# A Mammalian Conserved Circular RNA *CircLARP1B* Regulates Hepatocellular Carcinoma Metastasis and Lipid Metabolism

**DOI:** 10.1002/advs.202305902

**Published:** 2023-11-12

**Authors:** Jingxin Li, Xiaolin Wang, Liang Shi, Boqiang Liu, Zhiyong Sheng, Shuhui Chang, Xiujun Cai, Ge Shan

**Affiliations:** ^1^ Department of Laboratory Medicine The First Affiliated Hospital of USTC The CAS Key Laboratory of Innate Immunity and Chronic Disease School of Basic Medical Sciences Division of Life Science and Medicine University of Science and Technology of China Hefei 230027 China; ^2^ Department of General Surgery Sir Run Run Shaw Hospital School of Medicine Zhejiang University Hangzhou 310016 China; ^3^ School of Life Science Bengbu Medical College Bengbu 233030 China; ^4^ Department of Pulmonary and Critical Care Medicine Regional Medical Center for National Institute of Respiratory Diseases Sir Run Run Shaw Hospital School of Medicine Zhejiang University Hangzhou 310016 China

**Keywords:** *CircLARP1B*, conservation, HCC, HNRNPD, lipid metabolism, metastasis

## Abstract

Circular RNAs (circRNAs) have emerged as crucial regulators in physiology and human diseases. However, evolutionarily conserved circRNAs with potent functions in cancers are rarely reported. In this study, a mammalian conserved circRNA *circLARP1B* is identified to play critical roles in hepatocellular carcinoma (HCC). Patients with high *circLARP1B* levels have advanced prognostic stage and poor overall survival. *CircLARP1B* facilitates cellular metastatic properties and lipid accumulation through promoting fatty acid synthesis in HCC. *CircLARP1B* deficient mice exhibit reduced metastasis and less lipid accumulation in an induced HCC model. Multiple lines of evidence demonstrate that *circLARP1B* binds to heterogeneous nuclear ribonucleoprotein D (HNRNPD) in the cytoplasm, and thus affects the binding of HNRNPD to sensitive transcripts including *liver kinase B1* (*LKB1*) mRNA. This regulation causes decreased *LKB1* mRNA stability and lower LKB1 protein levels. Antisense oligodeoxynucleotide complementary to theHNRNPD binding sites in *circLARP1B* increases the HNRNPD binding to *LKB1* mRNA. Through the HNRNPD–LKB1–AMPK pathway, *circLARP1B* promotes HCC metastasis and lipid accumulation. Results from AAV8‐mediated hepatocyte‐directed knockdown of *circLARP1B* or Lkb1 in mouse models also demonstrate critical roles of hepatocytic *circLARP1B* regulatory pathway in HCC metastasis and lipid accumulation, and indicate that *circLARP1B* may be potential target of HCC treatment.

## Graphical abstract

The mammalian conserved *circLARP1B* promotes hepatocellular carcinoma metastasis and lipid accumulation. *CircLARP1B* functions by sequestering cytoplasmic heterogeneous nuclear ribonucleoprotein D (HNRNPD) to decrease HNRNPD binding to sensitive transcripts including *liver kinase B1* (*LKB1*) mRNA, leading to decreased *LKB1* mRNA stability and lower protein levels. Hepatocyte‐directed knockdown of *circLARP1B* or Lkb1 in mice impedes hepatocellular carcinoma metastasis and lipid accumulation.

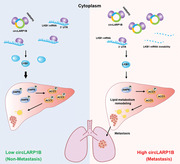

## 1 Introduction

Hepatocellular carcinoma (HCC) is the most common type of primary liver cancer, with high mortality rate and poor prognosis.^[^ ^1^, ^2^ ^]^ The five‐year overall survival rate exceeds 70% for early‐stage HCC, while the median survival of advanced (metastatic) HCC patients is ≈1.5 years.^[^ ^2^ ^]^ Metastatic HCC is less effective for surgical treatment and more resistant to drug therapy.^[^ ^3^, ^4^ ^]^ Metastasis is a pivotal process, frequently accompanied by dynamic metabolic alterations.^[^ ^5^, ^6^ ^]^ During the progress of metastasis, cancer cells utilize multiple mechanisms including lipid accumulation to favor cancer cell survival and provide energy to distant metastasis.^[^ ^7^ ^]^ Lipid metabolism reprogramming is actually another cancer hallmark, which is a complex cascade controlled by various factors, and AMP‐activated protein kinase (AMPK) and acetyl‐CoA carboxylase (ACC) are two key regulators among them.^[^ ^8^, ^9^, ^10^ ^]^ AMPK is a metabolic sensor in mammals activated by the increased cellular ratio of AMP/ATP.^[^ ^11^ ^]^ ACC is the rate‐limiting enzyme for fatty acid synthesis, and can be inhibited by AMPK.^[^ ^9^, ^11^, ^12^ ^]^

Circular RNAs (circRNAs) are a class of endogenous RNA transcripts, generated by back‐splicing from linear precursor RNAs or other RNA circularization mechanisms.^[^ ^13^, ^14^, ^15^, ^16^, ^17^ ^]^ Most circRNAs localize to the cytoplasm in an Exportin 4 dependent mechanism,^[^ ^18^ ^]^ and cytoplasmic circRNAs mainly function through sponging miRNAs, modulating RNA binding proteins (RBPs), and serving as translation templates.^[^ ^13^, ^19^, ^20^, ^21^, ^22^, ^23^ ^]^ Compiling studies have been carried out to investigate the molecular and cellular functions of circRNAs, although only a handful of circRNAs have been studied for the physiological roles with genetically manipulated mice, in which the expression of the corresponding circRNAs is missing or at a low level.^[^ ^13^, ^14^, ^15^ ^]^ The mammalian conserved circRNA *CDR1as* impacts brain development and function by sequestering *miR‐7*.^[^ ^19^, ^20^ ^]^ *CircBoule*, a circRNA conserved in metazoan, plays roles in the antistress reaction of sperm by regulating the stability of heat shock proteins.^[^ ^24^ ^]^ *Cia‐cGAS* regulates the differentiation of hematopoietic stem cells by binding and inhibiting cGAS in bone marrow.^[^ ^25^ ^]^

In cancers, an array of circRNAs have been shown to play regulatory roles.^[^ ^26^, ^27^ ^]^ For example, *circNSUN2* interacts with IGF2BP2 to form a circNSUN2‐IGF2BP2‐HMGA2 ternary complex to promote liver metastasis of colorectal cancer.^[^ ^21^ ^]^ The circRNA *FECR1* induces extensive DNA demethylation in the FLI1 genomic locus, thereby enhancing its expression to promote metastasis of breast cancer.^[^ ^28^ ^]^ *CircTP63* upregulates FOXM1 by competitively binding to *miR‐873‐3p* to facilitate cell cycle progression in lung squamous cell carcinoma.^[^ ^29^ ^]^ Multiple circRNAs have also been reported to play roles in HCC.^[^ ^23^, ^30^, ^31^ ^]^ For example, *circMTO1* suppresses HCC progression by serving as an *miR‐9* sponge to promote the expression of p21.^[^ ^30^ ^]^ *CircPABPC1* represses both intrahepatic and distant metastases in HCC through the degradation of ITGB1 in a ubiquitination‐independent manner.^[^ ^31^ ^]^ *Circβ‐catenin* promotes the HCC cell growth and metastasis via encoding a small protein, which stabilizes the full‐length β‐catenin to activate the Wnt pathway.^[^ ^23^ ^]^ CircRNAs can participate in HCC in multiple ways,^[^ ^23^, ^30^, ^31^ ^]^ although there is so far no circRNA identified to regulate HCC metastasis by modulating lipid metabolism.

Most of the studies in cancer circRNAs have been carried out with tumor cell lines, with associated clinical specimens and data, and sometimes with nude mice models.^[^ ^26^, ^27^, ^32^ ^]^ Lots of circRNAs with identified functions in cancer are not conserved between human and mice, which in some way hampers in‐depth investigations for roles of circRNAs and the underlying mechanisms with animal models. Identification of conserved circRNAs with critical roles in HCC yields meaningful insights into potential biomarkers and therapeutic targets.

In an effort to explore the roles of circRNAs in HCC metastasis, we have identified a mammalian conserved circRNA *circLARP1B*. With a series of bioinformatics, molecular, biochemical, and cellular analyses, and very importantly by using genetically modified mice, we have provided data to support roles of *circLARP1B* in HCC. We have uncovered that *circLARP1B* disturbs heterogeneous nuclear ribonucleoprotein D (HNRNPD) from the binding to 3′ UTR of *liver kinase B1* (*LKB1*), which leads to destabilizing *LKB1* mRNA and then modulating a pathway pivotal to HCC metastasis and lipid metabolism.

## 2 Results

### 2.1 *CircLARP1B* as a Mammalian Conserved CircRNA Is Identified in HCC Metastasis

**Figure 1:**
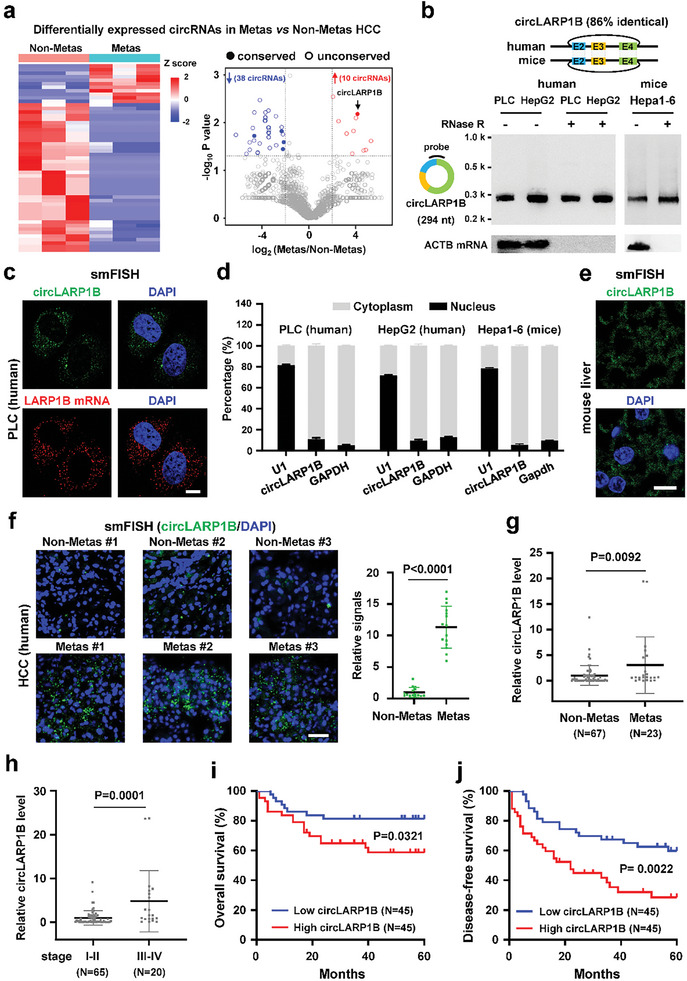
Characterization of mammalian‐conserved *circLARP1B* in HCC metastasis. a) Heatmap and volcano plot illustrating the differentially expressed circRNAs in three metastasis (metas) versus three nonmetastasis (nonmetas) specimens. In the volcano plot, red dots indicate significantly upregulated circRNAs, and blue ones represent downregulated circRNAs. Filled and empty dots indicate conserved and unconserved circRNAs, respectively. Specimens are in situ HCC from patients with or without metastasis. b) Northern blotting analysis of *circLARP1B* in human cells (PLC and HepG2) and murine Hepa1‐6 cells with or without RNase R treatment. The hybridization probe against the back‐splicing junction (BSJ) of *circLARP1B* is shown. *ACTB* mRNA is a control for the effect of RNase R, which digests linear RNAs. c) Representative smFISH images of *circLARP1B* (green) and *LARP1B* mRNA (red) in PLC cells. Blue, DAPI staining of nuclei. Scale bar: 10 µm. d) RT‐qPCR analysis of *circLARP1B* in the nuclear/cytoplasmic fraction of human cells (PLC and HepG2) and murine Hepa1‐6 cells. e) Representative smFISH images of *circLARP1B* (green) in the liver from wildtype mice. DAPI (nuclei, blue). Scale bar: 10 µm. f) RNA smFISH of *circLARP1B* (green) in nonmetas and metas specimens. Quantification of positive dots of *circLARP1B* smFISH is shown (right). *N* = 15 views. Scale bar: 10 µm. g,h) RT‐qPCR analysis of *circLARP1B* expressions in HCC specimens. *ACTB* mRNA is the endogenous control for normalization. i,j) Kaplan–Meier analysis of overall survival (i) and disease‐free survival (j) for 90 HCC patients collected. The red curve indicates survival in patients with higher HCC *circLARP1B* levels, and the blue line indicates survival in patients with lower HCC *circLARP1B* levels. (d) Data are shown as mean ± SD from three independent experiments. (f–h) *P*‐values are from two‐tailed unpaired Student's *t*‐test. (i,j) *P*‐values are calculated by the log‐rank test.

In order to investigate the functional roles of conserved circRNAs in HCC, we performed ribosomal RNA depleted RNA‐sequencing (RNA‐seq) of six HCC specimens, among which three HCC patients were metastatic and the other three were nonmetastatic (**Figure** **1**a). 4614 circRNAs with back‐splicing junction (BSJ) reads >2 were detected, and 48 of them were with over fourfold changes in expression levels between the two groups (Figure 1a; Table S1, Supporting Information). 38 of these circRNAs were with lower levels, and 10 out of the 48 were with higher levels in metastatic HCC (Figure 1a). Then we aligned the 48 circRNA sequences to annotated murine circRNAs from the circAtlas database to identify conserved circRNAs with a stringent criterion (full‐length and BSJ sequences >75% identical).^[^ ^33^ ^]^ *CircLARP1B* was the most differentially expressed and also the only metastasis upregulated circRNA among the five conserved circRNAs identified (Figure 1a). *CircLARP1B* is highly conserved with ≈86% sequence identity between human and mice, and consists of exons 2–4 from the La ribonucleoprotein 1B (*LARP1B)* gene, with a deduced size of 294 nt in both human and mice (Figure 1b; Figure S1a, Supporting Information). *CircLARP1B* and *LARP1B* mRNA exhibited distinct expression patterns in human tissues analyzed from the NCBI and circAtlas databases; for example, *LARP1B* mRNA but not *circLARP1B* was expressed in thyroid tissue (Figure S1b, Supporting Information). LARP1B itself is an RBP that regulates the translation of certain mRNAs.^[^ ^34^ ^]^ The overall survival rate showed no significant difference between the two groups of HCC patients with higher *LARP1B* expression and lower expression (Figure S1c, Supporting Information), indicating that *LARP1B* mRNA may not be a key regulator in HCC. The *circLARP1B* BSJ was validated by PCR amplification and confirmed by Sanger sequencing in a human HCC cell line PLC and mouse liver, to verify the circular nature of *circLARP1B* (Figure S1d, Supporting Information). Then, the actinomycin D chase assay revealed that *circLARP1B* was stable compared to *LARP1B* mRNA (Figure S1e, Supporting Information). By Northern blotting, bands corresponding to the deduced size of *circLARP1B* were detected, and the estimated copy numbers per cell in two HCC cell lines PLC and HepG2 were ≈136 and ≈158, respectively (Figure 1b; Figure S1f, Supporting Information). Copy numbers of more than 100 are high for circRNAs or even for mRNAs. Single molecular fluorescence in situ hybridization (smFISH) of *circLARP1B* in PLC cells revealed that this circRNA localized predominately in the cytoplasm (Figure 1c; Figure S1g,h, Supporting Information). The specificity of smFISH to *circLARP1B* was demonstrated with significantly decreased smFISH signals upon shcircLARP1B knockdown of the circRNA (Figure S1i–k, Supporting Information). With a thyroid cell line (K1 cells), which exhibited considerable *LARP1B* mRNA levels but no *circLARP1B* expression, the specificity of smFISH to *circLARP1B* or *LARP1B* mRNA was further proofed (Figure S1l, Supporting Information). More than 85% of *circLARP1B* were found to be cytoplasmic in one murine HCC cell line Hepa1‐6 and the two human HCC cell lines (Figure 1d). Furthermore, smFISH of *circLARP1B* in mouse liver also showed cytoplasmic enrichment (Figure 1e).

Higher *circLARP1B* levels in metastatic compared to nonmetastatic HCC specimens were revealed by smFISH analysis (Figure 1f). We collected HCC specimens from 90 patients, and found that higher *circLARP1B* levels were associated with 23 metastatic specimens as against the 67 nonmetastatic specimens (Figure 1g). Except 5 specimens without information of prognostic stage, these clinical samples could be divided into 65 specimens with prognostic TNM stage I–II and 20 specimens with TNM stage III–IV, and higher *circLARP1B* levels were significantly correlated with advanced TNM stages (III–IV) (Figure 1h). HCC patients with higher *circLARP1B* levels in tumors had significantly lower five‐year overall survival (OS) and disease‐free survival (DFS) rates (Figure 1i,j).

Collectively, these results demonstrated that *circLARP1B* as a mammalian conserved and highly expressed circRNA might play promoting roles in HCC metastasis as supported by data from clinical specimens.

### 2.2 *CircLARP1B* Regulates Cell Invasion and Lipid Accumulation via Fatty Acid Synthesis

**Figure 2:**
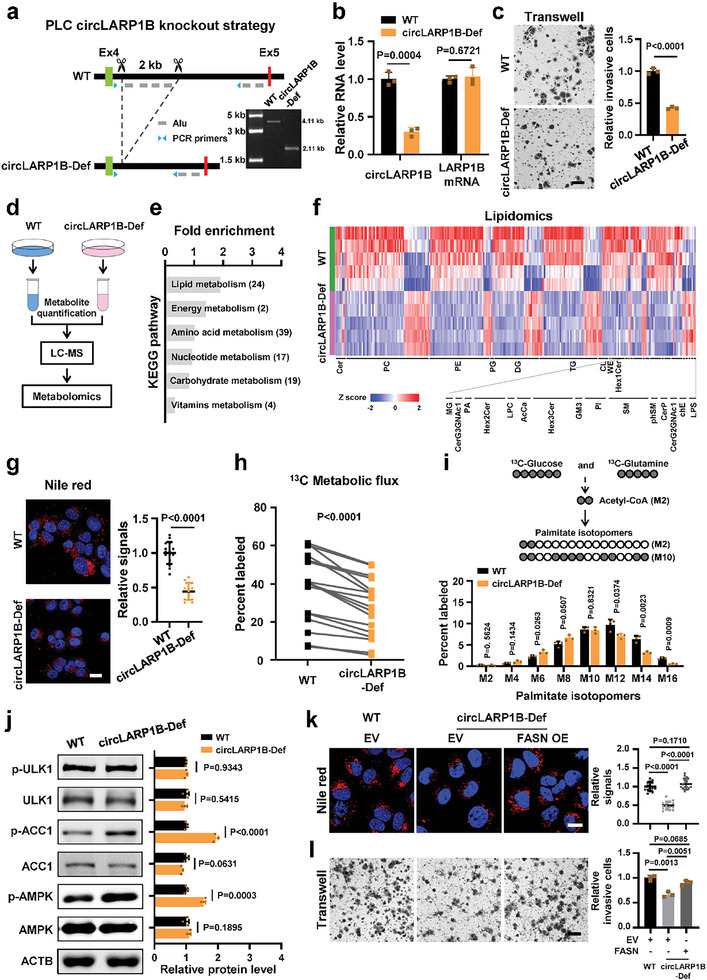
*CircLARP1B* modulates cell invasion and fatty acid synthesis in mammalian cells. a) Strategy of deleting intronic reverse‐complementary repeats in human *LARP1B* using CRISPR/Cas9 in PLC cells. Genomic PCR validation of WT and circLARP1B‐Def PLC cells is shown, and the corresponding amplifications are confirmed by Sanger sequencing. WT, wildtype; circLARP1B‐Def, *circLARP1B* deficient. b) RT‐qPCR analysis of *circLARP1B* and *LARP1B* mRNA expressions in WT and circLARP1B‐Def PLC cells. c) Transwell assays of WT and circLARP1B‐Def PLC cells. Quantification of invasive cells is shown (right). Scale bar: 100 µm. d) Schematic procedure of the untargeted metabolomics. All cells were maintained with standard DMEM medium containing 10% FBS for 48 h and then subjected to metabolomics analysis. e) KEGG pathway analysis of dysregulated metabolites in circLARP1B‐Def versus WT PLC cells. The number of significantly changed metabolites in the corresponding pathway is included in the brackets. f) Heatmap of the significantly changed lipid species (VIP >1, *P*‐value < 0.05) corresponding to 24 lipid classes enriched in lipid metabolism (e) from untargeted lipidomics of WT and circLARP1B‐Def PLC cells. VIP, variable importance in the projection. *N* = 5 samples for each cell type. g) Representative Nile red (red) and Hoechst 33342 (for nuclei, blue) staining of WT and circLARP1B‐Def PLC cells. *N* = 15. Scale bar: 20 µm. h) The distribution of ^13^C‐label fatty acids from ^13^C‐labeled targeted metabolic flux analysis of WT and circLARP1B‐Def PLC cells measured by liquid chromatography‐mass spectrometry (LC‐MS) following a 24 h ^13^C‐glucose and ^13^C‐glutamine incubation. A total of 17 ^13^C‐label fatty acids are detected and three replicates are performed for each group. i) Schematic depicting of the incorporation of uniformly labeled ^13^C‐glucose or ^13^C‐glutamine (^13^C is indicated by gray circle) into the fatty acid palmitate. Two representative palmitate isotopologs are indicated (top). The distribution of ^13^C‐label in even palmitate isotopologs in WT and circLARP1B‐Def PLC cells measured by LC‐MS is shown (bottom). j) Western blots and the quantification of p‐ULK1, ULK1, p‐ACC1, ACC1, p‐AMPK, and AMPK proteins in WT and circLARP1B‐Def PLC cells. ACTB, Actin b protein used as a loading control. The grayscale statistics of western blotting were performed by ImageJ. k) Representative Nile red (red) and Hoechst 33342 (nuclei, blue) staining of WT or circLARP1B‐Def PLC cells with overexpressing FASN (FASN OE) or not. EV, empty vector. *N* = 15. Scale bar: 20 µm. l) Transwell assays of WT or circLARP1B‐Def PLC cells with overexpressing FASN or not. Scale bar: 100 µm. (g,k) Nile red signal is defined as the mean gray value per cell quantified by ImageJ. (b,c,i,j,l) Data are shown as mean ± SD from three independent experiments. (b,c,g,i–l) *P*‐values by two‐tailed unpaired Student's *t*‐test. (h) *P*‐values by two‐tailed paired Student's *t*‐test.

To explore the cellular functions of *circLARP1B*, we applied the clustered regularly interspaced short palindromic repeats (CRISPR)/Cas9 technique to generate *circLARP1B* deficient (circLARP1B‐Def) PLC cells (**Figure** **2**a). Flanking introns of circularized exons contain *Alu* elements and *B1* repeats in human and mice, respectively, which both belong to reverse complementary sequences, known to facilitate circRNA biogenesis (Figure S2a, Supporting Information).^[^ ^13^, ^35^ ^]^ To generate circLARP1B‐Def PLC cells, repeat sequences including four proximal *Alu* elements in the fourth intron of human *LARP1B* were deleted, but functional intronic elements such as the splice sites and pyrimidine tract remained unaltered (Figure 2a). The resulted circLARP1B‐Def PLC cells exhibited significantly decreased *circLARP1B* levels with no alteration in *LARP1B* mRNA levels, compared to the wildtype (WT) PLC cells (Figure 2b).

CircLARP1B‐Def PLC cells compared to WT cells showed significantly reduced invasion, when examined with Transwell invasion assay (Figure 2c). No significant difference in growth curve and colony formation was observed between the circLARP1B‐Def and WT cells (Figure S2b–d, Supporting Information). Invasion ability is directly related to metastasis, and growth curve and colony formation are associated with cell growth and proliferation.

We then assessed the potential metabolic alterations in circLARP1B‐Def PLC cells by liquid chromatography‐mass spectrometry (LC‐MS) untargeted metabolomics (Figure 2d). A total of 366 metabolites and lipid classes (including 44 lipid classes) were detected, and 105 of them, 42 increased and 63 decreased, were significantly dysregulated (variable importance in the projection, VIP >1; *P*‐value <0.05) upon *circLARP1B* deficiency in PLC cells (Figure S2e; Table S2, Supporting Information). KEGG pathway analysis of these dysregulated metabolites revealed that lipid metabolism, among the 6 metabolic pathways significantly affected, was perturbed the most (Figure 2e). For the 24 lipid classes enriched in lipid metabolism of KEGG analysis, 18 ones were decreased, and 6 were increased in circLARP1B‐Def cells (Figure S2e, Supporting Information). We then performed lipidomics to examine lipid class composition, and identified 2709 lipid species that compose the 24 dysregulated lipid classes detected in the metabolomics. 273 lipid species covering all these 24 lipid classes were significantly changed upon *circLARP1B* deficiency (Figure 2f; Table S2, Supporting Information). Components of lipid droplets (LDs) including lipid classes such as phosphatidylcholine (PC), phosphatidylethanolamine (PE), diglyceride (DG), triglyceride (TG), and phosphatidylinositol (PI) were among the metabolites with lower levels in circLARP1B‐Def cells (Figure S2e, Supporting Information).^[^ ^36^ ^]^ LDs examined by Nile red staining were markedly decreased in circLARP1B‐Def cells compared to WT cells (Figure 2g).

To further explore the mechanism of lipid accumulation promoted by *circLARP1B*, we performed metabolic labeling with ^13^C‐labeled targeted metabolic flux analysis to assess the incorporation of ^13^C‐labeled glucose and glutamine into lipid in circLARP1B‐Def and WT PLC cells (Figure 2h,i).^[^ ^37^ ^]^ Targeted metabolic flux analyses were performed, and we totally detected 17 kinds of fatty acids with ^13^C labeling, and found that all of them were significantly reduced in circLARP1B‐Def cells (Figure 2h; Table S3, Supporting Information), indicating a promoting effect of *circLARP1B* in fatty acid synthesis (FAS). Glucose and glutamine are first converted to two‐carbon acetyl‐CoA, which serves as a substrate for ACC1 enzyme in FAS, and ^13^C‐labeled two‐carbon units from ^13^C‐glucose or ^13^C‐glutamine into palmitate can thus be examined to reveal FAS from glucose or glutamine. As expected, the distributions of ^13^C‐labeled palmitate isotopologs (M12, M14, and M16) in circLARP1B‐Def cells, compared to WT cells were significantly lower (Figure 2i). We further evaluated the involvement of *circLARP1B* in fatty acid oxidation (FAO) with oxygen consumption rate (OCR) assays after supplementing cells with exogenous free fatty acids (FFAs; palmitate was supplied). CircLARP1B‐Def cells showed similar OCR compared with WT cells (Figure S2f, Supporting Information). Unc‐51 like autophagy activating kinase 1 (ULK1) is a key regulator in lipophagy initiation, and phosphorylation of Ser555 of ULK1 induces lipophagy.^[^ ^38^ ^]^ The ULK1 and phosphorylated ULK1 (p‐ULK1) levels exhibited no significant alternation between circLARP1B‐Def and WT cells (Figure 2j). Together, these findings suggested that *circLARP1B* facilitated lipid accumulation by FAS, not through regulating FAO or lipophagy.

The activity of ACC1 is inhibited by phosphorylation of Ser79,^[^ ^9^, ^11^ ^]^ and we observed that the phosphorylated ACC1 (p‐ACC1) levels were substantially upregulated in circLARP1B‐Def cells (Figure 2j). AMPK is the major kinase that catalyzes the phosphorylation of ACC1, and AMPK itself is initially activated by phosphorylation at Thr172.^[^ ^11^ ^]^ The phosphorylated AMPK (p‐AMPK) levels were also significantly higher in circLARP1B‐Def cells than WT cells (Figure 2j). Therefore, higher levels of p‐AMPK and then p‐ACC1 would repress lipogenesis and lead to decreased amount of LDs in circLARP1B‐Def cells. AMPK activation also causes a range of metabolites changed in HCC.^[^ ^39^, ^40^, ^41^ ^]^ From the literature we searched, 12 of circLARP1B‐modulated metabolites have been reported to be regulated by AMPK signaling in HCC,^[^ ^39^, ^40^, ^41^ ^]^ and 8 of these metabolites exhibit consistent changes between *circLARP1B* deficiency and AMPK activation (Figure S2g, Supporting Information).

Additionally, we examined the effects of *circLARP1B* on the other downstream processes known to be regulated by AMPK, such as the mTOR pathway, angiogenesis, TGF‐β, and mitochondrial activity.^[^ ^11^, ^42^, ^43^, ^44^ ^]^ AMPK activation led to phosphorylation of the mTORC1 subunit RAPTOR to inhibit mTOR pathway,^[^ ^11^ ^]^ and significantly increased phosphorylated RAPTOR (p‐RAPTOR) levels were observed in circLARP1B‐Def cells compared to WT cells (Figure S2h, Supporting Information). Once activated, AMPK is reported to increase the protein level of VEGFA (an angiogenesis driver) and inhibit TGF‐β signaling,^[^ ^11^, ^42^, ^43^ ^]^ and the VEGFA and TGF‐β protein levels did not demonstrate significant change in circLARP1B‐Def cells compared to WT cells (Figure S2h, Supporting Information). As another substrate of AMPK, mitochondrial fission factor (MFF) is phosphorylated by AMPK to promote mitochondrial fission,^[^ ^11^ ^]^ and the MFF/p‐MFF protein levels remained unaltered upon *circLARP1B* deficiency in PLC cells (Figure S2h, Supporting Information). Moreover, basal respiration, ATP production, and maximum respiration revealing mitochondrial activity examined by OCR assays demonstrated no significant difference between circLARP1B‐Def cells and WT cells (Figure S2i, Supporting Information). Our results demonstrated that both ACC1 and the mTOR signaling directly regulated by AMPK were modulated by *circLARP1B* through a mechanism awaiting characterization in PLC cells. Both ACC1 and the mTOR signaling are known to promote fatty acid synthesis,^[^ ^9^, ^44^ ^]^ we focused on examining ACC1/p‐ACC1 as a readout of downstream effects of AMPK, owing to the fact that ACC1 is the rate‐limiting enzyme for FAS.^[^ ^9^ ^]^

We also manipulated levels of *circLARP1B in trans* by either RNA interference knockdown or plasmid overexpression, and in human PLC cells or murine Hepa1‐6 cells. ShRNA‐mediated *circLARP1B* knockdown suppressed cell invasion and formation of LDs, and increased p‐AMPK and p‐ACC1 levels in both PLC and Hepa1‐6 cells (Figure S3a–h, Supporting Information). *CircLARP1B* overexpression promoted cell invasion and LDs formation, and decreased the p‐AMPK and p‐ACC1 levels in PLC cells (Figure S3i–l, Supporting Information).

Roles of lipid metabolism in the promoting effects of *circLARP1B* on HCC cell invasion were then investigated. Fatty acid synthase (FASN), which is the human lipogenic enzyme for de novo fatty acid synthesis,^[^ ^9^ ^]^ was overexpressed in circLARP1B‐Def PLC cells (Figure 2k,l). FASN overexpression enhanced formation of LDs in circLARP1B‐Def PLC cells, and rescued the inhibitory effect on cell invasion caused by *circLARP1B* deficiency (Figure 2k,l). Then, we treated circLARP1B‐overexpressing PLC cells with IPI‐9119, a potent FASN inhibitor,^[^ ^45^ ^]^ and found that FASN inhibition blocked the promoting effect of *circLARP1B* on the formation of LDs and cell invasion (Figure S3m,n, Supporting Information).

Taken together, these findings demonstrated that *circLARP1B* stimulated HCC invasion through remodeling lipid metabolism at the cellular level, and promoted FAS via modulating the AMPK and its downstream targets including ACC1.

### 2.3 *CircLARP1B* Interacts with HNRNPD Protein

We set out to elucidate the functional mechanism of *circLARP1B* through inspecting its potential interacting molecules. Several putative miRNA binding sites on *circLARP1B* were predicted using CircInteractome (Figure S4a, Supporting Information),^[^ ^46^ ^]^ however, RNA immunoprecipitation (RIP) of AGO2, which mediates the interaction between target RNA and miRNA,^[^ ^13^ ^]^ showed no enrichment of *circLARP1B* in PLC cells (Figure S4b, Supporting Information). Furthermore, we applied ribosome profiling assay and found no obvious binding of *circLARP1B* to polysomes (Figure S4c, Supporting Information), indicating that *circLARP1B* did not function as a template for protein translation. Both human and murine *circLARP1B* also displayed low coding possibilities in CPAT predicting tool (Figure S4d, Supporting Information).^[^ ^47^ ^]^ Therefore, *circLARP1B* is noncoding and does not function as miRNA sponge.

**Figure 3:**
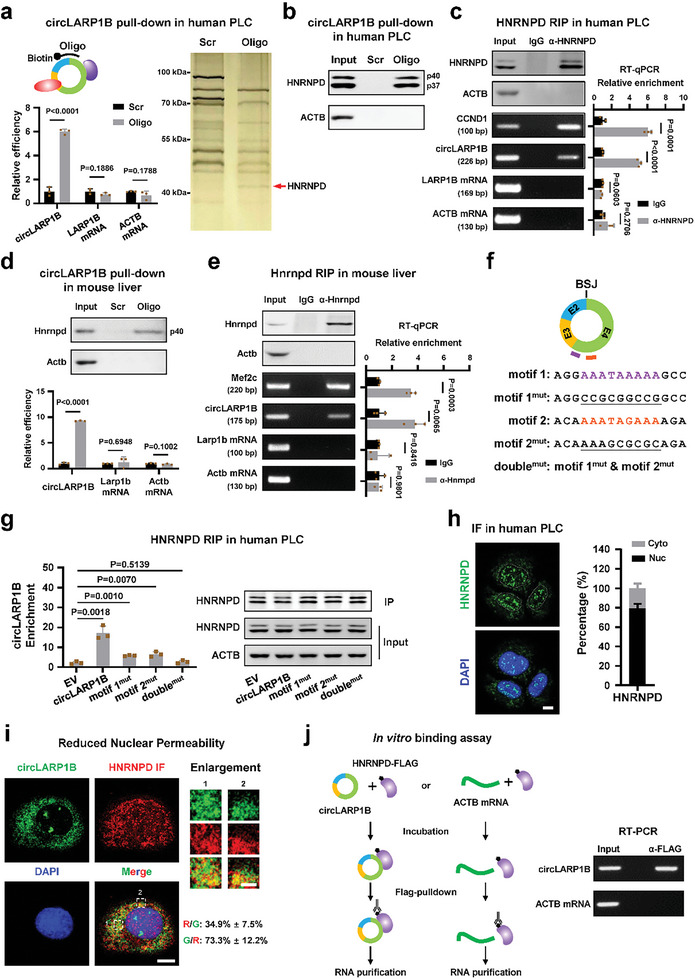
*CircLARP1B* interacts with HNRNPD via two binding sites of the circRNA. a) Pull‐down efficiency of *circLARP1B* in the cytoplasmic fraction of PLC cells (left). Biotinylated oligonucleotide antisense to the *circLARP1B* back‐splicing junction (BSJ) is indicated. Silver staining shows the proteins co‐pulled down. The red arrow denotes the band identified as HNRNPD by mass spectrometry. Scr, negative control of biotin‐labeled oligo with scrambled sequences; Oligo, biotin‐labeled oligo with antisense sequences to the *circLARP1B* BSJ. The pull‐down efficiency of *circLARP1B* is shown with bar figure. b) Western blot of HNRNPD to demonstrate that RNA pull‐down of *circLARP1B* co‐pulls down HNRNPD in the cytoplasmic fraction of PLC cells. ACTB is a negative control. c) RIP with an antibody against HNRNPD in the cytoplasmic fraction of PLC cells pulls down *circLARP1B*. Western blots showing efficient pull‐down of HNRNPD with ACTB as a negative control. Gel images demonstrate the semiquantitative RT‐PCR products of RIP RNAs. RT‐qPCR analyses show the enrichment of RIP RNAs. *CCND1* mRNA is a known target of HNRNPD and a positive control.^[^ ^50^ ^]^ *ACTB* mRNA is a negative control. d,e) *circLARP1B* RNA pull‐down (d) and Hnrnpd RIP with the cytoplasmic fraction (e) using mouse liver. *Mef2c* mRNA is a known target of Hnrnpd in mice and a positive control examined in Hnrnpd RIP (e).^[^ ^94^ ^]^ f) Conserved binding motifs of HNRNPD in *circLARP1B*. The conserved motif 1 (purple) and motif 2 (orange) are mutated separately to motif 1^mut^ and motif 2^mut^, or mutated together as double^mut^. Both sites (Positions 143–151 and 158–166 from the BSJ) are located in the exon 4 of the *LARP1B* gene. g) Association of *circLARP1B* examined by RT‐qPCR of HNRNPD RIP in PLC cells with overexpression of the corresponding forms of *circLARP1B*. Enrichment, normalized to IgG. EV, empty vector. Western blot images indicate successful IP of HNRNPD protein. h) Immunofluorescence (IF) of HNRNPD (green) in PLC cells. Nuclei were stained with DAPI (blue). Quantification of nuclear (Nuc)/cytoplasmic (Cyto) HNRNPD signals is shown as bar figure. *N* = 15. Scale bar: 10 µm. i) Representative images of *circLARP1B* FISH together with HNRNPD IF in PLC cells. A permeabilization condition was used to reduce the nuclear HNRNPD signals,^[^ ^59^, ^60^ ^]^ thereby highlighting cytoplasmic signals in PLC cells. Boxed areas are enlarged. The colocalization between *circLARP1B* (G, green) and HNRNPD (R, red) is shown (*N* = 15 randomly selected areas). R/G, proportion of red signal to green signal colocalization; G/R, proportion of green signal to red signal colocalization. IF, immunofluorescence. Scale bar: 10 and 1 µm (enlarged areas). j) In vitro binding assay of synthesized *circLARP1B* and FLAG‐tagged HNRNPD protein purified from HEK293 cells. An illustration of the assay was shown (left) and semiquantitative RT‐PCR for *circLARP1B* or *ACTB* mRNA fragment was indicated (right). (a–e,g) Data are shown as mean ± SD from three independent experiments; *P*‐values by two‐tailed unpaired Student's *t*‐test.

We then performed RNA pull‐down with the cytoplasmic fraction of PLC cells with a biotinylated oligo against the BSJ of *circLARP1B*, which showed effective and specific capture of *circLARP1B* (**Figure** **3**a). Cytoplasmic fraction was used due to the predominately cytoplasmic localization of *circLARP1B* (Figure 1c–e). The proteins co‐pulled down with *circLARP1B* were separated with SDS‐PAGE, followed by silver staining and mass spectrometry (MS) to disclose the specific *circLARP1B* binding band (Figure 3a). HNRNPD, also known as AU‐rich element RNA‐binding factor 1 (AUF1), was identified as the circLARP1B‐interacting protein in human cells (Figure 3a; Figure S4e, Supporting Information). HNRNPD is an RBP with both cytoplasmic and nuclear roles.^[^ ^48^, ^49^ ^]^ In the cytoplasm, HNRNPD regulates the stability of some mRNAs, e.g., *CCND1* mRNA, through binding to the AU‐rich region in 3′ UTR.^[^ ^48^, ^49^, ^50^ ^]^ HNRNPD undergoes alternative splicing to produce four protein variants (p37, p40, p42, and p45) that are expressed with cell type‐specificity,^[^ ^49^, ^51^ ^]^ and two variants (p37 and p40) were further confirmed as major HNRNPD isoforms in PLC cells (Figure S4f, Supporting Information). RNA pull‐down of *circLARP1B* and HNRNPD RIP assays using the cytoplasmic fraction of PLC cells further verified their interaction (Figure 3b,c). Using mouse liver as the experimental material, RNA pull‐down of murine *circLARP1B* could co‐pull down Hnrnpd, and Hnrnpd RIP acquired *circLARP1B* (Figure 3d,e). Therefore, *circLARP1B* and HNRNPD interact in both human and murine cells.

To map the binding sites of HNRNPD in *circLARP1B*, we uncovered two motifs (motif 1 and motif 2) conserved in human and murine *circLARP1B* via RBPmap (Figure 3f), a tool for predicting RBP binding sites in an RNA sequence.^[^ ^52^ ^]^ Both sites (Positions 143–151 and 158–166 from BSJ) are also AU‐rich, consistent with the HNRNPD binding preference to AU‐rich region.^[^ ^49^ ^]^ Mutation of either motif 1 or motif 2 in *circLARP1B* decreased the interaction between *circLARP1B* and HNRNPD in PLC cells (Figure 3g). Mutation of both motifs (double^mut^) abolished the interaction (Figure 3g). The 5′ portion of *LARP1B* mRNA possessing the same nucleic acid sequences as *circLARP1B* also has the HNRNPD binding site sequences present in *circLARP1B*, although HNRNPD RIP followed by semiquantitative RT‐PCR and real‐time quantification PCR (RT‐qPCR) analyses revealed that HNRNPD did not bind to *LARP1B* mRNA in PLC cells and mouse liver (Figure 3c,e; Figure S5a, Supporting Information). The 5′ portion of *LARP1B* mRNA is heavily bound by ribosomes through analyzing available ribosome profiling sequencing (Ribo‐seq) data from HCC cell lines (GSE125757, GSE128320, and GSE147840) (Figure S5b, Supporting Information).^[^ ^53^, ^54^, ^55^ ^]^ One possibility is that *LARP1B* mRNA is actively involved in translation, and the HNRNPD sites present in its 5′ are thus not available for HNRNPD binding.

HNRNPD binding to mRNA would modulate RNA stability,^[^ ^48^, ^50^, ^56^ ^]^ and we found out that knocking down of HNRNPD with siRNA did not affect levels of *circLARP1B*, indicating that HNRNPD did not regulate the stability of *circLARP1B* (Figure S5c, Supporting Information). Knocking down of *circLARP1B* with shRNA also did not affect levels of *HNRNPD* mRNA and protein (Figure S5d,e, Supporting Information). Consistent with previous reports,^[^ ^56^, ^57^, ^58^ ^]^ immunofluorescence (IF) analysis demonstrated that most HNRNPD localized in the nucleus, and ≈20% HNRNPD localized in the cytoplasm in PLC cells, which affected better visualization of cytoplasmic HNRNPD signals (Figure 3h). By using a condition to reduce the nuclear permeability to decrease the nuclear signals and thus to highlight the cytoplasmic HNRNPD signals,^[^ ^59^, ^60^ ^]^ we observed that more than 70% *circLARP1B* signals overlapped with HNRNPD protein signals in the cytoplasm (Figure 3i). A binding assay with FLAG‐tagged HNRNPD protein purified from HEK293 cells and synthesized *circLARP1B* RNA demonstrated the direct binding between *circLARP1B* and HNRNPD in vitro (Figure 3j). With over ≈100 copies in the cytoplasm, *circLARP1B* with two functional sites can bind to a substantial portion of cytoplasmic HNRNPD protein. Collectively, we concluded that *circLARP1B* with two functional sites and reasonable abundance could interact with a substantial portion of cytoplasmic HNRNPD, and *circLARP1B* and HNRNPD did not reciprocally regulate their expression or stability.

### 2.4 HNRNPD and HNRNPD Binding Are Essential for *CircLARP1B* Functions

**Figure 4:**
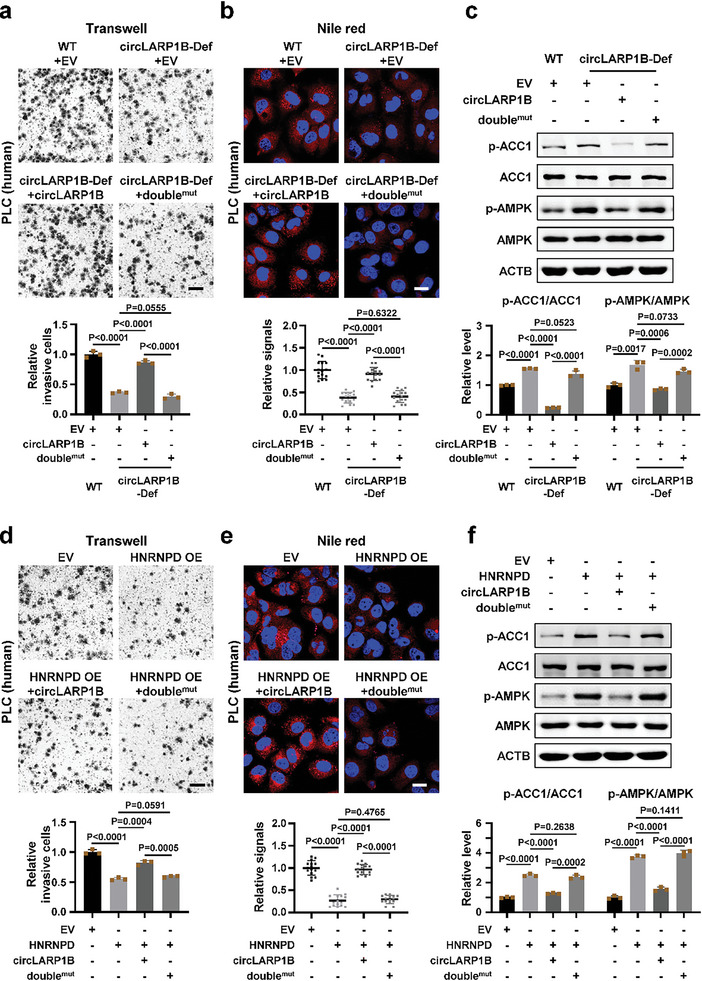
Effects of *circLARP1B* depend on the interaction with HNRNPD. a) Transwell assays of WT or circLARP1B‐Def PLC cells with overexpressing *circLARP1B* or double^mut^ *circLARP1B*. The quantification of invaded cells is analyzed. double^mut^, *circLARP1B* two HNRNPD‐binding sites mutated. Scale bar: 100 µm. b) Representative Nile red (red) and Hoechst 33342 (for nuclei, blue) staining of WT or circLARP1B‐Def PLC cells with overexpressing *circLARP1B* or double^mut^ *circLARP1B*. *N* = 15. Scale bar: 20 µm. c) Western blot images and the quantification of the indicated proteins in WT or circLARP1B‐Def PLC cells with the overexpression of *circLARP1B* or double^mut^. d) Transwell assays in PLC cells with HNRNPD overexpression. Cells with HNRNPD overexpression are examined upon co‐overexpression of *circLARP1B* or double^mut^ *circLARP1B*. The quantification of invaded cells is shown. Scale bar: 100 µm. e) Representative Nile red (red) and Hoechst 33342 (for nuclei, blue) staining in PLC cells with HNRNPD overexpression. Cells with HNRNPD overexpression are examined upon co‐overexpression of *circLARP1B* or double^mut^ *circLARP1B*. *N* = 15. Scale bar: 20 µm. f) Western blot images and quantification of the proteins in PLC cells overexpressed HNRNPD, with co‐overexpression of *circLARP1B* or double^mut^. (b,e) The Nile red signal is defined as the mean gray value per cell quantified by ImageJ. (c,f) The grayscale statistics of western blotting was performed by ImageJ. (a,c,d,f) Data are shown as mean ± SD from three independent experiments. (a–f) *P*‐values by two‐tailed unpaired Student's *t*‐test. EV, empty vector.

We then set out to investigate the roles of HNRNPD binding in the functionality of *circLARP1B*. Overexpression of WT but not double^mut^ *circLARP1B* could rescue the phenotypes in cell invasion and formation of LDs of circLARP1B‐Def cells (**Figure** **4**a,b; Figure S5f, Supporting Information). At the molecular level, overexpression of WT but not double^mut^ *circLARP1B* restored the p‐AMPK and p‐ACC1 levels in circLARP1B‐Def cells (Figure 4c). Overexpression of HNRNPD inhibited cell invasion and formation of LDs (Figure 4d,e; Figure S5g, Supporting Information), and levels of p‐AMPK and p‐ACC1 were also increased in PLC cells (Figure 4f). WT but not double^mut^ *circLARP1B* that overexpressed together with HNRNPD blocked the effects of HNRNPD on cell invasion, formation of LDs, and levels of p‐AMPK and p‐ACC1 (Figure 4d–f). Taking these results together, we concluded that the interaction between HNRNPD and *circLARP1B* was essential to regulatory functions of this circRNA, and it is possible that *circLARP1B* might function through binding to and modulating HNRNPD.

### 2.5 *CircLARP1B* Destabilizes *LKB1* mRNA via Perturbing HNRNPD

**Figure 5:**
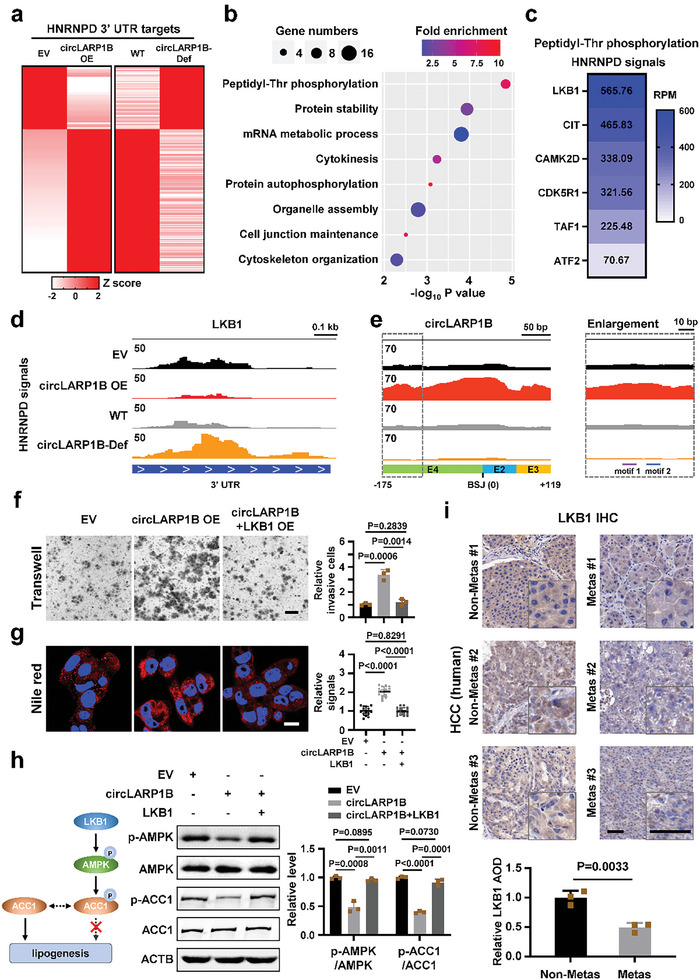
*LKB1* mRNA is a functional downstream target of *circLARP1B*. a) Heatmap showing HNRNPD RIP‐seq signals on the 3′ UTRs of 216 mRNAs that demonstrate significant sensitivity to *circLARP1B* levels. EV, PLC cells transfected with empty vector; *circLARP1B* OE, cells with *circLARP1B* overexpression; WT, wildtype PLC cells; circLARP1B‐Def, *circLARP1B* deficient PLC cells. b) Gene ontology (GO) analysis of 216 HNRNPD targets significantly sensitive to changes in *circLARP1B* levels. c) Total HNRNPD binding signals of four PLC cells (EV, *circLARP1B* OE, WT, circLARP1B‐Def) for six genes enriched in the biological process of peptidyl‐Thr phosphorylation. d) HNRNPD binding signals on *LKB1*’s 3′ UTR in four PLC cells. e) HNRNPD binding signals on *circLARP1B* (All reads including the BSJ reads are counted) in four PLC cells. Enlarged IGV visualization demonstrated HNRNPD binding signals around two functional motifs. f) Transwell assays of PLC cells with *circLARP1B* overexpression alone or with *circLARP1B* and LKB1 overexpression together. The quantification of invaded cells by ImageJ is shown. Scale bar: 100 µm. g) Representative Nile red (red) and Hoechst 33342 (nuclei, blue) staining of PLC cells with *circLARP1B* overexpression or co‐overexpressing of *circLARP1B* and LKB1. Nile red signal is defined as the mean gray value per cell quantified by ImageJ. *N* = 15. Scale bar: 20 µm. h) Western blot images and quantification of proteins in PLC cells with *circLARP1B* overexpression or co‐overexpressing of *circLARP1B* and LKB1. The grayscale statistics of western blotting was performed by ImageJ. i) Representative immunohistochemistry (IHC) staining and the quantification of LKB1 proteins in nonmetas and metas HCC specimens (*N* = 3). The IHC signal is defined as the average optical density (AOD) calculated by ImageJ. Scale bar: 50 µm. (f–h) EV, empty vector control. (f,h) Data are shown as mean ± SD from three independent experiments. (f–i) *P*‐values by two‐tailed unpaired Student's *t*‐test.

To provide further insights into the molecular mechanisms of *circLARP1B* and HNRNPD in HCC cells, we extracted cytoplasmic fractions of PLC cells and performed HNRNPD RIP of endogenously expressed HNRNPD, and the RNAs from RIP were subjected to RNA‐seq (RIP‐seq) (Figure S6a,b, Supporting Information). Distribution of RIP‐seq reads on mRNA targets revealed predominate 3′ UTR counts (Figure S6a, Supporting Information), consistent with the functional roles of cytoplasmic HNRNPD in regulating mRNA stability by binding to 3′ UTR.^[^ ^48^, ^50^, ^56^ ^]^ Comparison of RIP‐seq data from circLARP1B‐Def cells and *circLARP1B* overexpression cells to those from the corresponding control cells, identified 216 mRNAs with contrast changes of HNRNPD bindings on their 3′ UTR upon *circLARP1B* deficiency or overexpression (**Figure** **5**a; Table S4, Supporting Information). We have reanalyzed the HNRNPD PAR‐CLIP data (GSE52977) from HEK293 cells,^[^ ^61^ ^]^ and found that the 216 mRNAs sensitive to *circLARP1B* had significantly lower HNRNPD PAR‐CLIP binding signals in the 3′ UTRs, compared to the other HNRNPD PAR‐CLIP targets (Figure S6c, Supporting Information). These results indicated that the subset of mRNAs impacted by changes in *circLARP1B* levels might be with disadvantage in competing for the pool of HNRNPD, due to either relatively weaker binding ability to the protein or unfavorable cellular distribution and/or ratio of the corresponding mRNA/HNRNPD protein.

Gene ontology (GO) analysis for these target genes illustrated biological processes such as peptidyl‐Thr phosphorylation, protein stability, and mRNA metabolic process (Figure 5b). Peptidyl‐Thr phosphorylation was the most significantly (with the lowest *P*‐value) enriched process, among which LKB1 also known as serine/threonine kinase 11 (STK11) showed the most overall HNRNPD binding signals (the sum of binding signals from all four types of cells) on the 3′ UTR (Figure 5c). This indicated that its 3′ UTR occupied the most HNRNPD protein out of the 3′ UTRs of the six genes enriched in this GO term. With mouse liver, we found that Hnrnpd RIP could also pull down *Lkb1* mRNA (Figure S6d, Supporting Information). LKB1 is well known to directly catalyze the phosphorylation of Thr172 of AMPK, which is required for AMPK activation.^[^ ^62^, ^63^, ^64^ ^]^ Directly based on the RIP‐seq data, *circLARP1B* deficiency resulted in more HNRNPD bindings, and *circLARP1B* overexpression led to less HNRNPD bindings, to the 3′ UTR of *LKB1* mRNA (Figure 5d). We also found that the endogenous HNRNPD protein bound more *circLARP1B* upon *circLARP1B* overexpression and less *circLARP1B* in circLARP1B‐Def cells (Figure 5e). Furthermore, HNRNPD RIP revealed that *circLARP1B* deficiency but not knockdown of *LARP1B* mRNA resulted in significantly more HNRNPD binding to the 3′ UTR of *LKB1* mRNA (Figure S6e–g, Supporting Information). A recent study demonstrated that HNRNPD bound to the *c‐MYC* 3′ UTR and promoted the c‐MYC expression in colorectal cancer.^[^ ^65^ ^]^ HNRNPD RIP‐qPCR revealed that HNRNPD also bound to the *c‐MYC* 3′ UTR, and siRNA‐mediated HNRNPD knockdown significantly increased the c‐MYC protein levels in PLC cells (Figure S6h,i, Supporting Information). c‐MYC was not in the 216 targets sensitive to *circLARP1B* (Figure 5a; Table S4, Supporting Information), and the opposite effect of HNRNPD on c‐MYC expression in colorectal cancer and HCC cells required further investigation.

We examined the effect of LKB1 in HCC cells directly by overexpression, and we found that the overexpressed LKB1 blocked the effect of *circLARP1B* in promoting cell invasion and formation of LDs (Figure 5f,g). Overexpressed LKB1 also nearly abolished the effects of *circLARP1B* on p‐AMPK and p‐ACC1 levels (Figure 5h). Lower LKB1 protein levels were observed in metastatic compared to nonmetastatic HCC specimens (Figure 5i). These results indicated that *LKB1* mRNA was a downstream target of *circLARP1B* with robust contributions to the effects of *circLARP1B*.

### 2.6 *CircLARP1B* Competes with *LKB1* mRNA for HNRNPD Binding and Leads to *LKB1* mRNA Instability

**Figure 6:**
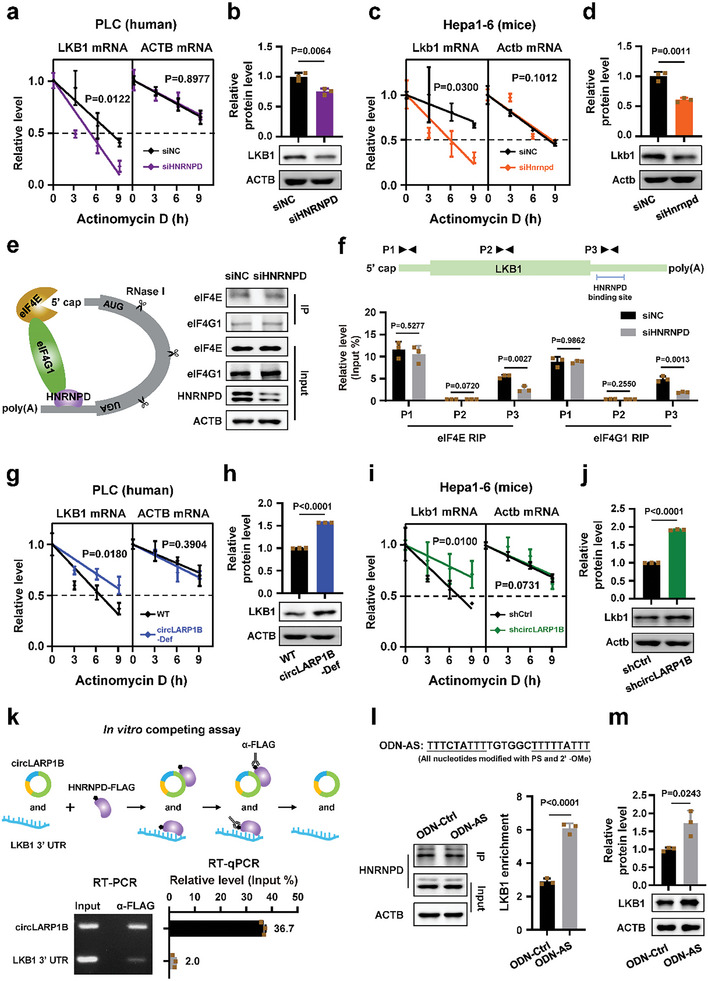
*CircLARP1B* destabilizes *LKB1* mRNA via perturbing HNRNPD. a–d) Stability assay of *LKB1* mRNA and the steady levels of LKB1 protein (examined by western blotting) in human PLC cells (a,b) and murine Hepa1‐6 cells (c,d) treated siRNA against HNRNPD. siNC, negative control siRNA with scrambled sequences. e) Overall experimental strategy of eIF4E and eIF4G1 RIP assays. RNase I is introduced to digest unprotected RNAs across the IP procedure. Western blots showing HNRNPD knockdown efficiency and efficient IPs of eIF4E and eIF4G1 in PLC cells treated with siRNA against HNRNPD (right). ACTB protein acted as the loading control. f) Primers against various regions of *LKB1* mRNA (top) and enrichment of *LKB1* mRNA regions with eIF4E or eIF4G1 by RT‐qPCR (bottom). g,h) Stability assay of *LKB1* mRNA (g) and the steady levels of LKB1 protein (h) in WT or circLARP1B‐Def PLC cells. i,j) Stability assay of *Lkb1* mRNA (i) and the steady levels of Lkb1 protein (j) in Hepa1‐6 cells treated with shcircLARP1B or shCtrl. shCtrl, shRNA control that generates siRNA of scrambled sequences; shcircLARP1B, shRNA against the murine *circLARP1B* BSJ. k) In vitro competing assay of purified HNRNPD protein for equal moles of in vitro synthesized *circLARP1B* and *LKB1* 3′ UTR. An illustration of the assay was shown (top). Semiquantitative RT‐PCR gels and RT‐qPCR for *circLARP1B* and *LKB1* 3′ UTR were indicated (bottom). *LKB1* 3′ UTR, in vitro transcribed *LKB1* 3′ UTR fragments with the HNRNPD binding sequence. l) Association of *LKB1* mRNA examined by RT‐qPCR of HNRNPD RIP in PLC cells treated with oligodeoxynucleotide antisense to two HNRNPD binding motifs in *circLARP1B* (ODN‐AS) or the control (ODN‐Ctrl, ODN with scrambled sequences). Two motifs with reverse complementary sequences were indicated with underlines. PS, phosphorothioate; 2′‐OMe, 2′‐*O*‐methyl. Western blot images indicate successful IP of HNRNPD protein. Enrichment, normalized to IgG. m) Western blotting and the corresponding quantification showing the steady level of LKB1 protein in PLC cells transfected with ODN‐AS or ODN‐Ctrl. (b,d,h,j,m) The grayscale statistics of western blotting were performed by ImageJ. (a–d,f–m) Data are shown as mean ± SD from three independent experiments. (a,c,g,i) *P*‐values by two‐way ANOVA test. (b,d,f,h,j,l,m) *P*‐values by two‐tailed unpaired Student's *t*‐test.

HNRNPD has complex regulatory effects on the stability of mRNAs, with some stabilized and the others destabilized.^[^ ^48^, ^50^, ^56^ ^]^ We observed that the steady levels but not nascent levels of *LKB1* mRNA were significantly increased, upon *circLARP1B* deficiency or knockdown in human and murine cells (Figure S6j,k, Supporting Information). We then examined whether and how HNRNPD and *circLARP1B* regulated the steady levels of *LKB1* mRNA. Knocking down HNRNPD with siRNA resulted in significant decrease in half‐life of *LKB1* mRNA and also LKB1 protein levels in PLC and Hepa1‐6 cells (**Figure** **6**a–d), indicating that HNRNPD stabilized *LKB1* mRNA. HNRNPD has been reported to bind to the 3′ UTR and simultaneously interact with translation initiation factor eIF4G1 and cap‐binding protein eIF4E to form a ternary complex, which could bring the 5′ and 3′ ends of the mRNA together to form a loop.^[^ ^51^ ^]^ We wondered whether HNRNPD looped *LKB1* mRNA through this mechanism. We performed eIF4G1 and eIF4E RIP followed by RT‐qPCR with specific primers to detect the 3′ UTR of *LKB1* mRNA in PLC cells with or without HNRNPD knockdown.^[^ ^66^, ^67^ ^]^ HNRNPD knockdown resulted in decreased binding signal of both eIF4G1 and eIF4E to the *LKB1* 3′ UTR (Figure 6e,f). These results demonstrated that HNRNPD enhanced the mRNA looping and stabilized *LKB1* mRNA.

In circLARP1B‐Def PLC cells, compared to the WT cells, *LKB1* mRNA stability was increased, and the steady levels of LKB1 protein were also increased (Figure 6g,h). Overexpression of *circLARP1B* in PLC cells destabilized *LKB1* mRNA, and led to lower levels of LKB1 protein (Figure S6l,m, Supporting Information). In mouse Hepa1‐6 cells, the stability of *Lkb1* mRNA was markedly enhanced, and the steady levels of Lkb1 protein were significantly increased upon *circLARP1B* knockdown with shRNA (Figure 6i,j). Dual‐luciferase reporter using the 3′ UTR of *LKB1* mRNA as the 3′ UTR of firefly luciferase mRNA showed that WT *circLARP1B* but not the double^mut^ *circLARP1B* could partially block the promoting effect on HNRNPD‐mediated stabilization of *LKB1* 3′ UTR (Figure S7a, Supporting Information). Fluorescent in situ hybridization (FISH) of *LKB1* mRNA and the HNRNPD IF staining revealed that circLARP1B‐Def compared to WT PLC cells, exhibited significantly more colocalization of *LKB1* mRNA signals and HNRNPD protein signals in the cytoplasm (Figure S7b, Supporting Information). ≈459 and ≈663 copies of *LKB1* mRNA per cell were estimated in PLC and HepG2 cells, respectively (Figure S7c, Supporting Information). The cytoplasmic copies of *circLARP1B* and *LKB1* mRNA were evaluated in PLC cells, and the molecular ratio was ≈1:3 (Figure S7d, Supporting Information). A competing assay, in which purified HNRNPD was incubated with equal moles of in vitro synthesized *circLARP1B* and *LKB1* 3′ UTR, was set up (Figure 6k). It was found that HNRNPD had much higher (≈18‐fold) binding affinity to *circLARP1B* than to the 3′ UTR of *LKB1* mRNA (Figure 6k).

A 24‐nt antisense oligodeoxynucleotide (ODN) complementary to the two HNRNPD binding sites in *circLARP1B* was synthesized to examine as an inhibitor of *circLARP1B*. The antisense ODN was modified with phosphorothioate (PS) and 2′‐*O*‐methyl (2′‐OMe),^[^ ^68^ ^]^ and termed ODN‐AS. ODN‐AS transfection in PLC cells increased the HNRNPD binding to *LKB1* 3′ UTR and upregulated LKB1 protein levels (Figure 6l,m). 5‐methylcytosine (m^5^C) modified sense single‐stranded RNA oligos (m^5^C‐ssRNA) containing the HNRNPD binding sites of *circLARP1B* were also tested.^[^ ^69^ ^]^ m^5^C‐ssRNA did not affect either the HNRNPD binding to *LKB1* 3′ UTR or LKB1 protein levels (Figure S7e,f, Supporting Information). These results indicated that the small m^5^C‐ssRNA oligos somehow could not function as a *circLARP1B* mimics in cells. On the other hand, the interaction between HNRNPD and *circLARP1B* could be blocked by ODN‐AS, and the potential effects of ODN‐AS in lipid metabolism, HCC metastasis, and even therapeutics could be further investigated.

### 2.7 *CircLARP1B* Deficiency in Mice Causes Liver Changes Attributable to Lkb1 Surplus

**Figure 7:**
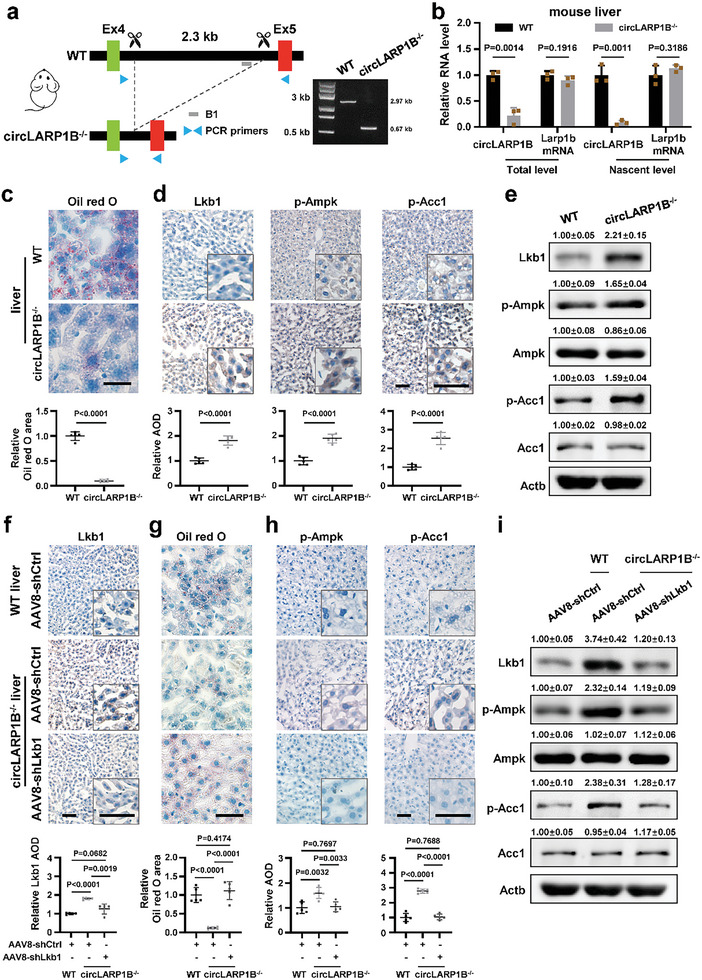
*CircLARP1B* deficiency in mice causes liver changes attributable to Lkb1 surplus. a) Strategy of knockout (KO) reverse‐complementary sequence in mouse *Larp1b* intron 4 using CRISPR‐Cas9. PCR products of mouse genotyping are shown. WT, wildtype; *circLARP1B* ^−/−^, KO of the intronic reverse‐complementary sequences; B1, mouse B1 repeat. b) RT‐qPCR analysis of the steady levels and nascent levels (with nuclear run‐on assay) of *circLARP1B* and *Larp1b* mRNA in WT and *circLARP1B* ^−/−^ mouse liver. Data are from three independent experiments. c) Representative Oil Red O staining and the quantification in WT and *circLARP1B* ^−/−^ mouse liver (*N* = 5 per group). Nuclei stained with hematoxylin (blue). Scale bar: 50 µm. d) Representative IHC staining and quantification of the proteins in liver from WT and *circLARP1B* ^−/−^ mice (*N* = 5 per group). Scale bar: 50 µm. AOD, average optical density. e) Western blot of the indicated proteins in livers from WT or *circLARP1B* ^−/−^ mice. f) Representative Lkb1 IHC staining in livers from WT or *circLARP1B* ^−/−^ mice with the intravenous tail injection of AAV8‐shCtrl or AAV8‐shLkb1 for three weeks (*N* = 5 per group). Scale bar: 50 µm. g) Representative Oil Red O staining in liver from WT or *circLARP1B* ^−/−^ mice with AAV8‐shCtrl or AAV8‐shLkb1 injection (*N* = 5 per group). shCtrl, negative control shRNA construct that gives rise to siRNA with scrambled sequences. Nuclei stained with hematoxylin (blue). Scale bar: 50 µm. h) Representative IHC staining of the indicated proteins in liver from WT or *circLARP1B* ^−/−^ mice with AAV8‐shCtrl or AAV8‐shLkb1 injection (*N* = 5 per group). Scale bar: 50 µm. i) Western blot of the indicated proteins in livers from WT or *circLARP1B* ^−/−^ mice with AAV8‐shCtrl or AAV8‐shLkb1 injection. Data are shown as mean ± SD. (c,g) Lipid level is defined as the percentage of Oil Red positive area calculated by Image‐Pro plus. (d,f,h) The IHC signal is defined as the average optical density (AOD) calculated by ImageJ. (e,i) The grayscale statistics of western blotting was performed by ImageJ. (c–i) All animals were kept in a controlled environment (23–25 °C with a 12‐h light‐dark cycle and lights on at 8:00 AM), on normal diet feeding with free access to water; all data were from mice at 9:00 AM. (b–d,f–h) *P*‐values by two‐tailed unpaired Student's *t*‐test.

To directly investigate the physiological roles of *circLARP1B* in animals, we used CRISPR/Cas9 to generate *circLARP1B* deficient mice, in which repeat sequences in intron 4 of murine *Larp1b* gene were deleted (*circLARP1B^−/−^*), with functional intronic elements such as the splice sites and pyrimidine tract remained unaltered (**Figure** **7**a). The mutant mice did not show statistically significant difference in body weight or litter size, when compared age‐matched WT mice (Figure S8a,b, Supporting Information). The *circLARP1B* expression was significantly decreased in livers of *circLARP1B^−/−^* mice compared to WT mice, while the total and nascent levels of *Larp1b* mRNA were unaltered (Figure 7b; Figure S8c, Supporting Information). The levels of *HNRNPD* mRNA and protein also showed no significant difference in livers of WT and *circLARP1B^−/−^* mice (Figure S8d, Supporting Information). Liver was the focus due to that it is the organ giving rise to HCC, and a central organ of metabolism in the body.

*CircLARP1B^−/−^* mice compared to WT mice had significantly reduced LDs in livers, examined by Oil red O staining (Figure 7c). Protein levels of Lkb1, p‐Ampk, and p‐Acc1 were significantly increased in *circLARP1B^−/−^* livers (Figure 7d,e; Figure S8e, Supporting Information). Further, we performed tail vein injection of 8‐week‐old *circLARP1B^−/−^* mice with hepatocyte‐directed adeno‐associated virus 8 (AAV8) to express shRNA against Lkb1 (AAV8‐shLkb1) or the corresponding control (AAV8‐shCtrl). When examined 3 weeks after the infection, AAV8‐shLkb1 resulted in effective Lkb1 knockdown in *circLARP1B^−/−^* livers, and Lkb1 depletion recovered the LDs levels in *circLARP1B^−/−^* livers to the levels in WT livers (Figure 7f,g). The increased p‐Ampk and p‐Acc1 levels in *circLARP1B^−/−^* liver were also restored nearly to the levels in WT liver (Figure 7h,i; Figure S8f, Supporting Information).

### 2.8 *CircLARP1B* Deficiency in Mice Impedes HCC Metastasis and Lipid Accumulation

**Figure 8:**
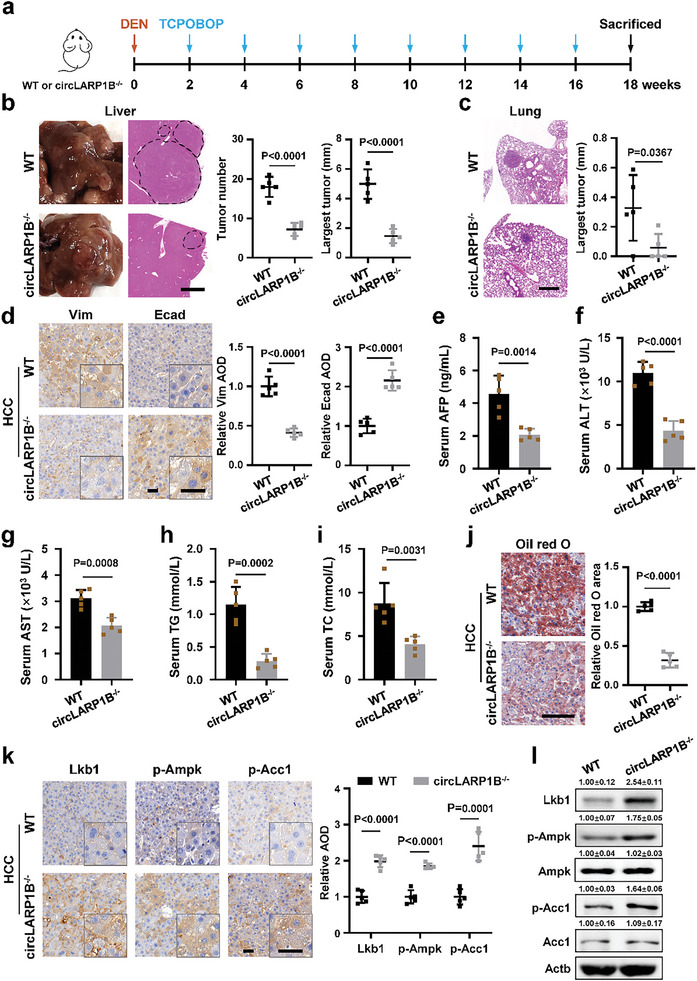
*CircLARP1B* deficiency impedes HCC metastasis and lipid accumulation in mice. a) Schematic illustration for generating the DEN‐induced mice HCC model. b) Representative images and H&E staining of liver tumor nodes from WT and *circLARP1B* ^−/−^ mice (*N* = 5 per group) after DEN‐induction for 18 weeks. Statistical analysis of liver tumor node numbers and diameters of the largest tumor node are shown. Scale bar: 2 mm. c) Representative H&E staining and the quantification of lung metastases in WT and *circLARP1B* ^−/−^ HCC mice (*N* = 5 per group). Scale bar: 400 µm. d) Representative Vim (Vimentin, a prometastasis marker) and Ecad (E‐cadherin, an antimetastasis marker) IHC staining of liver tumors from WT and *circLARP1B* ^−/−^ HCC mice (*N* = 5 per group). Scale bar: 50 µm. e–i) The serum AFP (e), ALT (f), AST (g), TG (h), and TC (i) levels in WT and *circLARP1B* ^−/−^ HCC mice (*N* = 5 per group). AFP, alpha‐fetoprotein (a biomarker for HCC); ALT, alanine aminotransferase; AST, aspartate aminotransferase; TG, triglyceride; TC, total cholesterol. j) Representative Oil Red O staining images and the quantification in liver tumors from WT and *circLARP1B* ^−/−^ HCC mice (*N* = 5 per group). Lipid level is defined as the percentage of Oil Red positive area calculated by Image‐Pro plus. Scale bar: 100 µm. k) Representative Lkb1, p‐Ampk, and p‐Acc1 IHC staining of liver tumors from WT and *circLARP1B* ^−/−^ HCC mice (*N* = 5 per group). Scale bar: 50 µm. l) Western blot of the indicated proteins of liver tumors from WT and *circLARP1B* ^−/−^ HCC mice. The grayscale statistics of western blotting was performed by ImageJ. (b–l) All animals were kept in a controlled environment (23–25 °C with a 12‐h light‐dark cycle and lights on at 8:00 AM), on normal diet feeding with free access to water, and fasted for 12 h before bleeding and sacrifice at 9:00 AM. All data are shown as mean ± SD. (d,k) The IHC signal is defined as the average optical density (AOD) calculated by ImageJ. (b–k) *P*‐values by two‐tailed unpaired Student's *t*‐test.

To examine physiological roles of *circLARP1B* in HCC at the whole organismal level, we induced HCC in mice with diethylnitrosamine (DEN), which is well established to cause severe liver damage and eventually hepatocarcinogenesis (**Figure** **8**a).^[^ ^70^ ^]^ After DEN induction for 10 weeks, liver inflammation examined by H&E staining was significantly lower in livers of *circLARP1B^−/−^* mice than that of WT mice (Figure S8g, Supporting Information). mRNA levels of *Il6* and *Tnf* (as inflammation indicators) were also decreased in livers of *circLARP1B^−/−^* mice (Figure S8h, Supporting Information). The presence of bridging fibrosis was also examined, and it was found that *circLARP1B^−/−^* mice had fewer Masson and Sirius Red signals in the portal areas (Figure S8i,j, Supporting Information). Consistently, mRNA levels of fibrosis‐related markers, *Col1a1*, *Tgfb1*, and *Mmp2* were decreased in livers of *circLARP1B^−/−^* mice (Figure S8k, Supporting Information). After DEN induction for 18 weeks (HCC mice), *circLARP1B^−/−^* mice exhibited fewer and smaller tumor nodes in livers, and significantly smaller lung metastatic nodes, compared with WT mice (Figure 8b,c). HNRNPD levels demonstrated no significant difference in liver tumors between WT and *circLARP1B^−/−^* HCC mice, while in HCC liver tumors of both genotypes, Hnrnpd levels were significantly higher than livers from age‐matched WT mice (Figure S8l,m, Supporting Information). Immunohistochemistry (IHC) of liver tumor nodes revealed that the prometastasis marker Vimentin (Vim) was lower and the antimetastasis marker E‐cadherin (Ecad) was higher in *circLARP1B^−/−^* HCC mice (Figure 8d). In consistence with HCC severity, the serum concentrations of alpha‐fetoprotein (AFP), a biomarker for HCC, was significantly lower in *circLARP1B^−/−^* HCC mice (Figure 8e). Alanine transaminase (ALT) and aspartate transaminase (AST), two markers of liver injury, were also significantly lower in the serum of *circLARP1B^−/−^* HCC mice (Figure 8f,g). *CircLARP1B^−/−^* HCC mice exhibited lower triglyceride (TG) and total cholesterol (TC) levels in serum, and significantly fewer LDs in liver tumor nodes (Figure 8h–j). Meanwhile, levels of Lkb1, p‐Ampk, and p‐Acc1 were significantly higher in liver tumor nodes from *circLARP1B^−/−^* HCC mice (Figure 8k,l; Figure S9a, Supporting Information).

**Figure 9:**
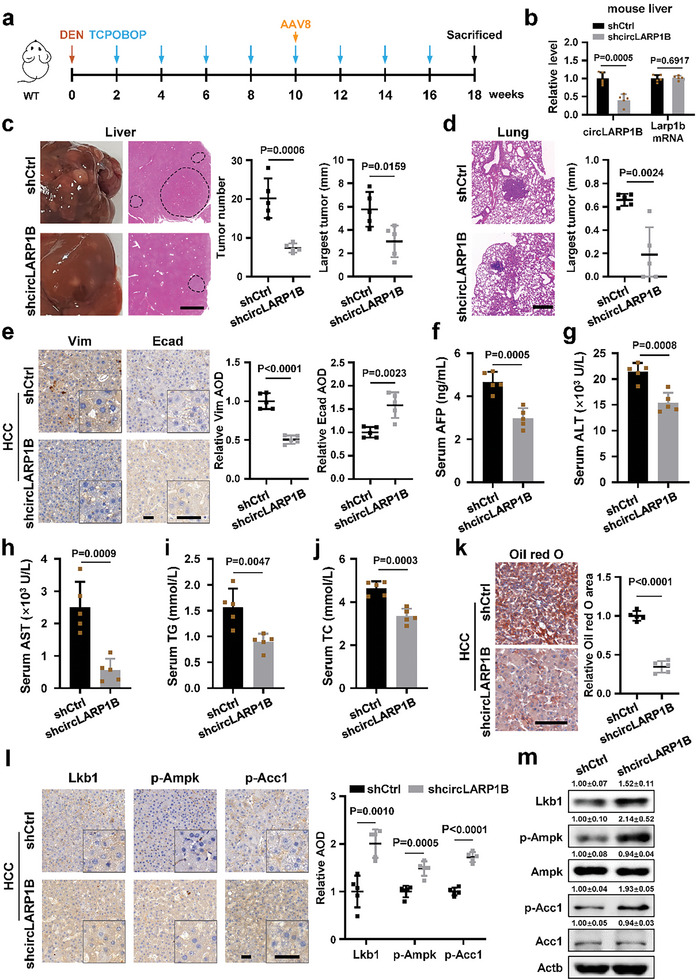
Hepatocyte‐directed *circLARP1B* knockdown impedes HCC metastasis and lipid accumulation in mice. a) Schematic illustration for generating the DEN‐induced mice HCC model. Hepatocyte‐directed AAV8 expressing shcircLARP1B and AAV8‐shCtrl were injected after induction for 10 weeks. b) RT‐qPCR analysis of *circLARP1B* and *Larp1b* mRNA levels of livers from WT mice (*N* = 5 per group) with AAV8‐shCtrl or AAV8‐shcircLARP1B injection. c) Representative images and H&E staining of liver nodes from WT mice (*N* = 5 per group) with AAV8‐shCtrl or AAV8‐shcircLARP1B injection after DEN‐induction for 18 weeks. Statistical analysis of liver tumor node numbers and diameters of the largest tumor node are shown. Scale bar: 2 mm. d) Representative H&E staining and the quantification of lung metastases in WT mice (*N* = 5 per group) with AAV8‐shCtrl or AAV8‐shcircLARP1B injection. Scale bar: 400 µm. e) Representative Vim (Vimentin, a prometastasis marker) and Ecad (E‐cadherin, an antimetastasis marker) IHC staining of liver tumors from WT mice (*N* = 5 per group) with AAV8‐shCtrl or AAV8‐shcircLARP1B injection. Scale bar: 50 µm. f–j) The serum AFP (f), ALT (g), AST (h), TG (i), and TC (j) levels in WT mice (*N* = 5 per group) with AAV8‐shCtrl or AAV8‐shcircLARP1B injection. k) Representative Oil Red O staining images and the quantification in liver tumors from WT mice (*N* = 5 per group) with AAV8‐shCtrl or AAV8‐shcircLARP1B injection. Lipid level is defined as the percentage of Oil Red positive area calculated by Image‐Pro plus. Scale bar: 100 µm. l) Representative Lkb1, p‐Ampk, and p‐Acc1 IHC staining of liver tumors from WT mice (*N* = 5 per group) with AAV8‐shCtrl or AAV8‐shcircLARP1B injection. Scale bar: 50 µm. m) Western blot of the indicated proteins of liver tumors from WT HCC mice with AAV8‐shCtrl or AAV8‐shcircLARP1B injection. The grayscale statistics of western blotting was performed by ImageJ. (b–m) All animals were kept in a controlled environment (23–25 °C with a 12‐h light‐dark cycle and lights on at 8:00 AM), on normal diet feeding with free access to water, and fasted for 12 h before bleeding and sacrifice at 9:00 AM. Data are shown as mean ± SD. (e,l) The IHC signal is defined as the average optical density (AOD) calculated by ImageJ. (b–l) *P*‐values by two‐tailed unpaired Student's *t*‐test.

To provide further insights for HCC metastatic roles of *circLARP1B* in liver cells, we infused hepatocyte‐directed AAV8 expressing shRNA against *circLARP1B* (AAV8‐shcircLARP1B) or the corresponding negative control (AAV8‐shCtrl) into WT mice after DEN induction for 10 weeks (**Figure** **9**a; Figure S3e, Supporting Information).^[^ ^71^ ^]^ In livers of WT mice infused with AAV8‐shcircLARP1B, effective knockdown of *circLARP1B* was observed, while the *Larp1b* mRNA was unaltered (Figure 9b). Consistent with results from *circLARP1B^−/−^* mice, AAV8‐shcircLARP1B injection resulted in fewer and smaller tumor nodes in livers, and smaller lung metastatic nodes, compared to AAV8‐shCtrl injection in WT mice (Figure 9c,d). The Vim IHC signals were lower, and the Ecad IHC signals were higher in liver tumors of WT mice with AAV8‐shcircLARP1B (Figure 9e). Serum AFP, ALT, AST, TG, and TC levels were also significantly lower in HCC mice with AAV8‐shcircLARP1B (Figure 9f–j). WT HCC mice infused with AAV8‐shcircLARP1B exhibited significantly fewer LDs and markedly higher levels of Lkb1, p‐Ampk, and p‐Acc1 in liver tumor nodes (Figure 9k–m; Figure S9b, Supporting Information). Even these results were based on AAV8‐mediated hepatocyte specific *circLARP1B* knockdown, in the real case of HCC, effects of the niche, immune cells, and other unaccounted parameters could still contribute to the overall effects of *circLARP1B* on metastasis.

Taken together, these results demonstrated that lower *circLARP1B* levels were unfavorable to HCC progression, lipid accumulation, and metastasis in mouse models, by affecting the same *circLARP1B–*HNRNPD–*LKB1*–AMPK regulatory pathway identified in HCC cell lines.

### 2.9 Lkb1 Is the Key Factor for the Effect of *CircLARP1B* in HCC Mouse Model

**Figure 10:**
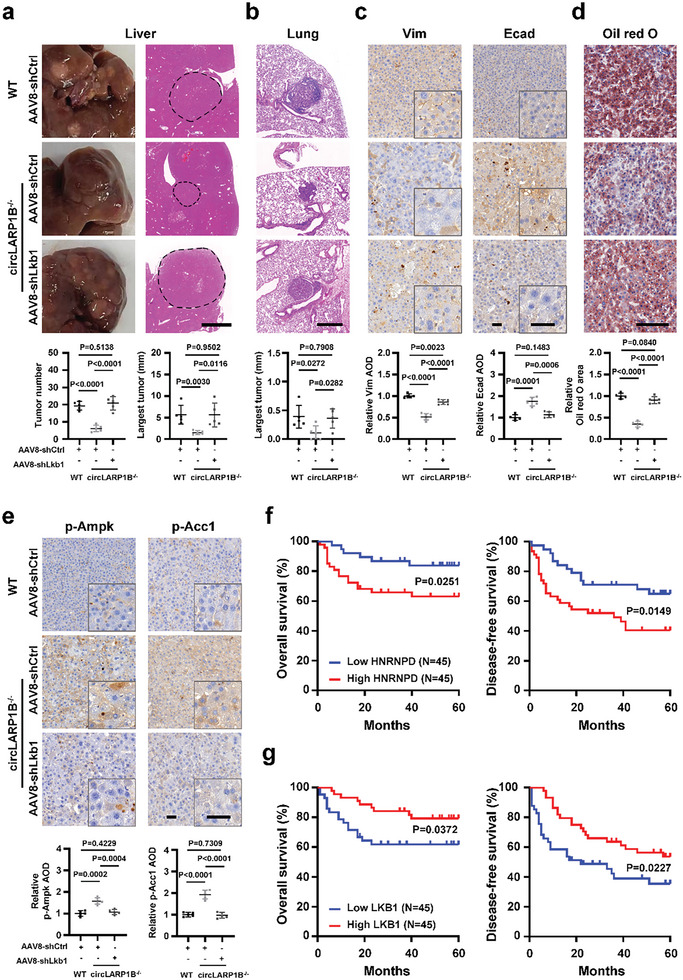
LKB1 is a key target for the roles of *circLARP1B* in HCC. a) Representative images and H&E staining of liver tumors from WT and *circLARP1B* ^−/−^ mice (*N* = 5 per group) with AAV8‐shCtrl or AAV8‐shLkb1 injection after DEN induction for 10 weeks. Statistical analysis of liver tumor numbers and diameter of the largest tumor is shown. Scale bar: 2 mm. b) Representative H&E staining and the quantification of lung metastases in WT and *circLARP1B* ^−/−^ HCC mice (*N* = 5 per group) with AAV8‐shCtrl or AAV8‐shLkb1 injection. Scale bar: 400 µm. c) Representative Vim (Vimentin, prometastasis marker) and Ecad (E‐cadherin, antimetastasis marker) IHC staining of liver tumors from WT and *circLARP1B* ^−/−^ mice (*N* = 5 per group) with AAV8‐shCtrl or AAV8‐shLkb1 injection. The quantification of IHC is shown. Scale bar: 50 µm. d) Representative Oil Red O staining of liver tumors from WT and *circLARP1B* ^−/−^ mice (*N* = 5 per group) with AAV8‐shCtrl or AAV8‐shLkb1 injection. Lipid level is defined as the percentage of Oil Red positive area calculated by Image‐Pro plus. Scale bar: 100 µm. e) Representative p‐Ampk and p‐Acc1 IHC staining of liver tumors from WT and *circLARP1B* ^−/−^ mice (*N* = 5 per group) with AAV8‐shCtrl or AAV8‐shLkb1 injection (top). The corresponding quantification is analyzed (bottom). Scale bar: 50 µm. f) Kaplan–Meier analysis of OS (left) and DFS (right) for 90 HCC patients collected. The red curve indicates survival in patients with higher HCC *HNRNPD* mRNA levels, and the blue line indicates survival in patients with lower HCC *HNRNPD* mRNA levels. g) Kaplan–Meier analysis of OS (left) and DFS (right) for 90 HCC patients with *LKB1* mRNA levels. (a–e) All animals were kept in a controlled environment (23–25 °C with a 12‐h light‐dark cycle and lights on at 8:00 AM), on normal diet feeding with free access to water, and fasted for 12 h before bleeding and sacrifice at 9:00 AM. Data are shown as mean ± SD. (c,e) The quantification of IHC as average optical density (AOD) calculated by ImageJ is shown. (a–e) *P*‐values by two‐tailed unpaired Student's *t*‐test. (f,g) *P*‐values are calculated by the log‐rank test.

We then performed tail vein injection with AAV8‐shLkb1 or the AAV8‐shCtrl once in *circLARP1B^−/−^* HCC mice also at the 10th week after DEN induction. Hepatic Lkb1 protein was successfully knocked down (Figure S9c, Supporting Information). We found that AAV8‐shLkb1 injection led to more and larger liver tumors, and larger lung metastatic nodes compared to AAV8‐shCtrl injection in *circLARP1B^−/−^* HCC mice (**Figure** **10**a,b). In *circLARP1B^−/−^* HCC mice upon AAV8‐shLkb1 application, liver tumor number and size, and lung metastases were as worse as what happened in the WT HCC mice (Figure 10a,b), indicating strongly that Lkb1 was the key downstream factor in hepatocytes for the effects of *circLARP1B*.

Lkb1 knockdown led to the increased Vim levels and the decreased Ecad levels in liver tumor nodes in *circLARP1B^−/−^* HCC mice (Figure 10c). Meanwhile, Lkb1 knockdown reversed these changes in Vim and Ecad levels caused by *circLARP1B* deficiency (Figure 10c). Lkb1 silencing nearly restored the circLARP1B‐deficient mediated suppressive effects on the levels of serum markers AFP, ALT, and AST in *circLARP1B^−/−^* HCC mice (Figure S9d–f, Supporting Information). Furthermore, Lkb1 depletion almost completely restored the inhibitory effects of *circLARP1B* deficiency on lipid accumulation in liver tumor nodes and serum TG and TC levels (Figure 10d; Figure S9g,h, Supporting Information). Increased levels of p‐Ampk and p‐Acc1 observed in the liver of *circLARP1B^−/−^* HCC mice were also eradicated upon Lkb1 knockdown (Figure 10e; Figure S9i,j, Supporting Information). Therefore, Lkb1 was the key factor in hepatocytes for the molecular regulatory pathway of *circLARP1B* in HCC lipid metabolism and metastasis.

### 2.10 Clinical Significance of HNRNPD, LKB1, and AMPK in HCC

Levels of *HNRNPD* mRNA were significantly higher in the 23 metastatic specimens compared to 67 nonmetastatic specimens we collected (Figure S10a, Supporting Information), and higher *HNRNPD* mRNA levels were positively associated with shorter OS and DFS analyzed based on these 90 HCC specimens (Figure 10f). HCC patients with higher *LKB1* mRNA levels in tumors had significantly longer OS and DFS (Figure 10g). On the other hand, levels of *LKB1* mRNA showed no statistically significant difference in metastatic versus nonmetastatic specimens (Figure S10b, Supporting Information). Presumably upstream mechanisms might upregulate both HNRNPD and *circLARP1B* in metastatic HCC (Figure 1g), and the two regulators had opposite effects on the levels of *LKB1* mRNA, with HNRNPD enhancing and *circLARP1B* destabilizing *LKB1* mRNA. Therefore, these regulations might lead to the balance of *LKB1* mRNA levels in metastatic versus nonmetastatic HCC. The *AMPK* mRNA levels demonstrated statistically significant increase in metastatic HCC, but its levels had no significant correlation to the OS and DFS of HCC patients (Figure S10c,d, Supporting Information). AMPK was reported to either promote or suppress HCC,^[^ ^11^, ^72^, ^73^, ^74^ ^]^ and in the *circLARP1B*–HNRNPD–*LKB1*–AMPK axis, the levels of p‐AMPK rather than the overall AMPK levels would be more relevant to HCC.

## 3 Discussion

Roles of circRNAs in human diseases are a field with extensive studies,^[^ ^5^, ^13^, ^14^, ^15^ ^]^ although further understandings of circRNAs with strong functions and in‐depth circRNA functional mechanisms are still required. In this study, a mammalian conserved circRNA *circLARP1B* is identified through the inspection of clinical specimens with different metastasis and prognostic features. Each *circLARP1B* molecule possesses two HNRNPD binding sites, and with more than 100 copies in the cytoplasm, *circLARP1B* can absolve a considerable portion of HNRNPD, and perturb the HNRNPD from binding to *LKB1* mRNA. *LKB1* mRNA is remarkably sensitive to low availability of HNRNPD, which leads to instability of this mRNA and then low LKB1 protein level. LKB1 as a kinase activates another mighty kinase AMPK that regulates downstream targets including the phosphorylation of ACC1 to constrain FAS and metastasis in HCC. Through this regulatory pathway, high levels of *circLARP1B* result in high level of FAS and metastasis in HCC (Figure S10e, Supporting Information).

CircRNAs are pervasively expressed and can be conserved across species,^[^ ^13^, ^14^, ^15^, ^33^, ^75^ ^]^ and several conserved circRNAs such as *CDR1as* in mammals and *circBoule* in metazoans have been investigated with animal models.^[^ ^19^, ^20^, ^24^ ^]^ As for HCC or even cancers, almost all reported circRNAs are either not conserved or not subjected to examinations with genetically manipulated animal models.^[^ ^5^, ^13^ ^]^ A previous report has revealed that the *p21* mRNA stability is enhanced by *circPCNX*, which inhibits HNRNPD's binding to *p21* mRNA in human HeLa cells,^[^ ^50^ ^]^ although *circPCNX* is not conserved in mice. Another circRNA, *circURI1* also functions as a protein sponge of HNRNPM to modulate alternative splicing of genes such as VEGF1 to inhibit gastric cancer metastasis,^[^ ^32^ ^]^ and *circURI1* is again not conserved in mice. The nature that *circLARP1B* is a mammalian conserved circRNA facilitates our study, especially enables detailed investigations of *circLARP1B* functions and functional mechanisms at the whole organismal level. It is evident that *circLARP1B* utilizes a conserved mechanism to play roles in regulating a cascade that dictates lipid synthesis in HCC, and maybe also in other physiological events or disorders.

Cancer metastasis is always coupled with lipid metabolism reprogramming.^[^ ^8^, ^9^, ^10^ ^]^ Take LDs as an example, besides other metastasis promoting functions, increased storage of lipids as LDs at least provide energy reservoir for metastatic cloning.^[^ ^7^, ^76^ ^]^ A recent study has demonstrated that ND‐654, as a liver‐specific ACC inhibitor, constrains hepatic de novo lipogenesis and HCC development by mimicking the effects of ACC phosphorylation.^[^ ^12^ ^]^ Besides the regulation of ACC activity in lipid biosynthesis, both LKB1 and AMPK are potent kinases, and LKB1/AMPK pathway has an array of known functionalities in HCC and cancers.^[^ ^11^, ^77^ ^]^ In addition to ACC1, the mTOR signaling has been known to promote FAS and is relevant to liver tumorigenesis,^[^ ^9^, ^44^ ^]^ which seems also regulated by *circLARP1B* through the AMPK pathway (Figure S2h, Supporting Information). mTOR has been subjected to extensive investigations, although the involvement of mTOR signaling in the effects of *circLARP1B* in HCC metastasis and lipid metabolism needs further exploration. Data from PLC cells demonstrate that FAS, but not FAO or lipophagy, is the process that regulated by *circLARP1B* (Figure 2d–j; Figure S2e–i, Supporting Information).

*CircLARP1B* deficiency leads to the dysregulation of a series of lipid classes (Figure 2f). PC, PE, PI, phosphatidylglycerol, and cardiolipin decreased in circLARP1B‐Def PLC cells belong to glycerophospholipids, which are associated with drug‐resistance in multiple cancers.^[^ ^78^ ^]^ For instance, colorectal cancer patients with abnormal biosynthesis of PC are resistant to oxaliplatin and 5‐fluorouracil.^[^ ^78^ ^]^ DG, TG, and monoglyceride that are also decreased in circLARP1B‐Def PLC cells belong to glycerolipids, which function as secondary messengers to activate downstream oncogenic signaling.^[^ ^79^ ^]^ Sphingomyelin (SM), *N*‐acetylhexosyl ceramide (CerG2GNAc1), ceramides phosphate, and phytosphingosine with increased levels in circLARP1B‐Def cells belong to sphingolipids, which are enriched in the lipid rafts localized in cellular membranes, and play critical roles in tumor metastasis, such as EMT and angiogenesis.^[^ ^80^ ^]^ It is highly possible that these molecules are not just substrates or products of lipid metabolism but also modulators that contribute to the effects of *circLARP1B* in HCC.

We have provided lines of evidence to reveal roles of *circLARP1B* in hepatocytes with cell lines, knockout mice, and hepatocyte‐directed *circLARP1B* knockdown mice, although the effects of the niche, immune cells, and other unaccounted parameters could also contribute to the final effects observed in HCC. For the effects of hepatocytic Lkb1 depletion in *circLARP1B^−/−^* HCC mice (Figure 10a–c), the restoration of liver tumor numbers and the Vim and Ecad IHC in liver tumors indicate that Lkb1 depletion in hepatocytes promotes malignant transformation; the restoration of liver tumor size indicates that Lkb1 depletion also promotes tumor growth. Hepatocyte‐directed AAV8‐mediated *circLARP1B* knockdown significantly inhibits HCC metastasis and relevant traits in mice, providing valuable insights for developing this circRNA as a therapeutic target of HCC.

We have provided experimental data from multiple levels to support that *LKB1* mRNA is the key downstream target of *circLARP1B* via perturbing HNRNPD in both human and mice. Even hepatocytic knockdown of *LKB1* seems sufficient to abolish effects of *circLARP1B* absence in HCC (Figure 10a–e; Figure S9c–j, Supporting Information), it is possible that the other mRNAs sensitive to *circLARP1B* and HNRNPD may also take part in the regulatory effects of *circLARP1B* to some degree. HNRNPD, a well‐recognized and conserved RBP with multiple post‐translational modifications, is upregulated in various human cancers including HCC, and is associated with poor prognosis and drug resistance.^[^ ^48^, ^49^, ^51^, ^81^, ^82^, ^83^, ^84^ ^]^ In the cytoplasm, HNRNPD can promote the decay of some mRNAs, and on the other hand stabilize some mRNA targets (e.g., *PTH* and *VHL*).^[^ ^48^, ^50^, ^56^ ^]^ Intriguingly, for *c‐MYC* mRNA, HNRNPD promotes its degradation in human lymphoblast cell line K562 and PLC liver cancer cells, and increases its stability in murine fibroblast 3T3 and human colorectal cancer cell line HCT116 indicating that the effect of HNRNPD may depend on the overall interactions among proteins bound to the mRNA.^[^ ^65^, ^85^ ^]^ We have demonstrated clinical significance of *circLARP1B*, HNRNPD, and *LKB1* in HCC. As for AMPK, it may play complex regulatory roles through various downstream signaling networks in cancers. In this study, p‐AMPK has been shown to play critical roles in regulating HCC metastasis through remodeling lipid metabolism, and further investigation concentrating on clinical relevance of the kinase role of AMPK in HCC is required.

Starting from screening with clinical specimens collected, we have identified the functional roles and mechanisms of the mammalian conserved *circLARP1B* in HCC metastasis and lipid metabolism. Data from both human and mice have revealed a pathway of *circLARP1B*–HNRNPD–*LKB1*–AMPK with potent regulatory effects in HCC lipid metabolism and metastasis. As it is closely associated with progression and prognosis, *circLARP1B* may be explored as a diagnostic marker or even a therapeutic target for HCC.

## 4 Experimental Section

### Clinical Specimens

All fresh HCC tumor tissues were collected from Sir Run Run Shaw Hospital, which was approved by the Human Research Ethics Committee of Sir Run Run Shaw Hospital (SRRSHLS2022Y0312). Written informed consent was obtained from each patient for this study. All samples were frozen and stored in liquid nitrogen after removal from the operation followed by washing with PBS twice.

### Mice

All mice were housed in an enriched environment under the standard conditions (23–25 °C temperature, 40–60% humidity) with a 12‐h light‐dark cycle (lights on from 08:00 to 20:00) at the Specific‐Pathogen‐Free facility with unrestricted access to food and water for the duration of the experiment, unless specified in the corresponding situation. All animal protocols were approved by the Animal Care and Use Committee of the University of Science and Technology of China (USTCACUC212201037). For genetically engineered mice, whole‐body *circLARP1B* ^−/−^ mice were generated with the CRISPR/Cas9 system as previously described.^[^ ^18^ ^]^ sgRNAs targeting *Larp1b* intron 4 were designed by CRISPick web‐server (https://portals.broadinstitute.org/gppx/crispick/public). The synthesized *Cas9* mRNA and sgRNAs were coinjected into fertilized embryos of C57BL/6J mice and 100 zygotes were implanted into five ICR strain surrogate mice. All F0 founders were genotyped at 2 weeks after birth with PCR amplification followed by Sanger sequencing. Specific primers for PCR were included in Table S5 of the Supporting Information. Positive F0 founders were backcrossed to C57BL/6 mice to obtain deficient allele heritable heterozygous F1 mice, which were further intercrossed to generate *circLARP1B* ^−/−^ homozygous mice. All *circLARP1B* ^−/−^ mice used for analyses were in parallel with age‐ and gender‐matched wildtype littermates (as the control group). Male mice were used for experiments in this study.^[^ ^86^, ^87^ ^]^

### Library Preparation for RNA‐seq

Total RNA from frozen tissues or cultured cells was extracted with the TRIzol reagent (Invitrogen) according to the manufacturer's protocols. Libraries were prepared with the TruSeq Ribo Profile Library Prep Kit (Illumina) according to the manufacturer's instructions and then subjected to sequencing in an Illumina Nova‐PE150 system (Novogene). Each library generated ≈100 million 150‐nt paired‐end read pairs.

### CircRNA Identification

The circRNA candidates were identified with CIRI2 and the BSJ reads per million were used to calculate circRNA levels. Briefly, the adapters were removed with Cutadapt (‐e 0.1 ‐O 5) to obtain clean reads. The reads continuously aligned to the human reference genome (hg19) were filtered with Bowtie (‐v 1) allowing one mismatch. The remaining reads were used to predict circRNA candidates with CIRI2 with default parameters. The differentially expressed circRNAs were determined by DEseq2 with a criterion (fold change >4 or <0.25, *P*‐value <0.05). For conservation analysis of circRNAs, the circRNA sequences were aligned to annotated murine circRNAs from the circAtlas database with Blast. Conserved circRNAs between human and mice were determined with a stringent cutoff (full‐length and BSJ sequences >75% identical).

### Cell Culture and Cell Transfection

The PLC/PRF/5 (PLC) cells were kindly provided by Dr. Yide Mei (USTC). HepG2, HEK293T, K1, and Hepa1‐6 cells all originated from the American Type Culture Collection. All above cells were maintained under standard conditions with the DMEM medium containing 10% FBS (CLARK, FB25015) and 1% penicillin/streptomycin (Beyotime, C0222) at 37 °C under 5% CO_2_. A PCR‐based method and DAPI staining were used to ensure cells without contamination of mycoplasma. All cell lines were authenticated by short‐tandem‐repeat profiling. Transfection of plasmids and siRNAs was conducted with Lipofectamine 2000 (Invitrogen) according to the manufacturer's protocol.

### CRISPR/Cas9‐Mediated CircLARP1B Deficient Cells

Two sgRNAs targeting the intron 4 of human LARP1B were designed based on http://crispr.mit.edu to knockout *circLARP1B*. After transfection of 2 µg pX330 plasmids expressing *Cas9* mRNA and targeted sgRNAs for two days, hundreds of mCherry‐positive single‐cell clones were sorted through the BD FACSAria III cell sorting system. Two weeks later, the colonies were split into two identical plates and one plate was harvested for PCR‐mediated genotype and confirmation by Sanger sequencing. The primer sequences are listed in Table S5 of the Supporting Information.

### OCR Measurements

The OCR analyses were performed using the Agilent Seahorse XF Cell Mito Stress Test Kit (Agilent, 103015‐100) as previously described.^[^ ^88^ ^]^ Briefly, 20 000 cells per well were seeded in a 96‐well XF cell culture microplate in the growth medium overnight at 37 °C in 5% CO_2_. OCR was measured with an XF96 analyzer in XF DMEM medium (Agilent, 103575‐100) containing 1 mm pyruvate, 2 mm glutamine, and 10 mm glucose, followed by sequential addition of 1.5 µm oligomycin (ATP synthase inhibitor), 1.0 µm FCCP (membrane potential uncoupler), 0.5 µm rotenone (Complex I inhibitor), and 0.5 µm antimycin A (Complex III inhibitor). Data were analyzed by the Seahorse XF Cell Mito Stress Test Report Generator package.

### FAO Rate Measurements

The FAO rate analyses were performed using the Agilent Seahorse XF Cell Mito Stress Test Kit (Agilent, 103015‐100) as previously described.^[^ ^89^ ^]^ Briefly, 15 000 cells per well were seeded in a 96‐well XF cell culture microplate in the growth medium overnight at 37 °C in 5% CO_2_. Then, cells were starved by incubating with substrate‐limited DMEM supplemented with 0.5 mm glucose, 1 mm glutamate, 0.5 mm carnitine, and 1% FBS. After 24‐h starvation, the medium was replaced by FAO assay medium (111 mm NaCl, 4.7 mm KCl, 1.25 mm CaCl_2_, 2 mm MgSO_4_, 1.2 mm NaH_2_PO_4_ supplemented with 2.5 mm glucose, 0.5 mm carnitine, and 5 mm HEPES, pH 7.4). Finally, BSA‐conjugated palmitate (Agilent, 102720‐100) was added to a final concentration of 50 mm, followed by sequential addition of 1.5 µm oligomycin, 1.0 µm FCCP, 0.5 µm rotenone, and 0.5 µm antimycin A. Data were analyzed by the Seahorse XF Cell Mito Stress Test Report Generator package.

### ^13^C‐Labeled Targeted Metabolic Flux Analysis

^13^C‐labeled targeted metabolic flux analysis was carried out as previously described with minor modifications.^[^ ^37^ ^]^ In brief, 10^7^ PLC cells were washed with 0.9% saline three times and incubated in substrate‐limited DMEM supplemented with 15  mm ^13^C‐glucose, 4  mm ^13^C‐glutamine, and 10% FBS for 24 h before metabolite extraction. After washing with cold 0.9% saline twice, cells were incubated with 500 µL cold extraction buffer (methanol:acetonitrile:water, 2:2:1, v/v/v). Harvested cells were sonicated for 2 min, centrifuged at 14 000 × *g* for 5 min at 4 °C and cell supernatants were transferred to new tubes. 400 µL chloroform was added and the tubes were vortexed, centrifuged at 14 000 × *g* for 5 min at 4 °C. The resulting phase separation resulted in an aqueous upper phase containing polar metabolites and an organic lower phase containing nonpolar metabolites. The aqueous upper phase was transferred to tubes and the lower chloroform phase was transferred to a glass tube. For reverse phase liquid chromatography separation, ACQUITY UPLC BEH C18 (100 × 2.1 mm, 1.7 µm, Waters) was used. Mass spectrometric data were acquired with a Q‐Exactive plus hybrid quadrupole–orbitrap mass spectrometer (Thermo) using negative ion electrospray ionization. The scan range was from 50 to 1000 *m*/*z*. The scan time for each function was set to 0.2 s. Ion monitoring conditions were defined as a capillary voltage of 2.0 kV, source temperature of 120 °C, and desolvation temperature of 500 °C. Data processing and ion annotation based on accurate mass were performed in TraceFinder 5.0 (Thermo) and Xcalibur 4.0 (Thermo). Metabolite mass isotopomer distribution was determined based on the ratio of the integrated peak areas of the chosen isotopomer to the sum of all the integrated peak areas of the possible isotopomers for the given metabolites.

### Plasmids Construction

All plasmids were constructed with restriction‐enzyme digestion and ligation or with recombinant methods (Vazyme, c113‐02). For *circLARP1B* overexpression in human, the circularized exons and the endogenous flanking sequences including one *Alu* pair were inserted into the pcDNA3 vector. The backbone vector of p3×FLAG‐Myc was used for constructing FLAG‐tagged HNRNPD and LKB1. The shRNA against the BSJ of human or murine *circLARP1B* was cloned into the vector pLKO.1 (Sigma) and the negative‐control shRNA (shCtrl, MFCD07785395) was obtained from the MISSION shRNA Library (Sigma). The sgRNAs for human or murine *circLARP1B* were inserted into the *Cas9‐* and sgRNA‐expressing backbone (pX330), which was engineered with expressing mCherry and a puromycin selectable marker. All plasmids have been sequenced for confirmation and further information about these plasmids is available upon request. Oligonucleotide sequences for primers used in plasmids construction, along with other oligo sequences used in probe preparation, siRNA, and biotin‐labeled nucleic acids were listed in Table S5 of the Supporting Information.

### Northern Blotting

Northern blotting was performed as previously described.^[^ ^90^ ^]^ The digoxigenin‐labeled RNA probe with antisense sequences to the *circLARP1B* BSJ was synthesized with a DIG Northern Starter Kit (Roche, 12039672910) according to the manufacturer's protocol. The primers for probe preparation were listed in Table S5 of the Supporting Information.

### Single‐Molecule Fluorescence In Situ Hybridization

The smFISH was carried out as previously described with minor modifications.^[^ ^91^ ^]^ Cells or tissues were fixed with 4% PFA for 10 min at room temperature and permeabilized with ice‐cold 70% ethanol overnight at −20 °C. For hybridization, cells or tissues were prehybridized at 37 °C for 30 min in hybridization buffer (30% formamide, 5× SSC, 9 mm citric acid, pH 6.0, 0.1% Tween‐20, 50 µg mL^−1^ heparin, 1× Denhardt's solution, and 10% dextran sulfate) after washing with 2× SSC twice. Then, samples were hybridized with 2 pmol probes overnight at 37 °C in the hybridization buffer. For the amplification stage, 18 pmol hairpins were heated at 95 °C for 90 s and cooled to room temperature in a dark drawer for 30 min. Samples were incubated with the denatured probes overnight at room temperature followed by three 5 min washes in 2× SSC buffer. After a 10 min incubation in DAPI (5 mg mL^−1^), the sections were washed three times for 5 min in 2× SSC buffer. The images were captured using the laser confocal microscopy LSM880 (Zeiss). The ImageJ was used to further process the data, and the ImageJ Plot Profile tool was applied to calculate signal intensity. All probe sequences were included in Table S5 of the Supporting Information.

### PCR Reactions

For RT‐PCR, 500 ng total RNA was reverse‐transcribed into complementary DNA (cDNA) with the GoScript Reverse Transcription System (Promega, A5001) according to the manufacturer's protocol. For PCR with genomic DNA (gDNA) as the template, gDNA was isolated by phenol/chloroform extraction. For semiquantitative RT‐PCR gels, 25–30 cycles were carried out. The RT‐qPCR was performed using GoTaq SYBR Green qPCR Master Mix (Promega, A6001) on a PikoReal 96 real‐time PCR system (Thermo). All PCR products were confirmed by Sanger sequencing. All used primer sequences are included in Table S5 of the Supporting Information.

### Nuclear/Cytosolic Fractionation

Cellular fractionation was carried out as previously described with minor modifications.^[^ ^18^ ^]^ Briefly, cells were incubated with hypotonic buffer (10 mm Tris‐HCl, pH 8.0, 140 mm NaCl, 1.5 mm MgCl_2_, 0.5% NP‐40, 1 mm DTT, and 0.1 U µL^−1^ RNase inhibitor (Promega, N2615)) on ice for 20 min after washing with PBS twice. After centrifugation at 1000 × *g* for 5 min at 4 °C, the supernatant was collected as the cytoplasmic fraction. The pellets were then resuspended in nuclear resuspension buffer (20 mm HEPES, pH 7.9, 400 mm NaCl, 1 mm EGTA, 1 mm EDTA, 1 mm DTT, and 0.1 U µL^−1^ RNase inhibitor followed by the incubation at 4 °C for 30 min. Nuclear fraction was collected after removing insoluble membrane debris by centrifugation at 12 000 × *g* for 15 min.

### Quantification of RNA Copy Number per Cell

The protocol was carried out as previously described.^[^ ^32^ ^]^ Total RNA was extracted from 10^6^ PLC and HepG2 cells, and cDNA was synthesized. The standard curve was used to calculate the copy numbers per cell in each cell line based on cell numbers and Ct values.

### Transwell Assay

Transwell invasion assay was performed using the chamber with Matrigel (BD Biosciences). Cells were seeded into the upper chamber cultured with the serum‐free DMEM medium, and DMEM containing 10% FBS was added to the lower chamber. After 24 h, the migrated cells on the outer membrane were stained with 0.1% crystal violet for 10 min. Images were captured with an inverted microscope (Olympus) and the number of invaded cells was quantified by ImageJ.

### Cell Viability and Number Measurement and Colony Formation

The cell viability was detected using MTT cell proliferation and Cytotoxicity Detection Kit (KeyGEN, KGA311). Cell numbers were measured by trypan blue assay using Trypan Blue Staining Cell Viability Assay Kit (Beyotime, C0011). For colony formation, cells were seeded into 6‐well plates (500 cells per well) and then were fixed and stained with 0.1% crystal violet for 10 min after two weeks.

### Nile Red Staining

To visualize lipid droplets, cells were washed with PBS twice, fixed with 4% PFA, and stained with 0.05 mg mL^−1^ Nile red solution for 10 min. The nuclei were further stained with Hoechst 33342, and images were captured using laser confocal microscopy LSM880 (Zeiss). For FASN inhibition, 50 nm IPI‐9119 (Selleck, E2667) was incubated with PLC cells for 72 h. The Nile red signal is defined as the mean gray value per cell (integrated density/area) quantified by ImageJ.

### Untargeted Metabolomics and Lipidomics

The untargeted metabolomics and lipidomics were carried out as previously described with minor modifications.^[^ ^92^ ^]^ All cells were maintained under standard conditions with DMEM medium containing 10% FBS for 48 h and then subjected to metabolomics analysis. 10^7^ cells were incubated with 800 µL cold methanol for protein removal and metabolite extraction after two PBS washes. The mixture was collected and centrifuged at 12 000 × *g* for 20 min, and the supernatant was further dried in a vacuum centrifuge. For LC‐MS analysis, samples were redissolved in 100 µL acetonitrile/water (1:1, v/v) solvent and centrifuged at 14 000 × *g* at 4 °C for 15 min. Then the supernatant was subjected to AB Triple TOF 6600 (AB SCIEX) and Q Exactive HF‐X (Thermo) for metabolomics and lipidomics, respectively (Shanghai Applied Protein Technology). For statistical analysis, the VIP value of each variable in the orthogonal partial least‐squares discriminant analysis model was calculated to indicate its contribution to the classification. The significantly differential metabolites were identified with the cutoff (VIP >1, *P*‐value <0.05). *P*‐values were generated by the two‐tailed Student's *t*‐test.

### Western Blotting

For western blots, samples were separated on SDS‐PAGE gels and then transferred to PVDF membranes (Millipore). Membranes were processed according to the ECL western blotting protocol (GE Healthcare). The grayscale statistics of western blotting were performed by ImageJ. The following antibodies were used in western blots: anti‐AMPK (CST, 5832), anti‐p‐AMPK (CST, 2535), anti‐ACC1 (CST, 3676), anti‐p‐ACC1 (CST, 3661), anti‐RAPTOR (CST, 2280), anti‐p‐RAPTOR (CST, 89146), anti‐VEGFA (Proteintech, 9003‐1‐AP), anti‐TGF‐β (Immunoway, YT4632), anti‐MFF (Proteintech, 17090‐1‐AP), anti‐p‐MFF (Immunoway, YP1403), anti‐p‐ULK1 (CST, 5869), anti‐ULK1 (CST, 8054), anti‐LKB1 (Immunoway, YT2573), anti‐HNRNPD (Thermo, PA5‐99469), anti‐AGO2 (Sigma, SAB4200085), anti‐eIF4G1 (CST, 8701), anti‐eIF4E (Abcam, ab33768), anti‐c‐MYC (CST, 9402), and anti‐ACTB (TransGen, HC201). Antibody validation is provided on the manufacturers’ websites.

### RNA Pull‐Down and RNA Immunoprecipitation

RNA pull‐down with 5′‐biotinylated antisense oligos and RIP were carried out as previously described with minor modifications.^[^ ^93^ ^]^ PLC cells and murine liver cells were cross‐linked (a total of 0.4 J cm^−2^) in a UV cross‐linker. Cells were harvested in ice‐cold lysis buffer (20 mm HEPES, pH 7.4, 10 mm KCl, 2 mm MgCl_2_, 0.5% NP‐40, 1 mm DTT, 1× Protease Inhibitor Cocktail, and 0.1 U µL^−1^ RNase inhibitor (Promega, N2615)) for 30 min on ice. The supernatant was collected after centrifugation at 1000 × *g* for 5 min at 4 °C, and subjected to sonication on ice for 5 min with a Sonics Vibra‐Cell (3 s on, 6 s off, 30%). 100 pmol biotinylated AS oligos (for pull‐down) or 2 µg antibody (for RIP) was added to the supernatant. After rotation 4 h at 4 °C, 50 µL M‐280 Streptavidin Dynabeads (Invitrogen, 11206D, for RNA pulldown) or Protein G Dynabeads (Invitrogen, 10004D, for RIP), which were blocked with 500 ng µL^−1^ yeast total RNA and 1 mg mL^−1^ BSA for 1 h at room temperature were added. After rotation 4 h at 4 °C, washing once with lysis buffer, twice with high salt lysis buffer (20 mm HEPES, pH 7.4, 10 mm KCl, 500 mm NaCl, 2 mm MgCl_2_, 0.5% NP‐40, 1 mm DTT, 1× Protease Inhibitor Cocktail, and 0.1 U µL^−1^ RNase inhibitor). The purified RNAs were analyzed by RT‐qPCR. The purified proteins were analyzed by MS after silver staining using Protein Stains K kit (Sangon Biotech, C500021‐0010) or western blot. The following antibodies were used: anti‐HNRNPD (Thermo, PA5‐99469); anti‐AGO2 (Sigma, SAB4200085). For HNRNPD RIP, *CCND1* and *Mef2c* mRNAs were used as positive controls in human and mice, respectively.^[^ ^50^, ^94^ ^]^ For AGO2 RIP, *TNFRSF12A* mRNA was used as a positive control.^[^ ^95^ ^]^

### Ribosome Profiling and Ribo‐seq Analysis

The ribosome profiling assay was conducted as previously described with minor modifications.^[^ ^32^ ^]^ The curve was generated with optical scanning at 254 nm using a Gradient Profiler (BioComp). *GAPDH* mRNA is a positive control, and *circHIPK3*, a negative control, is known to be noncoding.^[^ ^96^ ^]^ For Ribo‐seq analysis from previous studies (GSE147840, GSE125757, and GSE128320), the adapters were trimmed to obtain clean reads. The left reads with lengths ranging from 28 to 32 nt were then aligned to the human genome (hg19) with Bowtie (‐v 1) allowing one mismatch. Ribo‐seq signals shown in Figure S5b of the Supporting Information were presented by IGV visualization.

### Protein Mass Spectrometry

The specific silver‐stained band was cut, cleaned, and digested in gel with the digestion buffer (100 mm NH_4_HCO_3_, pH 8.5) containing trypsin (Promega, V5111). The samples were analyzed using an LC‐ESI‐MS/MS system after extraction and purification. Protech's ProtQuest software suite was used to search the mass spectrometric data against the UniProt protein database.

### ImmunofluorescenceStaining

PLC cells or HCC tissues were fixed with methanol/glacial acetic acid (3:1, v/v) at room temperature for 10 min after washing with PBS twice, and permeabilized with fresh PBS containing 1% Triton X‐100 on ice for 10 min. After blocking with 1% BSA for 30 min at room temperature, samples were incubated with the anti‐HNRNPD antibody (Thermo, PA5‐99469) or anti‐LKB1 antibody (Proteintech, 10746‐1‐AP) (1:200 dilution in 1% BSA) for 4 h at room temperature. With three 5 min washes in PBST buffer (PBS with 0.4% Tween‐20), samples were incubated with Goat Anti‐Rabbit Secondary Antibody Alexa Fluor 488 (Abcam, ab150077) (1:200 diluted in 1% BSA) for 2 h at room temperature, protected from light. After three 5 min washes in PBST buffer, nuclei were stained with DAPI, and the images were captured using laser confocal microscopy LSM880 (Zeiss).

### RIP‐seq and Data Analysis

RIP‐seq was carried out as previously described with minor modifications.^[^ ^18^, ^24^ ^]^ 5 × 10^7^ PLC cells were ultraviolet cross‐linked (a total of 0.4 J cm^−2^) in a UVP cross‐linker and harvested in ice‐cold lysis buffer (20 mm HEPES, pH 7.4, 10 mm KCl, 2 mm MgCl_2_, 0.5% NP‐40, 1 mm DTT, 1× Protease Inhibitor Cocktail, and 0.1 U µL^−1^ RNase inhibitor (Promega, N2615)) for 30 min on ice. The supernatant was collected after centrifugation at 1000 × *g* for 5 min at 4 °C, and subjected to sonication on ice for 5 min with a Sonics Vibra‐Cell (3 s on, 6 s off, 30%). The HNRNPD‐binding complexes were isolated by anti‐HNRNPD (Thermo, PA5‐99469) coupled with Protein G Dynabeads (Life Technology, 10004D). After one wash with lysis buffer, two washes with high salt lysis buffer (20 mm HEPES, pH 7.4, 10 mm KCl, 500 mm NaCl, 2 mm MgCl_2_, 0.5% NP‐40, 1 mm DTT, 1× Protease Inhibitor Cocktail, and 0.1 U µL^−1^ RNase inhibitor) and treatment of proteinase K, the mixture was subjected to RNA extraction with TRIzol reagent according to standard protocol. For RIP‐seq cDNA library preparation, the purified RNAs were ligated to adapters, reverse transcribed, PCR‐amplified for ≈25 cycles, and then subjected to high‐throughput sequencing using an Illumina Novoseq platform with a 150‐nt run length. For data analysis, the adapters were first trimmed to obtain clean reads, and the left reads were then mapped to the human genome (hg19) with Bowtie2. After the alignment, duplicates were removed and MACS2 was used for peak calling. The genome distribution of HNRNPD RIP‐seq signals was annotated according to the gene transfer format file from UCSC. For *circLARP1B*, all *circLARP1B* reads including the BSJ reads from the HNRNPD RIP‐seq were used for IGV visualization. For HNRNPD PAR‐CLIP reanalysis from previous studies (GSE52977), HNRNPD signals in the 3′ UTRs of targeted mRNAs were used for comparison.

### KEGG and GO Analysis

For KEGG pathway analysis, the metabolites were blasted against the online KEGG database (https://www.genome.jp/kegg/) to annotate the corresponding pathways. For GO analysis of HNRNPD binding targets regulated by *circLARP1B*, GOrilla web‐server (https://cbl‐gorilla.cs.technion.ac.il/) with default parameters was visualized by the ggplot2 package in R software.

### mRNA Stability Assay

For mRNA stability assay, cells were cultured with fresh DMEM at 37 °C for 12 h before being treated with 5 mg mL^−1^ Actinomycin D (Sigma, A4262). The samples were harvested at indicated times and then subjected to RT‐qPCR analysis.

### EIF4E and eIF4G1 RIP Assays

EIF4E and eIF4G1 RIP assays were performed as previously described with minor modifications.^[^ ^66^ ^]^ 5 × 10^7^ PLC cells were ultraviolet cross‐linked (a total of 0.4 J cm^−2^) in a UV cross‐linker, and harvested in ice‐cold RIPA buffer (50  mm Tris‐HCl, pH 8.0, 150  mm NaCl, 5  mm EDTA, 1% NP‐40, 0.1% SDS, 1 mm DTT, 1× Protease Inhibitor Cocktail) for 30 min on ice. The lysate was then subjected to sonication on ice for 5 min with a Sonics Vibra‐Cell (3 s on, 6 s off, 30%) and the supernatant was collected after centrifugation at 12 000 × *g* for 5 min at 4 °C. After the measurement of the absorbance at 260 nm, the supernatant was incubated with 3 U of RNase I (Invitrogen, AM2294) per A260 unit (Absorbance at 260 nm × volume in mL). An aliquot of the digestion reaction was saved as the input sample. The left supernatant was incubated with Protein G beads preconjugated antibody for the immunoprecipitation procedures. The bead–lysate mixture was rotated at room temperature (25 °C) for 30 min, and the input sample was placed alongside it. After collection on a magnetic rack at 4 °C, beads were rinsed briefly in 1 mL ice‐cold RIPA buffer, then collected and washed twice for 5 min each with rotating. Meanwhile, the bead supernatant and the input sample were aliquoted into two samples each (for RNA and protein isolation) and frozen. The RNA samples were subjected to TRIzol extraction. For further RT‐qPCR detection, primers used were included in Table S5 of the Supporting Information. The following antibodies were used: anti‐eIF4E (Abcam, ab33768); anti‐eIF4G1 (CST, 8701).

### Dual‐Luciferase Reporter Assay

The 3′ UTR sequence of *LKB1* mRNA was used as the 3′ UTR of firefly luciferase in the vector pGL3 (Promega, HG‐VQP0122). Vector expressing renilla luciferase was used as a loading control. After transfection in HEK293T cells for 24 h, relative luciferase activities were determined using a Dual‐Luciferase Reporter Assay System (Promega, E1910) according to the manufacturer's instructions.

### Immunofluorescence Combined with Fluorescence In Situ Hybridization

The IF combined with FISH assay was performed as previously described with several modifications.^[^ ^16^, ^60^ ^]^ PLC cells were fixed with methanol/glacial acetic acid (3:1, v/v) at room temperature for 10 min after washing with PBS twice and then permeabilized with freshly made PBS containing 0.01% Triton X‐100 (v/v) and 0.1 U µL^−1^ RNase inhibitor (Promega, N2615) on ice for 20 min. After blocking with 1% BSA for 30 min at room temperature, samples were incubated with the anti‐HNRNPD antibody (Thermo, PA5‐99469) (1:200 dilution in 1% BSA) for 3 h at room temperature. With three 5 min washes in PBST buffer (PBS with 0.4% Tween‐20), samples were incubated with Donkey anti‐Rabbit IgG Secondary Antibody Alexa Fluor 546 (Invitrogen, A10040) (1:200 diluted in 1% BSA) for 1 h at room temperature in dark. For *LKB1* mRNA FISH, probes were generated with Transcript Aid T7 High Yield Transcription Kit (Thermo, K0441), and then labeled with Alexa Fluor 488 by using the ULYSIS Nucleic Acid Labeling Kit (Invitrogen, 2161899) according to manufacturer's instructions. RNA probes were denatured at 80 °C for 5 min and placed on ice immediately. After washing with PBST buffer twice and 2× SSC once in dark, samples were incubated with RNA probes mixed with 20 ng µL^−1^ human Cot‐1 DNA (Invitrogen, 15279011), 500 ng µL^−1^ yeast total RNA (Invitrogen, AM7118) and 0.1 U µL^−1^ RNase inhibitor in 2× hybridization buffer (4× SSC, 40% dextran sulfate) at 37 °C for 15–17 h. Slides were washed with 2× SSC containing 0.1% Triton X‐100 at 45 °C. Nuclei were stained with DAPI and the images were captured using laser confocal microscopy LSM880 (Zeiss). The regain of interest (ROI) was drawn as a small rectangle and colocalization percentage of HNRNPD protein and *LKB1* mRNA in 15 ROIs was measured by using ImageJ plugin Coloc2.

### In Vitro RNA Circularization

Linear RNA fragment was generated with Transcript Aid T7 High Yield Transcription Kit (Thermo, K0441). In brief, 1 µg PCR‐amplified template, 2 µL T7 RNA polymerase enzyme, 0.5 mm NTPs, and 2 mm GMP were mixed and incubated for 4 h at 37 °C. After DNase I treatment, transcribed linear RNA was purified using phenol/chloroform (pH 4.5). For in vitro circularization, 50 µg linear RNA was incubated with T4 RNA ligase 1 (NEB, M0204) in 500 µL reaction buffer for overnight at 16 °C. After separation on 5% Urea PAGE gel, circularized RNA was cut in the corresponding band and eluted overnight in elution buffer (20 mm Tris‐HCl, pH 7.5, 250 mm NaOAc, 1 mm EDTA, and 0.25% SDS). The eluted RNA was then purified using phenol/chloroform (pH 4.5).

### In Vitro Binding/Competing Assay

In vitro synthesized *circLARP1B*, *ACTB* fragment, and *LKB1* 3′ UTR fragment RNAs were heated in RNA‐folding buffer (10 mm HEPES, pH 7.4 and 10 mm MgCl_2_) for 5 min at 65 °C and slowly cooled down over the course of 40 min to room temperature. For in vitro binding assay, equal amounts (10 pmol) of folded *circLARP1B* or *ACTB* RNAs were incubated with 1 µg purified Flag‐tagged HNRNPD protein (OriGene, TP320809) in 0.2 mL Binding buffer (50 mm HEPES, pH 7.4, 150 mm NaCl, 10 mm MgCl_2_, 1 mm DTT, 1× Protease Inhibitor Cocktail, and 0.1 U µL^−1^ RNase inhibitor) for 2 h at 4 °C. For in vitro competing assay, 5 pmol folded *circLARP1B* and 5 pmol *LKB1* 3′ UTR fragment RNA were incubated with 1 µg purified Flag‐tagged HNRNPD protein in 0.2 mL Binding buffer for 2 h at 4 °C. For both assays, one tenth of incubated solution was saved as input. The HNRNPD–RNA complex was purified with Protein G beads (Invitrogen, 10004D) preconjugated anti‐FLAG antibody (Sigma, F1804). After one Binding buffer wash and one high‐salt Binding buffer (50 mm HEPES, pH 7.4, 500 mm NaCl, 10 mm MgCl_2_, 1 mm DTT, 1× Protease Inhibitor Cocktail, and 0.1 U µL^−1^ RNase inhibitor) wash at 4 °C for 5 min, the interacted RNA was extracted with TRIzol according to the manufacturer's protocol for further analysis.

### DEN‐Induced HCC Mouse Model

Diethylnitrosamine (DEN)‐induced HCC mouse model was established as described previously.^[^ ^70^ ^]^ In brief, 2‐week‐old mice were injected with DEN (20 mg k^−1^g body weight, Sigma, N0258) to start the growth of tumors. The phenobarbital‐like inducer TCPOBOP (3 mg k^−1^g body weight; ApexBio, B8576) was injected every 2 weeks (a total of 8 times) to accelerate HCC progression after DEN injection for 2 weeks. All mice were kept in a controlled environment (23–25 °C with a 12‐h light‐dark cycle and lights on at 8:00 AM), on normal diet feeding with free access to water. For Figure S8g–k of the Supporting Information to examine liver inflammation and fibrosis, all data were collected from mice at 9:00 AM. For the other HCC mouse model, mice were fasted for 12 h before bleeding and sacrifice at 9:00 AM.

### Liver‐Directed AAV8‐Mediated CircLARP1B and Lkb1 Silencing

For preparing AAV8 to silence *circLARP1B* or Lkb1, shRNA was inserted into the p‐AAV‐MCS plasmid. AAV8‐shcircLARP1B, AAV8‐shLkb1 or AAV8‐shCtrl was generated with the AAV Helper‐Free System and infected mice with tail vein injection (10^12^ vg AAV8/mouse). For evaluating Lkb1's roles in normal livers as shown in Figure 7f–i and Figure S8f (Supporting Information), 8‐week‐old *circLARP1B^−/−^* or WT male mice were injected with AAV8‐shLkb1 or AAV8‐shCtrl, and sacrificed after 3 weeks. For AAV treatment in HCC mice models, AAV8 expressing shcircLARP1B, shLKB1 or shCtrl was injected once at 10 weeks after DEN injection and TCPOBOP was also injected into mice at the time point.

### Nuclear Run‐On Assay

For nuclear isolation, cells were harvested in ice‐cold solution I (10 mm Tris‐HCl, pH 7.5, 150 mm KCl, 4 mm Mg(OAc)_2_) and placed on ice for 10 min. After centrifugation at 1000 × *g* for 10 min, pellets were resuspended in solution II (10 mm Tris‐HCl, pH 7.5, 150 mm KCl, 4 mm Mg(OAc)_2_, 0.5% NP‐40, 10% glycerol, and 1 U µL^−1^ RNase inhibitor (Promega, N2615)) followed by intermittent shaking on ice for 20 min. The nuclear run‐on mixture (10 mm ATP, CTP, GTP, BrUTP, and the crude nuclei) was incubated at 30 °C for 5 min in the run‐on buffer (10 mm Tris‐HCl, pH 7.5, 5 mm MgCl_2_, 150 mm KCl, 1% Sarkosyl, and 2 mm DTT) in the presence of RNase inhibitor. The RNA was isolated with TRIzol reagent (Invitrogen) based on the manufacturer's instructions. Nascent transcripts were immunoprecipitated with anti‐BrdU antibody (Novus, NB500‐235) and converted to cDNA for use in RT‐qPCR assays.

### Oil Red O Staining

Frozen sections of liver tissues or tumor samples were washed with PBS twice followed by fixing with 4% PFA. After one ddH_2_O wash, samples were stained with freshly prepared 3 µg mL^−1^ Oil Red O for 1 h at room temperature. Nuclei were counterstained with hematoxylin. Images were captured using an inverted microscope (Olympus). The percentage of Oil Red O positive area is calculated by image‐Pro plus.

### Immunohistochemistry

For IHC staining, paraffin sections of murine tissues were deparaffinized with xylene and rehydrated with ethanol at decreasing concentrations. Then, samples were incubated with the indicated antibodies overnight at 4 °C followed by incubating with the correspondent secondary antibodies for 1 h at room temperature. Nuclei were stained with hematoxylin and images were captured using an inverted microscope (Olympus). The following antibodies were used in IHC: anti‐ACC1 (CST, 3676), anti‐p‐ACC1 (CST, 3661), anti‐AMPK (CST, 5832), anti‐p‐AMPK (CST, 2535), anti‐LKB1 (Immunoway, YT2573), anti‐Vim (Bioss, bs‐8533R), anti‐Ecad (Bioss, bs‐10009R), anti‐HNRNPD (Thermo, PA5‐99469). The average optical density (AOD) is calculated by ImageJ to indicate the IHC signal.

### H&E, Masson, and Sirius Red Staining

The lung or liver tissues were soaked in 10% formalin, dehydrated with graded alcohols, and then embedded in paraffin wax for sections. The paraffin sections were deparaffinized and rehydrated after being immersed in xylene. H&E Staining Kit (Abcam, ab245880) was used for H&E staining. The Fontana‐Masson Stain Kit (Abcam, ab150669) was used for Masson staining. The Picro Sirius Red Stain Kit (Abcam, ab150681) was used for Sirius Red staining. Images were captured using an inverted microscope (Olympus). The percentage of Masson and Sirius Red positive area is calculated by ImageJ.

### Measurement of Serum AFP, ALT, AST, TG, and TC

The serum AFP level was measured with Alpha‐fetoprotein ELISA Kit (NJJC, H226‐1‐1). The ALT and AST levels in serum were detected with Micro Glutamic‐pyruvic Transaminase Assay Kit (Solarbio, BC1555) and Micro Glutamic‐oxalacetic Transaminase Assay Kit (Solarbio, BC1565), respectively. The serum TG and TC levels were examined by Triglycerides Assay Kit (NJJC, F001‐1‐1) and Total Cholesterol Assay Kit (NJJC, F002‐1‐1), respectively.

### Image Processing

All images of the Nile Red staining, smFISH, and IF were taken by confocal microscope LSM880 (Zeiss). All images of the Transwell assay, Oil red O staining, and IHC were taken with an inverted fluorescence microscope IX73 (Olympus). Image processing and quantification were performed by ImageJ or Image‐Pro plus (as stated specifically in the corresponding method).

### Statistical Analysis

Statistical analysis was carried out using GraphPad Prism version 8.0. Either Student's *t*‐tests, two‐way ANOVA tests were used to calculate *P*‐values, as indicated in the figure legends. For Student's *t*‐tests, the values reported in the graphs represent averages with error bars showing S.D. After analysis of variance with F tests, the statistical significance and *P*‐values were evaluated with Student's *t*‐tests. Statistical significance was defined as *P*‐value < 0.05. The detailed statistical analysis applied to each experiment is included in the figure legends. The sample size (*N*) was represented in the corresponding figure legends.

## Conflict of Interest

J.L., X.W., and G.S. have an ownership interest in a patent related to this research.

## Author Contributions

J.L. and X.W. contributed equally to this work. G.S. conceived of and designed this project. J.L., X.W., and S.C. performed experiments. J.L. and X.W. performed bioinformatics analyses of RNA‐seq data, circRNA profiling, and RIP‐seq. X.C., L.S., B.L., and Z.S. provided clinical specimens. X.W., X.C., and G.S. analyzed the results. J.L., X.W., and G.S., wrote the manuscript. All authors have discussed the results and made comments on the manuscript. All authors approved the final manuscript.

## Supporting information

Supporting Information

Supplemental Table 1

Supplemental Table 2

Supplemental Table 3

Supplemental Table 4

Supplemental Table 5

## Data Availability

All high‐throughput sequencing data in this study have been deposited to the Gene Expression Omnibus (GEO) database and are available under Accession No. GSE217403. All metabolomics, lipidomics, and metabolic flux data in this study are available in Metabolomics Workbench with the sproject (DOI: 10.21228/M8CM5D). All experimental materials generated in this study are available upon request.
