# Supplementary material for: A Mammalian Conserved Circular RNA CircLARP1B Regulates Hepatocellular Carcinoma Metastasis and Lipid Metabolism
Source: Adv Sci (Weinh). 2023 Nov 12;11(2):2305902. doi: 10.1002/advs.202305902 (PMC10787103; doi:10.1002/advs.202305902)
Supplement: Supplementary file 1 — Supporting Information [file ADVS-11-2305902-s006.pdf]

ADVANCED SCIENCE
Open Access

Supporting Information

for *Adv. Sci.*, DOI 10.1002/advs.202305902

A Mammalian Conserved Circular RNA *CircLARP1B* Regulates Hepatocellular Carcinoma Metastasis and Lipid Metabolism

Jingxin Li, Xiaolin Wang, Liang Shi, Boqiang Liu, Zhiyong Sheng, Shuhui Chang, Xiujun Cai* and Ge Shan**

Supporting Information

A Mammalian Conserved Circular RNA CircLARP1B Regulates Hepatocellular Carcinoma Metastasis and Lipid Metabolism

Jingxin Li, Xiaolin Wang[*]*, Liang Shi, Boqiang Liu, Zhiyong Sheng, Shuhui Chang, Xiujun Cai*[*]*, Ge Shan*[*]

Figure S1

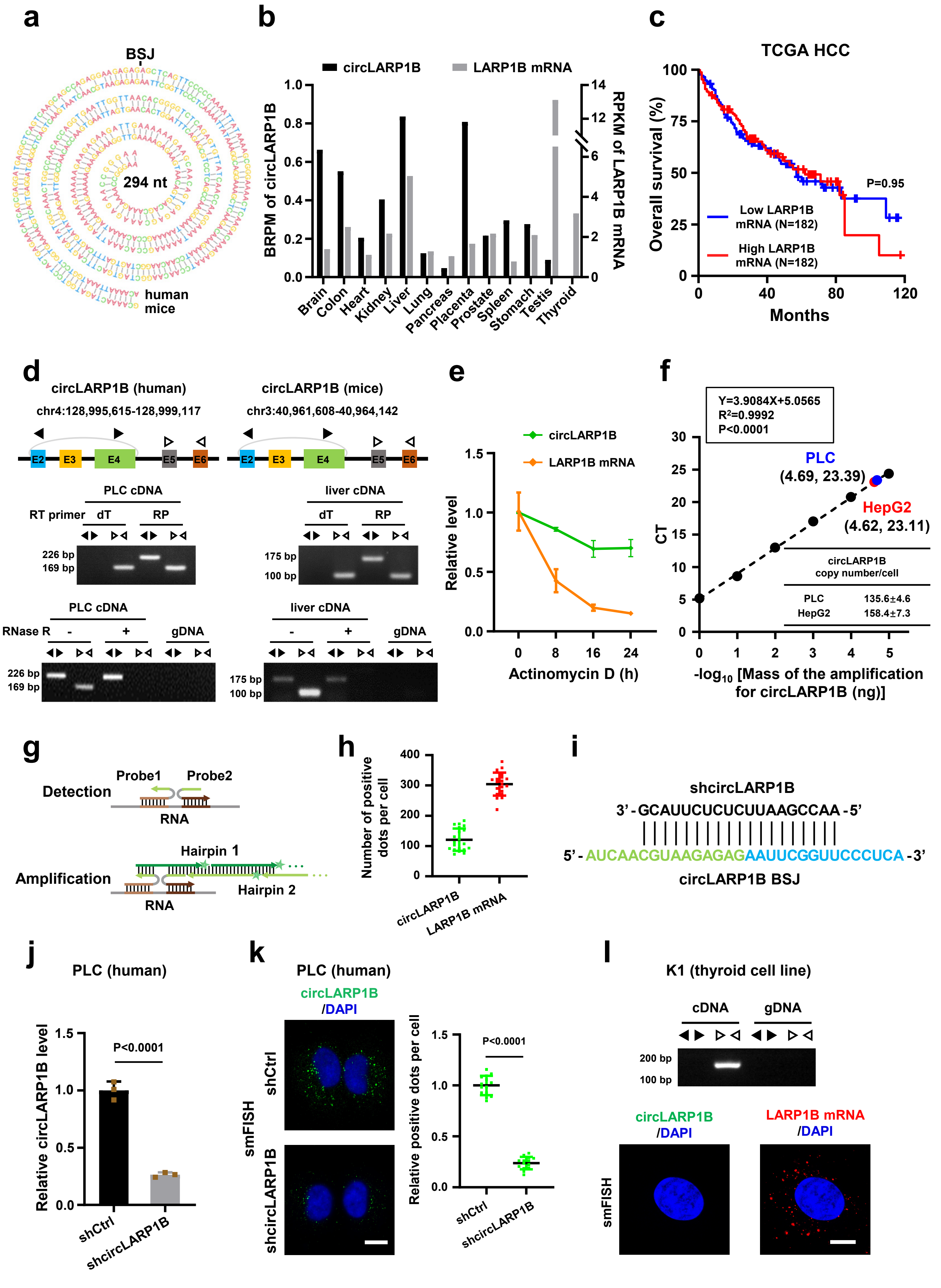

a
BSJ
294 nt
human
mice
b
circLARP1B
LARP1B mRNA
BRPM of circLARP1B
RPKM of LARP1B mRNA
Brain
Colon
Heart
Kidney
Liver
Lung
Pancreas
Placenta
Prostate
Spleen
Stomach
Testis
Thyroid
c
TCGA HCC
Overall survival (%)
P=0.95
Low LARP1B mRNA (N=182)
High LARP1B mRNA (N=182)
Months
d
circLARP1B (human)
chr4:128,995,615-128,999,117
circLARP1B (mice)
chr3:40,961,608-40,964,142
E2
E3
E4
E5
E6
PLC cDNA
RT primer
dT
RP
226 bp
169 bp
liver cDNA
175 bp
100 bp
RNase R
gDNA
e
circLARP1B
LARP1B mRNA
Relative level
Actinomycin D (h)
f
Y=3.9084X+5.0565
R²=0.9992
P<0.0001
CT
PLC
(4.69, 23.39)
HepG2
(4.62, 23.11)
circLARP1B copy number/cell
PLC 135.6±4.6
HepG2 158.4±7.3
-log10 [Mass of the amplification for circLARP1B (ng)]
g
Detection
Probe1
Probe2
RNA
Amplification
Hairpin 1
Hairpin 2
RNA
h
Number of positive dots per cell
circLARP1B
LARP1B mRNA
i
shcircLARP1B
3'-GCAUUCUCUCUUAAGCCAA-5'
5'-AUCAACGUAAGAGAGAAUUCGGUUCCCUCA-3'
circLARP1B BSJ
j
PLC (human)
Relative circLARP1B level
P<0.0001
shCtrl
shcircLARP1B
k
PLC (human)
circLARP1B /DAPI
smFISH
shCtrl
shcircLARP1B
Relative positive dots per cell
P<0.0001
l
K1 (thyroid cell line)
cDNA
gDNA
200 bp
100 bp
circLARP1B /DAPI
LARP1B mRNA /DAPI
smFISH

Figure S1. Identification of *circLARP1B* in human and mice. a) Alignment of human and murine *circLARP1B*. BSJ, back-splicing junction. b) The expression of *circLARP1B* and *LARP1B* mRNA in human tissues based on analyses of circAtlas and NCBI databases. BRPM, BSJ reads per million. RPKM, reads per kilobase per million. c) Kaplan–Meier analysis of overall survival for HCC patients based on the TCGA database. The red curve represents survival in HCC patients with higher *LARP1B* mRNA levels, and the blue line indicates survival in HCC patients with lower *LARP1B* mRNA levels. d) Characterization of *circLARP1B* in human (hg19) and mice (mm10). *CircLARP1B* and *LARP1B* mRNA are amplified with the indicated divergent (to amplify circRNA) and convergent primers, respectively. gDNA, genomic DNA. RT, Reverse Transcription. dT, oligo d(T). RP, random primer. e) Stability assay of *circLARP1B* and *LARP1B* mRNA in human PLC cells. f) Quantification of *circLARP1B* copy numbers in two human HCC cell lines (HepG2 and PLC). The Ct values and the amount of purified DNA fragments corresponding to *circLARP1B* amplicon are plotted to generate a standard curve by RT-qPCR. R represents Spearman's correlation coefficient, and the *P* value is calculated by Spearman's correlation test. The red and blue dots present the Ct values and the amount of *circLARP1B* from the cDNA used in HepG2 and PLC cells, respectively. The inset presents the calculated copy numbers of *circLARP1B* in HepG2 and PLC cells. g) Schematic procedure for smFISH. More experimental details are available in the Experimental Section. h) Quantification of positive dots of *circLARP1B* and *LARP1B* mRNA smFISH in PLC cells shown in Figure 1c. N=20. The numbers of smFISH dots are almost equivalent to RNA copies per cell. i) The siRNA sequences produced from the shRNA targeting the *circLARP1B* BSJ. j) RT-qPCR of *circLARP1B* in PLC cells treated with shcircLARP1B or shCtrl; data are from three independent experiments. shCtrl, shRNA that generates siRNA with scrambled sequences. k) Representative smFISH of *circLARP1B* (green) in PLC cells treated with shcircLARP1B or shCtrl. Quantification of smFISH signals is shown (right). N=15. Scale bar, 10 μm. l) Semi-quantitative RT-PCR gels of *circLARP1B* and *LARP1B* mRNA in K1 cells (a thyroid cell line) (top).

Representative smFISH images of *circLARP1B* (green) and *LARP1B* mRNA (red) in K1 cells (bottom). Blue, DAPI staining of nuclei. Scale bar, 10 μm. For (e), (h), (j) and (k), data are shown as mean ± SD. For (c), *P* value by the log-rank test. For (j) and (k), *P* values by two-tailed unpaired Student's *t*-test.

Figure S2

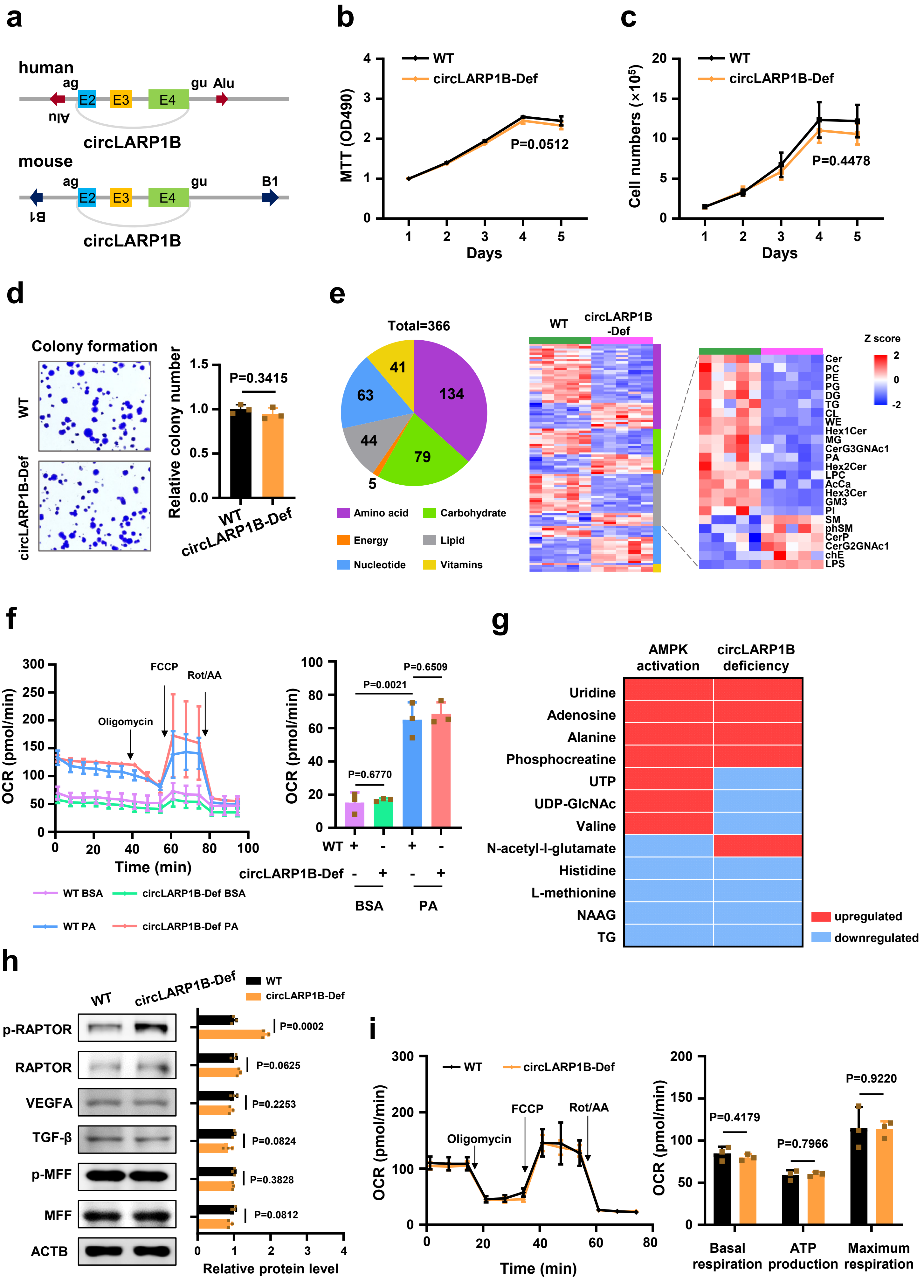
a
human
ag
E2
E3
E4
gu
Alu
Alu
circLARP1B
mouse
ag
gu
B1
B1
circLARP1B
b
WT
circLARP1B-Def
MTT (OD490)
P=0.0512
Days
c
WT
circLARP1B-Def
Cell numbers (×10⁵)
P=0.4478
Days
d
Colony formation
WT
circLARP1B-Def
Relative colony number
P=0.3415
WT
circLARP1B-Def
e
Total=366
134
79
44
63
41
5
Amino acid
Carbohydrate
Energy
Lipid
Nucleotide
Vitamins
WT
circLARP1B
-Def
Z score
Cer
PC
PE
PG
DG
TG
CL
WE
Hex1Cer
MG
CerG3GNAc1
PA
Hex2Cer
LPC
AcCa
Hex3Cer
GM3
PI
SM
phSM
CerP
CerG2GNAc1
chE
LPS
f
OCR (pmol/min)
Oligomycin
FCCP
Rot/AA
Time (min)
WT BSA
circLARP1B-Def BSA
WT PA
circLARP1B-Def PA
P=0.6770
P=0.0021
P=0.6509
WT
circLARP1B-Def
BSA
PA
g
AMPK activation
circLARP1B deficiency
Uridine
Adenosine
Alanine
Phosphocreatine
UTP
UDP-GlcNAc
Valine
N-acetyl-l-glutamate
Histidine
L-methionine
NAAG
TG
upregulated
downregulated
h
WT
circLARP1B-Def
p-RAPTOR
RAPTOR
VEGFA
TGF-β
p-MFF
MFF
ACTB
P=0.0002
P=0.0625
P=0.2253
P=0.0824
P=0.3828
P=0.0812
Relative protein level
i
WT
circLARP1B-Def
OCR (pmol/min)
Oligomycin
FCCP
Rot/AA
Time (min)
P=0.4179
P=0.7966
P=0.9220
Basal respiration
ATP production
Maximum respiration

Figure S2. Involvement of *circLARP1B* in cell viability, colony formation and downstream pathways regulated by AMPK. a) Alignment of the genomic loci of human *circLARP1B* and murine *circLARP1B*. 5' splicing site (AG), 3' splicing site (GU), exon (E); *Alu* and *B1* are intronic repeat elements in human and mice, respectively. b, c) Cell survival measured by MTT assay (b) and cell numbers measured by trypan blue assay (c) in WT and circLARP1B-Def PLC cells. d) Colony formation assays and the quantification with WT and circLARP1B-Def PLC cells. e) Distribution of polar metabolites detected by untargeted metabolomics in WT and circLARP1B-Def PLC cells (left). Heatmap of the 105 significantly changed metabolites (including lipid classes) (VIP >1, P value <0.05) in metabolomics of WT and circLARP1B-Def PLC cells (right). VIP, variable importance in the projection. f) Oxygen consumption rate (OCR) measured by Sea horse XF assays in WT and circLARP1B-Def PLC cells with or without addition of exogenous fatty acids (PA). Basal OCR levels during the first 40 min are shown (right). PA, palmitate. g) Heatmap of changes of 12 metabolites under AMPK activation or *circLARP1B* deficiency. These 12 ones are known to be regulated by AMPK activation in HCC.[39-41] Red, upregulated; blue, downregulated. h) Western blots and the quantification of p-RAPTOR, RAPTOR, VEGFA, TGF-β, p-MFF, and MFF proteins in WT and circLARP1B-Def PLC cells. ACTB, Actin b protein used as a loading control. The grey-scale statistics of western blotting was performed by Image J. i) Mitochondrial respiration profile of WT and circLARP1B-Def PLC cells. Oxygen consumption rate (OCR) was measured by Sea horse XF assays at three-time points. Basal respiration, ATP production and maximum respiration are shown (right). For (b-d), (f), (h) and (i), data are shown as mean ± SD from three independent experiments. For (b) and (c), P values by two-way ANOVA. For (d), (f), (h) and (i), P values by two-tailed unpaired Student's t-test.

Figure S3

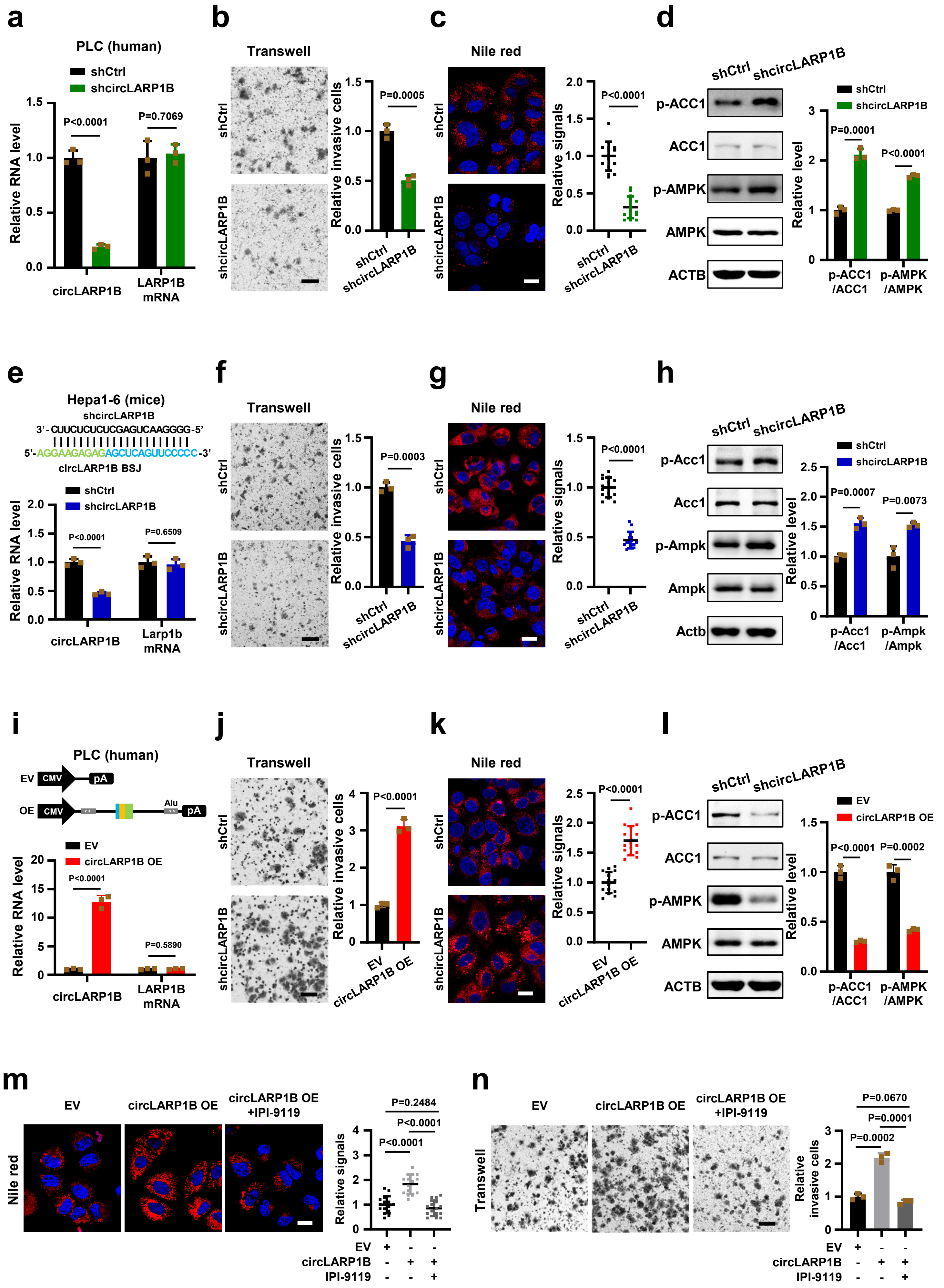
a
PLC (human)
shCtrl
shcircLARP1B
Relative RNA level
P<0.0001
P=0.7069
circLARP1B
LARP1B mRNA
b
Transwell
shCtrl
shcircLARP1B
Relative invasive cells
P=0.0005
c
Nile red
Relative signals
P<0.0001
d
p-ACC1
ACC1
p-AMPK
AMPK
ACTB
Relative level
P=0.0001
P<0.0001
p-ACC1 /ACC1
p-AMPK /AMPK
e
Hepa1-6 (mice)
shcircLARP1B
3'-CUUCUCUCUCGAGUCAAGGGG-5'
5'-AGGAAGAGAGAGCUCAGUUCCCCC-3'
circLARP1B BSJ
P<0.0001
P=0.6509
Larp1b mRNA
f
P=0.0003
g
P<0.0001
h
p-Acc1
Acc1
p-Ampk
Ampk
Actb
P=0.0007
P=0.0073
p-Acc1 /Acc1
p-Ampk /Ampk
i
EV
OE
CMV
pA
Alu
circLARP1B OE
P<0.0001
P=0.5890
j
k
l
P<0.0001
P=0.0002
m
EV
circLARP1B OE
circLARP1B OE +IPI-9119
Nile red
P=0.2484
P<0.0001
circLARP1B
IPI-9119
n
Transwell
P=0.0670
P=0.0001
P=0.0002

Figure S3. *CircLARP1B* regulates cell invasion and lipid accumulation in mammalian cells. a) RT-qPCR of *circLARP1B* and *LARP1B* mRNA in PLC cells treated with shcircLARP1B or shCtrl. shCtrl, shRNA control gives rise to siRNA with scrambled sequences; shcircLARP1B, shRNA against the human *circLARP1B* BSJ. b) Transwell assays of PLC cells treated with shcircLARP1B or shCtrl. Scale bar, 100 μm. c) Representative Nile red staining of PLC cells treated with shcircLARP1B or shCtrl. N=15. Scale bars, 20 μm. d) Western blot images and quantification of the indicated proteins in PLC cells treated with shcircLARP1B or shCtrl. e) RT-qPCR of *circLARP1B* and *Larp1b* mRNA in Hepa1-6 cells treated with shcircLARP1B or shCtrl. shCtrl, shRNA control that gives rise to siRNA with scrambled sequences; shcircLARP1B, shRNA against the murine *circLARP1B* BSJ. f) Transwell assays and corresponding quantification of Hepa1-6 cells treated with shcircLARP1B or shCtrl. Scale bar, 100 μm. g) Representative Nile red staining and the quantification of Hepa1-6 cells treated with shcircLARP1B or shCtrl. N=15. Scale bar, 20 μm. h) Western blot images and quantification of the indicated proteins in Hepa1-6 cells treated with shcircLARP1B or shCtrl. i) RT-qPCR analysis of *circLARP1B* and *LARP1B* mRNA upon *circLARP1B* overexpression (OE). EV, empty vector control. Construction for *circLARP1B* overexpression with the circularized sequence along with its flanking *Alu* pairs is illustrated. j) Transwell assays of PLC cells after *circLARP1B* overexpression. Scale bars, 100 μm. k) Representative Nile red staining of PLC cells after *circLARP1B* overexpression. N=15, Scale bars, 20 μm. l) Western blot and the quantification of the indicated proteins in PLC cells upon *circLARP1B* overexpression. m) Representative Nile red (red) and Hoechst 33342 (nuclei, blue) staining of PLC cells treated with IPI-9119 (a FASN inhibitor) under *circLARP1B* overexpression or not. N=15. Scale bar, 20 μm. n) Transwell assays of PLC cells treated with IPI-9119 under *circLARP1B* overexpression or not. Scale bar, 100 μm. For (c), (g), (k) and (m), the Nile red signal is defined as the mean gray value per cell quantified by Image J. For (d), (h) and (l), the grey-scale statistics of western blotting was performed by Image J. For (a), (b), (d-f), (h-j), (l) and (n), data are from three independent experiments. For (a-n), data are shown as mean ± SD; *P* values by two-tailed unpaired Student's *t*-test.

Figure S4

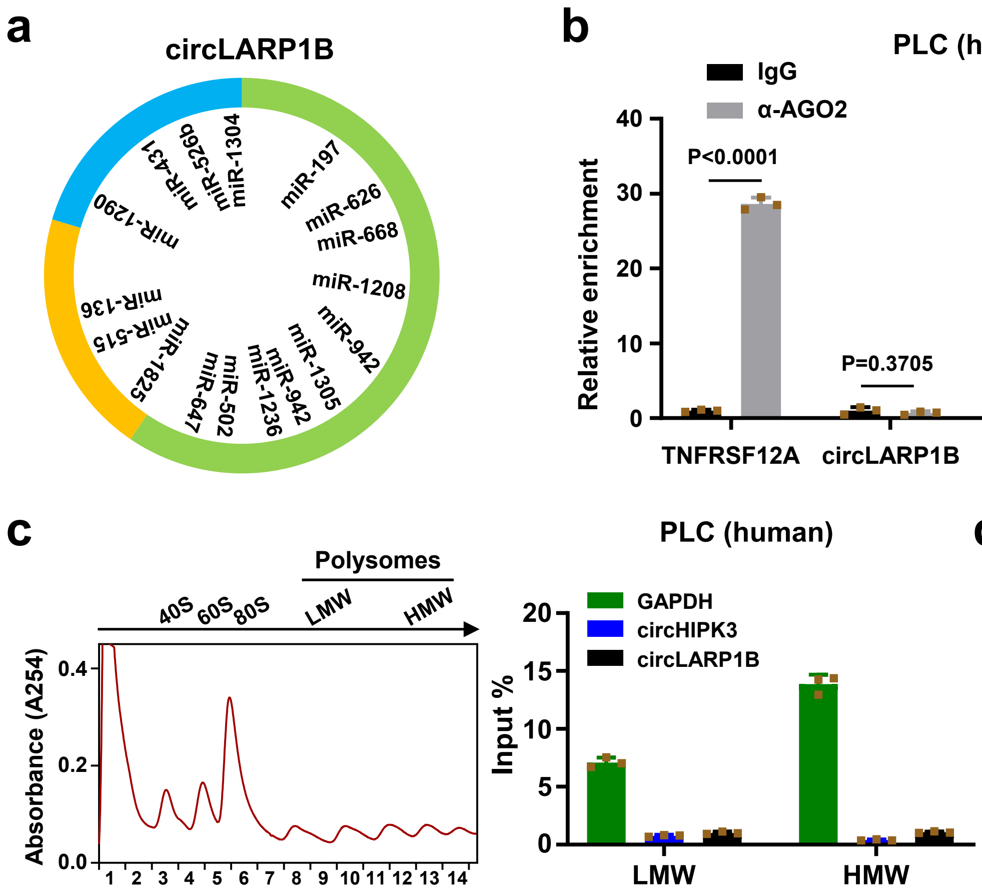

d

Coding possibility

Symbol	ORF	Score
human LARP1B mRNA	1008 nt	0.9993
human circLARP1B	84 nt	0.0168
murine Larp1b mRNA	1626 nt	0.9999
murine circLARP1B	84 nt	0.0150

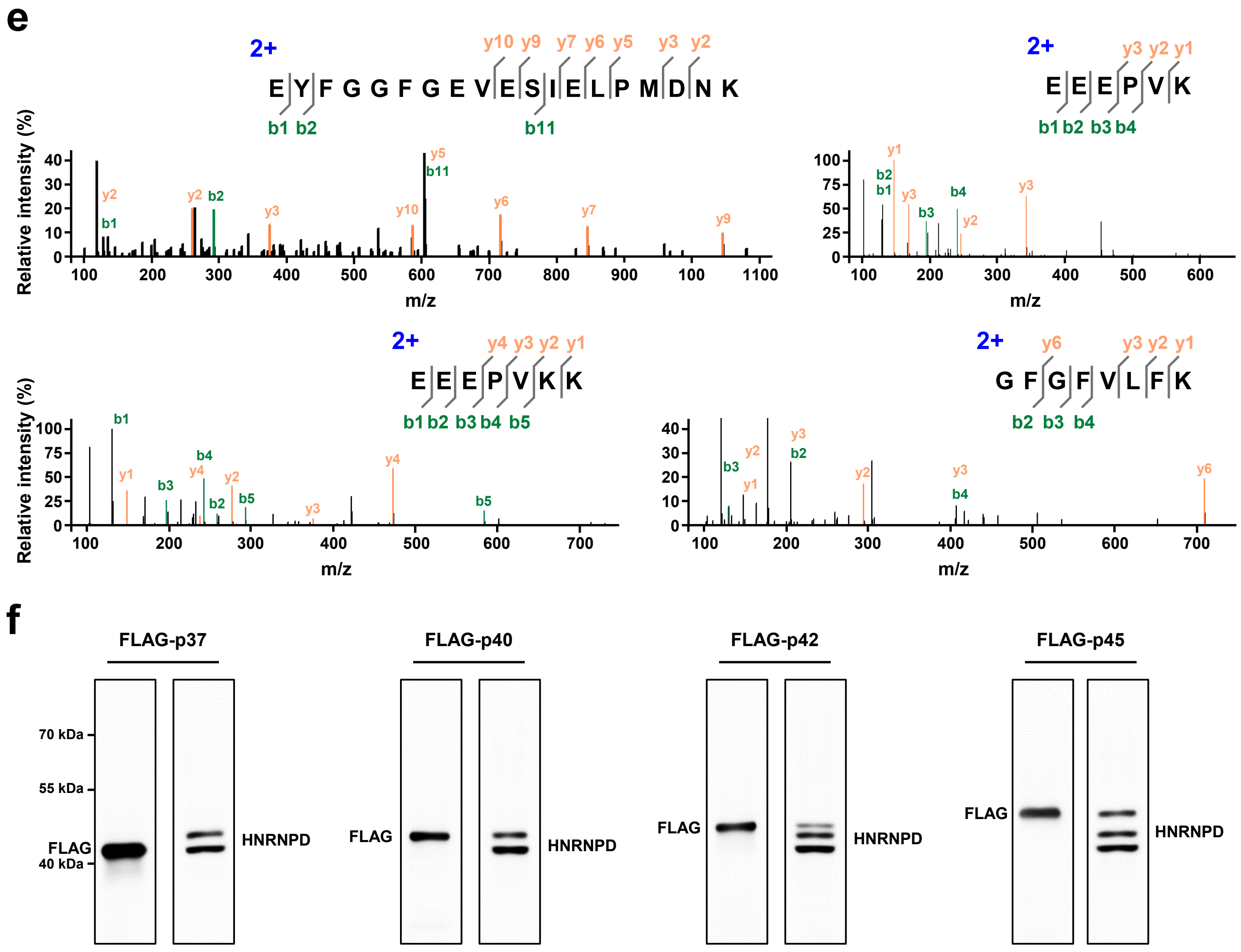

Figure S4. Examination of potential circLARP1B-interacting molecules. a) The putative miRNA binding sites of *circLARP1B* predicted with CircInteractome.[46] b) AGO2 RIP assay with PLC cells followed by the detection of RNAs in the RIP materials. *TNFRSF12A* mRNA, a positive control, is known to be targeted by miRNAs loaded in AGO2.[95] while EIciPAIP2, a negative control, is nuclear localized circRNA.[17] Western blotting showing the successful AGO2 IP. c) Ribosome profiling curve. LMW, low molecular weight fraction; HMW, high molecular weight fraction. RT-qPCR of *circLARP1B* in either LMW or HMW. *GAPDH* mRNA is a positive control, and *circHIPK3*, a negative control, is known to be noncoding.[96] d) The coding possibilities of human and murine *circLARP1B* predicted by CPAT.[47] Generally, a coding score over 0.5 is required to be considered as potentially coding. e) The identified peptides of HNRNPD from MS assay. HNRNPD was the only protein identified from MS analysis of the band around 40 kDa in sliver staining. f) Four HNRNPD variants (p37, p40, p42, and p45) examined by western blotting with anti-FLAG and anti-HNRNPD antibodies when PLC cells were transfected with FLAG-tagged HNRNPD isoforms. For (b) and (c), Data are shown as mean ± SD from three independent experiments. For (b), *P* values by two-tailed unpaired Student's *t*-test.

Figure S5

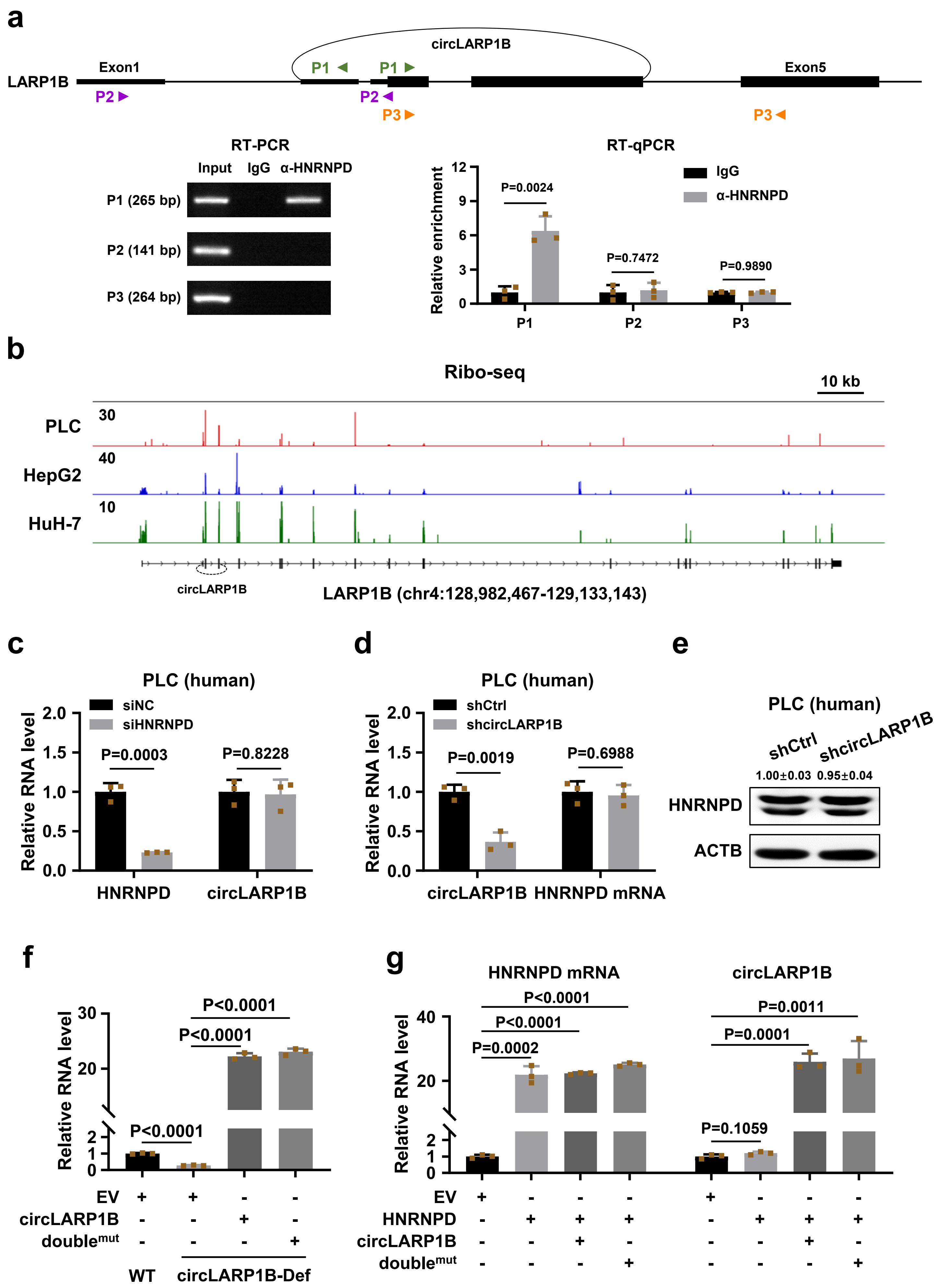
a
circLARP1B
LARP1B
Exon1
Exon5
P1
P2
P3
RT-PCR
Input
IgG
α-HNRNPD
P1 (265 bp)
P2 (141 bp)
P3 (264 bp)
RT-qPCR
Relative enrichment
IgG
α-HNRNPD
P=0.0024
P=0.7472
P=0.9890
b
Ribo-seq
10 kb
PLC
HepG2
HuH-7
30
40
10
circLARP1B
LARP1B (chr4:128,982,467-129,133,143)
c
PLC (human)
siNC
siHNRNPD
Relative RNA level
P=0.0003
P=0.8228
HNRNPD
circLARP1B
d
PLC (human)
shCtrl
shcircLARP1B
P=0.0019
P=0.6988
circLARP1B
HNRNPD mRNA
e
PLC (human)
shCtrl
shcircLARP1B
1.00±0.03
0.95±0.04
HNRNPD
ACTB
f
P<0.0001
P<0.0001
P<0.0001
EV
circLARP1B
double^mut
WT
circLARP1B-Def
g
HNRNPD mRNA
circLARP1B
P<0.0001
P<0.0001
P=0.0002
P=0.0011
P=0.0001
P=0.1059
EV
HNRNPD
circLARP1B
double^mut

Figure S5. Reciprocal modulation of *circLARP1B* and HNRNPD. a) The enrichment of indicated fragments of HNRNPD RIP. One pair divergent primers (P1) are specific for *circLARP1B* amplification. Two pairs of convergent primers (P2 and P3) are specific for the amplification of *LARP1B* mRNA. Gel images demonstrate the semi-quantitative RT-PCR products of RIP RNAs. RT-qPCR analyses show the enrichment of RIP RNAs. b) Ribo-seq signals of PLC (GSE147840), HepG2 (GSE125757) and HuH-7 (GSE128320) cells in the *LARP1B* genomic loci. c) RT-qPCR of *circLARP1B* upon HNRNPD silencing in PLC cells. siNC, siRNA negative control with scrambled sequences; siHNRNPD, siRNA against human *HNRNPD* mRNA. d) RT-qPCR of *HNRNPD* mRNA expression upon *circLARP1B* silencing in PLC cells. shcircLARP1B, shRNA against the *circLARP1B* BSJ; shCtrl, shRNA control gives rise to siRNA with scrambled sequences. e) Western blotting of HNRNPD protein upon *circLARP1B* silencing in PLC cells. The grey-scale statistics of western blotting was performed by Image J. f) RT-qPCR of *circLARP1B* in WT or circLARP1B-Def cells under the overexpression of *circLARP1B* or double^{mut} *circLARP1B*. g) RT-qPCR of *circLARP1B* and *HNRNPD* mRNA in PLC cells with the overexpression of the corresponding transcript. In (f) and (g), EV, empty vector. For (a) and (c-g), Data are shown as mean ± SD from three independent experiments. For (a), (c), (d), (f) and (g), *P* values by two-tailed unpaired Student's *t*-test.

Figure S6

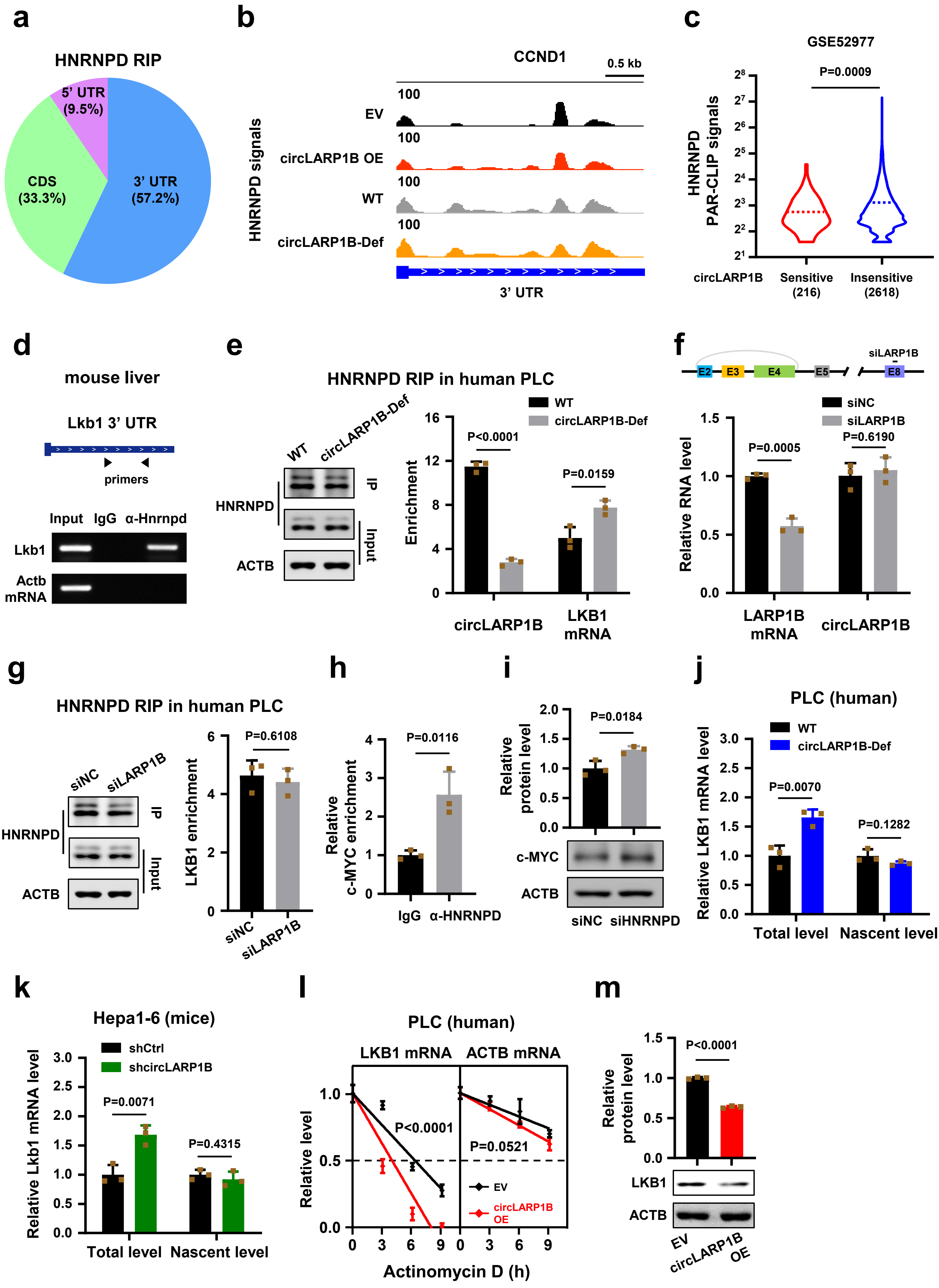

a
HNRNPD RIP
5' UTR (9.5%)
CDS (33.3%)
3' UTR (57.2%)
b
CCND1
0.5 kb
HNRNPD signals
EV
circLARP1B OE
WT
circLARP1B-Def
100
3' UTR
c
GSE52977
HNRNPD PAR-CLIP signals
P=0.0009
circLARP1B
Sensitive (216)
Insensitive (2618)
d
mouse liver
Lkb1 3' UTR
primers
Input IgG α-Hnrnpd
Lkb1
Actb mRNA
e
HNRNPD RIP in human PLC
WT circLARP1B-Def
HNRNPD
IP
Input
ACTB
Enrichment
P<0.0001
P=0.0159
circLARP1B
LKB1 mRNA
f
siLARP1B
E2 E3 E4 E5 E8
siNC
siLARP1B
Relative RNA level
P=0.0005
P=0.6190
LARP1B mRNA
circLARP1B
g
HNRNPD RIP in human PLC
siNC siLARP1B
HNRNPD
IP
Input
ACTB
LKB1 enrichment
P=0.6108
h
Relative c-MYC enrichment
P=0.0116
IgG α-HNRNPD
i
Relative protein level
P=0.0184
c-MYC
ACTB
siNC siHNRNPD
j
PLC (human)
WT
circLARP1B-Def
Relative LKB1 mRNA level
P=0.0070
P=0.1282
Total level
Nascent level
k
Hepa1-6 (mice)
shCtrl
shcircLARP1B
Relative Lkb1 mRNA level
P=0.0071
P=0.4315
Total level
Nascent level
l
PLC (human)
LKB1 mRNA
ACTB mRNA
Relative level
P<0.0001
P=0.0521
EV
circLARP1B OE
Actinomycin D (h)
m
Relative protein level
P<0.0001
LKB1
ACTB
EV
circLARP1B OE

Figure S6. *CircLARP1B* destabilizes *LBK1* mRNA in human and mice. a) Distribution of HNRNPD RIP-seq mRNA targets identified. RIP is with the cytoplasm of PLC cells. b) HNRNPD binding signals on the 3' UTR of *CCND1* mRNA in the corresponding cells. *CCND1* mRNA is a known binding target of HNRNPD.[50] c) HNRNPD PAR-CLIP signals (GSE52977) in the 3' UTRs of 216 *circLARP1B* sensitive targets and 2618 other HNRNPD targets. d) RT-PCR showing the successful pull-down of *Lkb1* 3' UTR by Hnrnpd RIP in mouse liver. e) Association of *circLARP1B* and *LKB1* mRNA examined by RT-qPCR of HNRNPD RIP in WT and circLARP1B-Def PLC cells. Western blot images indicate successful IP of HNRNPD protein. Enrichment, normalized to IgG. f) RT-qPCR of *LARP1B* mRNA and *circLARP1B* in PLC cells treated with siLARP1B or siNC. siLARP1B, siRNA against the human *LARP1B* mRNA. g) Association of *LKB1* mRNA examined by RT-qPCR of HNRNPD RIP in PLC cells treated with siLARP1B. Western blot images indicate successful IP of HNRNPD protein. Enrichment, normalized to IgG. h) Association of *c-MYC* mRNA examined by RT-qPCR of HNRNPD RIP in PLC cells. i) The steady c-MYC protein levels examined by western blotting in PLC cells upon HNRNPD knockdown. siHNRNPD, siRNA against the human *HNRNPD* mRNA. j) RT-qPCR analysis of the steady levels and nascent levels (with Nuclear Run-on assay) of *LKB1* mRNA in WT and circLARP1B-Def PLC cells. k) RT-qPCR analysis of the steady levels and nascent levels (with Nuclear Run-on assay) of *Lkb1* mRNA in Hepa1-6 cells treated with shcircLARP1B or shCtrl. shCtrl, shRNA control that gives rise to siRNA with scrambled sequences; shcircLARP1B, shRNA against the murine *circLARP1B* BSJ. l, m) Stability assay of *LKB1* mRNA and the steady levels of LKB1 protein (examined by western blotting) in PLC cells upon *circLARP1B* overexpression. EV, empty vector. For (f), (g) and (i), siNC, negative control siRNA with scrambled sequences. For (i) and (m), the grey-scale statistics of western blotting was performed by Image J. For (c), *P* values by two-tailed unpaired *t*-test. For (e-k) and (m), data are shown as mean ± SD from three independent experiments; *P* values by two-tailed unpaired Student's *t*-test. For (l), *P* values by two-way ANOVA test.

Figure S7

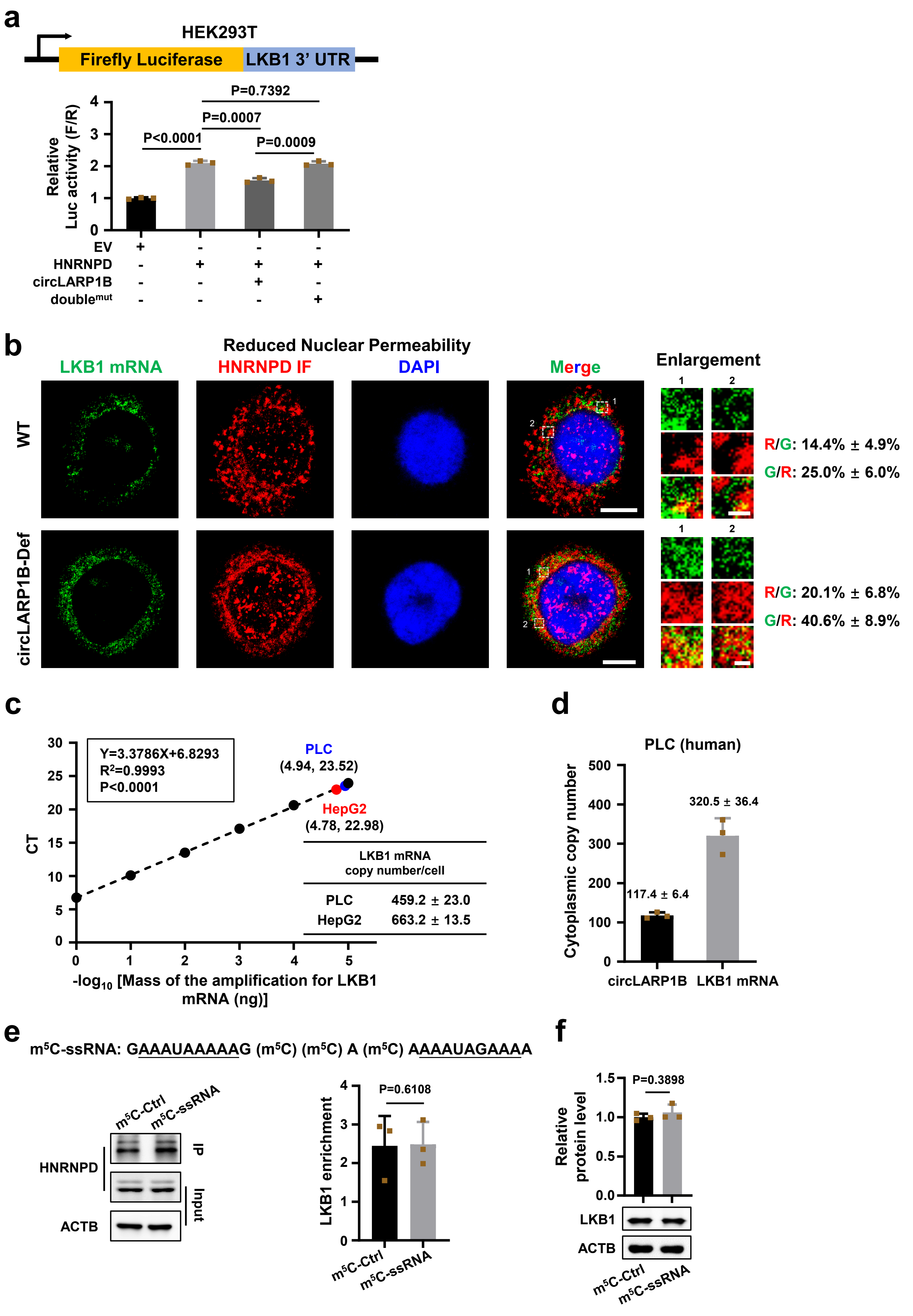
a
HEK293T
Firefly Luciferase
LKB1 3' UTR
Relative Luc activity (F/R)
P<0.0001
P=0.0007
P=0.7392
P=0.0009
EV
HNRNPD
circLARP1B
double^mut
b
Reduced Nuclear Permeability
LKB1 mRNA
HNRNPD IF
DAPI
Merge
Enlargement
WT
circLARP1B-Def
R/G: 14.4% ± 4.9%
G/R: 25.0% ± 6.0%
R/G: 20.1% ± 6.8%
G/R: 40.6% ± 8.9%
c
Y=3.3786X+6.8293
R²=0.9993
P<0.0001
PLC
(4.94, 23.52)
HepG2
(4.78, 22.98)
CT
-log10 [Mass of the amplification for LKB1 mRNA (ng)]
LKB1 mRNA copy number/cell
PLC 459.2 ± 23.0
HepG2 663.2 ± 13.5
d
PLC (human)
Cytoplasmic copy number
117.4 ± 6.4
320.5 ± 36.4
circLARP1B
LKB1 mRNA
e
m5C-ssRNA: GAAAUAAAAAG (m5C) (m5C) A (m5C) AAAAUAGAAAA
m5C-Ctrl
m5C-ssRNA
HNRNPD
ACTB
IP
Input
LKB1 enrichment
P=0.6108
f
Relative protein level
P=0.3898
LKB1
ACTB

Figure S7. *CircLARP1B* destabilizes *LBK1* mRNA in human. a) Dual-luciferase assay showing the effect of *circLARP1B* on the stability of transcript with *LKB1* 3' UTR. The 3' UTR of human *LKB1* mRNA is served as the 3' UTR of firefly luciferase mRNA. The corresponding HNRNPD protein or circRNA is overexpressed. b) Representative images of *LKB1* mRNA FISH together with HNRNPD IF in WT and circLARP1B-Def PLC cells. A permeabilization condition was used to reduce the nuclear HNRNPD signals,[59,60] thereby highlighting cytoplasmic signals in PLC cells. Boxed areas are enlarged. The colocalization between *LKB1* mRNA (G, green) and HNRNPD (R, red) is shown (N=15 randomly selected areas). R/G, proportion of red signal to green signal colocalization; G/R, proportion of green signal to red signal colocalization. IF, immunofluorescence. Scale bar, 10 μm and 1 μm (enlarged areas). c) Quantification of *LKB1* mRNA copy numbers in HepG2 and PLC cell lines. The red and blue dots present the Ct values and the amount of *LKB1* mRNA from the cDNA used in HepG2 and PLC cells, respectively. The inset presents the copy numbers of *LKB1* mRNA in HepG2 and PLC cells. d) Quantification of cytoplasmic *circLARP1B* and *LKB1* mRNA copy numbers in PLC cells. e) Association of *LKB1* mRNA examined by RT-qPCR of HNRNPD RIP in PLC cells treated with m^5C-Ctrl or m^5C-ssRNA. Western blot images indicate successful IP of HNRNPD protein (right). m^5C-ssRNA, m^5C modified single-strand RNA with the HNRNPD binding motifs which were indicated with underlines; m^5C-Ctrl, negative control m^5C modified ssRNA with scrambled sequences; m^5C, 5-methylcytosine. Enrichment, normalized to IgG. f) Western blotting and the corresponding quantification showing the steady level of LKB1 protein in PLC cells transfected with m^5C-Ctrl or m^5C-ssRNA. The grey-scale statistics of western blotting was performed by Image J. For (a) and (c-f), data are shown as mean ± SD from three independent experiments. For (a), (e) and (f), *P* values by two-tailed unpaired Student's *t*-test.

Figure S8

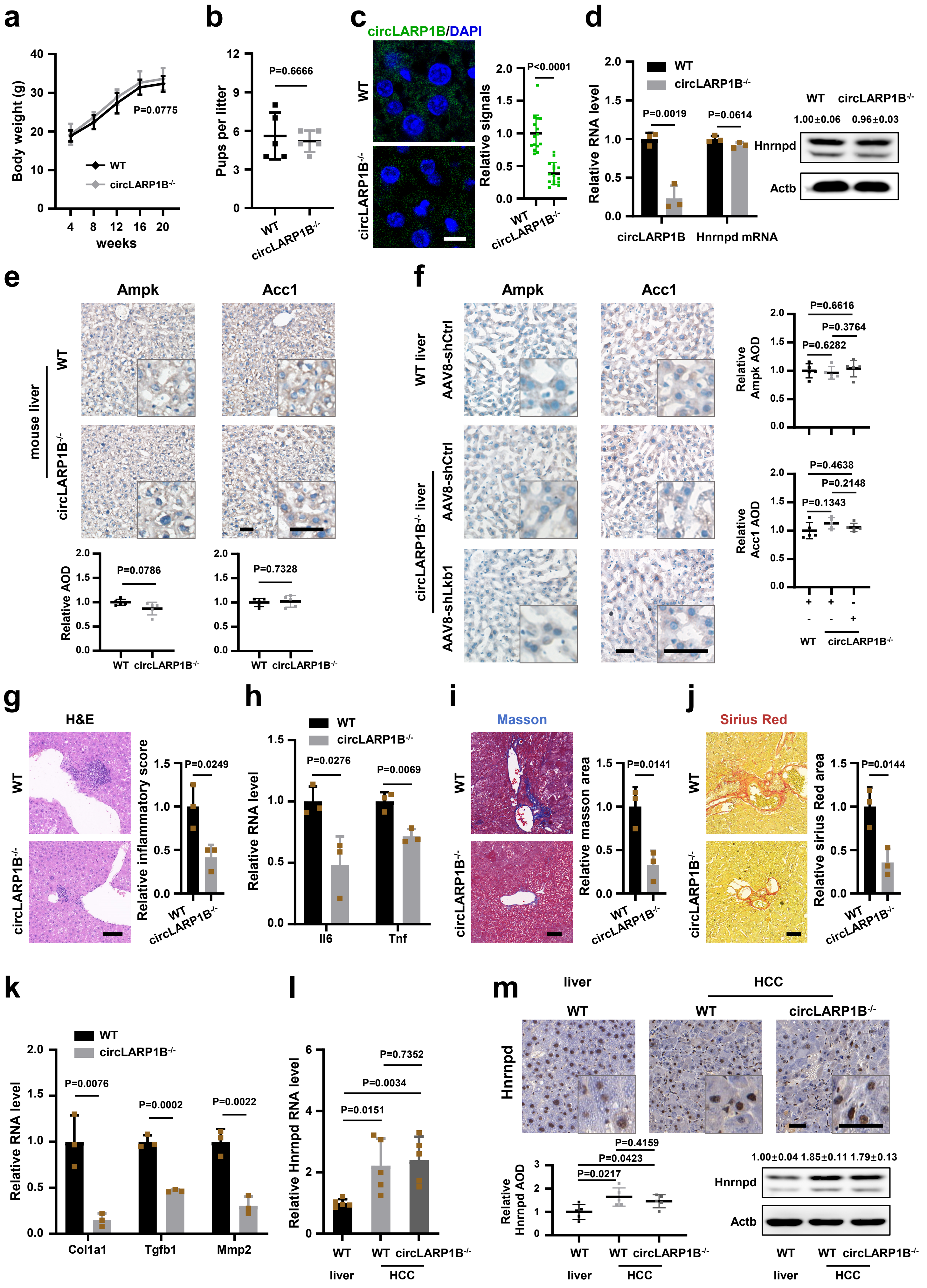

a
Body weight (g)
weeks
P=0.0775
WT
circLARP1B-/-
b
Pups per litter
P=0.6666
c
circLARP1B/DAPI
Relative signals
P<0.0001
d
Relative RNA level
P=0.0019
P=0.0614
circLARP1B
Hnrnpd mRNA
1.00±0.06
0.96±0.03
Hnrnpd
Actb
e
Ampk
Acc1
mouse liver
Relative AOD
P=0.0786
P=0.7328
f
WT liver
circLARP1B-/- liver
AAV8-shCtrl
AAV8-shLkb1
Relative Ampk AOD
P=0.6616
P=0.3764
P=0.6282
Relative Acc1 AOD
P=0.4638
P=0.2148
P=0.1343
g
H&E
Relative inflammatory score
P=0.0249
h
P=0.0276
P=0.0069
Il6
Tnf
i
Masson
Relative masson area
P=0.0141
j
Sirius Red
Relative sirius Red area
P=0.0144
k
P=0.0076
P=0.0002
P=0.0022
Col1a1
Tgfb1
Mmp2
l
Relative Hnrnpd RNA level
P=0.7352
P=0.0034
P=0.0151
liver
HCC
m
Relative Hnrnpd AOD
P=0.4159
P=0.0423
P=0.0217
1.00±0.04 1.85±0.11 1.79±0.13

Figure S8. Examination of liver of *circLARP1B*$^{-/-}$ mice. a, b) The body weight and litter size of wild-type (WT) and *circLARP1B*$^{-/-}$ mice (N=5 per group). c) Representative smFISH of *circLARP1B* (green) and the quantification from WT and *circLARP1B*$^{-/-}$ mouse liver. N=15 cells analyzed. Scale bar, 10 μm. d) RT-qPCR of *Hnrnpd* mRNA and Western blot of Hnrnpd protein in WT and *circLARP1B*$^{-/-}$ mouse liver. Data are from three independent experiments. e) Representative IHC staining and the corresponding quantification of the Ampk and Acc1 proteins in livers from WT and *circLARP1B*$^{-/-}$ mice (N=5 per group). Scale bar, 50 μm. f) Representative IHC staining and the corresponding quantification of the Ampk and Acc1 proteins in livers from WT or *circLARP1B*$^{-/-}$ mice (N=5 per group) with the intravenous tail injection of AAV8-shCtrl or AAV8-shLkb1 for 3 weeks. Scale bar, 50 μm. g) Representative images and quantification of H&E staining of livers from WT and *circLARP1B*$^{-/-}$ mice (N=3 per group) after DEN-injection for 10 weeks. Scale bar, 100 μm. h) RT-qPCR analysis of mRNA levels of Il6 and Tnf (two pro-inflammation markers) in livers from WT and *circLARP1B*$^{-/-}$ mice (N=3 per group) after DEN-injection for 10 weeks. i, j) Representative images and quantification of Masson (i), and Sirius Red (j) staining of livers from WT and *circLARP1B*$^{-/-}$ mice (N=3 per group) after DEN-injection for 10 weeks. Scale bar, 50 μm. k) RT-qPCR analysis of mRNA levels of fibrosis markers (Col1a1, Tgfb1 and Mmp2) in the livers from WT and *circLARP1B*$^{-/-}$ mice (N=3 per group) after DEN-injection for 10 weeks. l) RT-qPCR analysis of *Hnrnpd* mRNA levels in WT mice livers and liver tumors from WT and *circLARP1B*$^{-/-}$ HCC mice (N=5 per group). m) IHC and western blotting analysis of Hnrnpd protein levels in WT mice livers and liver tumors from WT and *circLARP1B*$^{-/-}$ HCC mice (N=5 per group). Scale bar, 50 μm. All animals were kept in a controlled environment (23–25 °C with a 12-hour light-dark cycle and lights on at 8:00 AM), on normal diet feeding with free access to water, and all data were from mice at 9:00 AM (a-k), or fasted for 12 hours before bleeding and sacrifice at 9:00 AM (l and m). For (d) and (m), the grey-scale statistics of western blotting was performed by Image J. For (e), (f) and (m), the IHC signal is defined as the average optical density (AOD) calculated by Image J. For (a-m), data are shown as mean ± SD. For (a), *P* values by two-way ANOVA. For (b-m), *P* values by two-tailed unpaired Student's *t*-test.

Figure S9

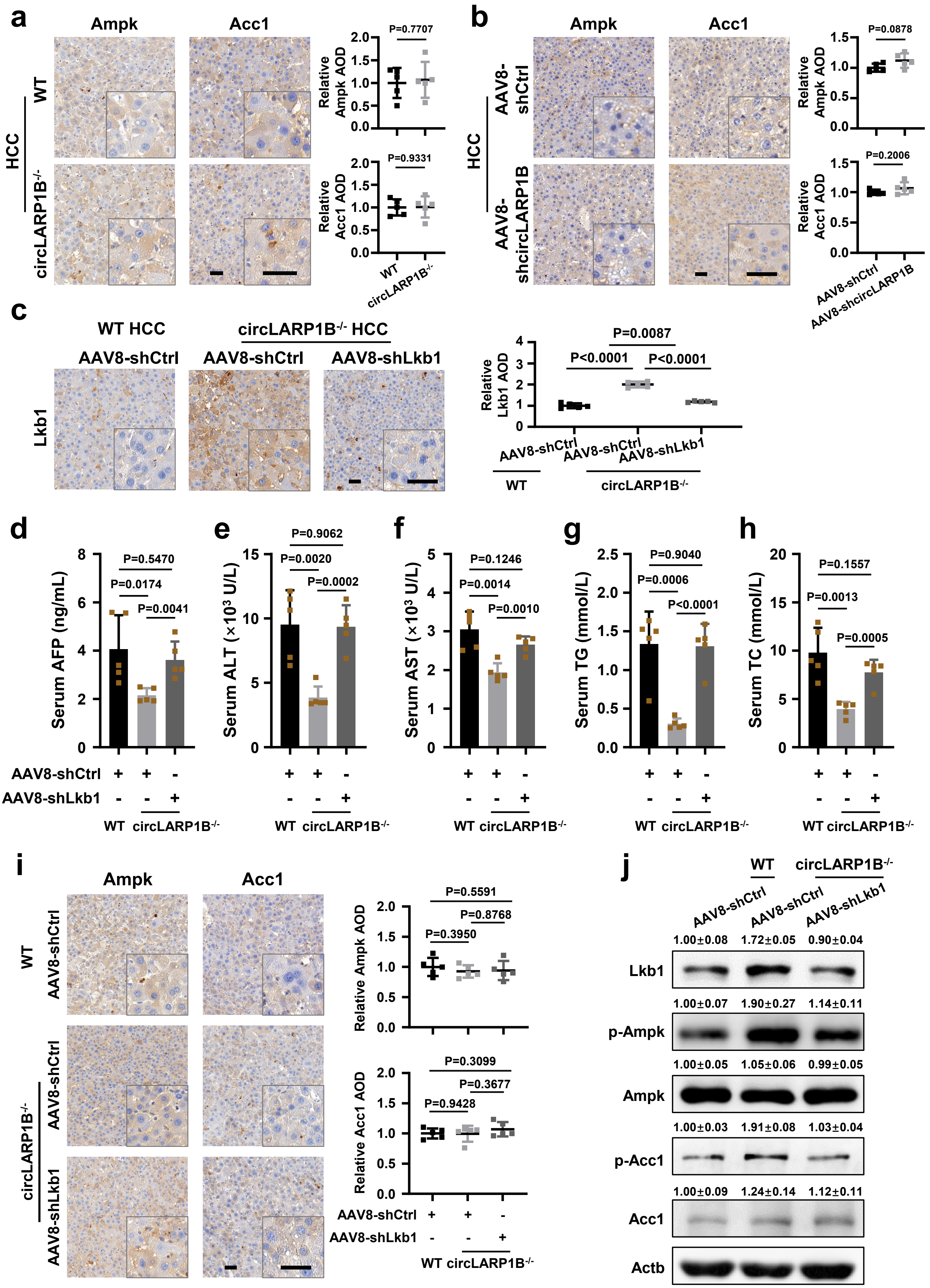
a
Ampk
Acc1
HCC
WT
circLARP1B-/-
P=0.7707
Relative Ampk AOD
P=0.9331
Relative Acc1 AOD
WT
circLARP1B-/-
b
Ampk
Acc1
HCC
AAV8-shCtrl
AAV8-shcircLARP1B
P=0.0878
Relative Ampk AOD
P=0.2006
Relative Acc1 AOD
AAV8-shCtrl
AAV8-shcircLARP1B
c
WT HCC
circLARP1B-/- HCC
AAV8-shCtrl
AAV8-shCtrl
AAV8-shLkb1
Lkb1
P=0.0087
P<0.0001
P<0.0001
Relative Lkb1 AOD
AAV8-shCtrl
AAV8-shCtrl
AAV8-shLkb1
WT
circLARP1B-/-
d
Serum AFP (ng/mL)
P=0.5470
P=0.0174
P=0.0041
e
Serum ALT (×10³ U/L)
P=0.9062
P=0.0020
P=0.0002
f
Serum AST (×10³ U/L)
P=0.1246
P=0.0014
P=0.0010
g
Serum TG (mmol/L)
P=0.9040
P=0.0006
P<0.0001
h
Serum TC (mmol/L)
P=0.1557
P=0.0013
P=0.0005
AAV8-shCtrl + + -
AAV8-shLkb1 - - +
WT circLARP1B-/-
i
Ampk
Acc1
WT
AAV8-shCtrl
circLARP1B-/-
AAV8-shCtrl
AAV8-shLkb1
P=0.5591
P=0.8768
P=0.3950
Relative Ampk AOD
P=0.3099
P=0.3677
P=0.9428
Relative Acc1 AOD
AAV8-shCtrl + + -
AAV8-shLkb1 - - +
WT circLARP1B-/-
j
WT
circLARP1B-/-
AAV8-shCtrl
AAV8-shCtrl
AAV8-shLkb1
Lkb1 1.00±0.08 1.72±0.05 0.90±0.04
p-Ampk 1.00±0.07 1.90±0.27 1.14±0.11
Ampk 1.00±0.05 1.05±0.06 0.99±0.05
p-Acc1 1.00±0.03 1.91±0.08 1.03±0.04
Acc1 1.00±0.09 1.24±0.14 1.12±0.11
Actb

Figure S9. LKB1 is a key target for the roles of *circLARP1B* in HCC mice. a) Representative Ampk and Acc1 IHC staining of liver tumors from WT and *circLARP1B*$^{-/-}$ HCC mice (N=5 per group). Scale bar, 50 μm. b) Representative Ampk and Acc1 IHC staining of liver tumors from WT mice (N=5 per group) with AAV8-shCtrl or AAV8-shcircLARP1B injection. Scale bar, 50 μm. c) Representative Lkb1 IHC staining of liver tumors from WT and *circLARP1B*$^{-/-}$ HCC mice (N=5 per group) with AAV8-shCtrl or AAV8-shLkb1 injection. The corresponding quantification is shown. Scale bar, 50 μm. d-h) The serum AFP (d), ALT (e), AST (f), TG (g) and TC (h) levels in WT and *circLARP1B*$^{-/-}$ HCC mice (N=5 per group) with AAV8-shCtrl or AAV8-shLkb1 injection. i) Representative Ampk and Acc1 IHC staining of liver tumors from WT and *circLARP1B*$^{-/-}$ mice (N=5 per group) with AAV8-shCtrl or AAV8-shLkb1 injection (left). The corresponding quantification is shown (right). Scale bar, 50 μm. j) Western blot of the indicated proteins of liver tumors from WT and *circLARP1B*$^{-/-}$ mice (N=5 per group) with AAV8-shCtrl or AAV8-shLkb1 injection. The grey-scale statistics of western blotting was performed by Image J. All animals were kept in a controlled environment (23–25 °C with a 12-hour light-dark cycle and lights on at 8:00 AM), on normal diet feeding with free access to water, and fasted for 12 hours before bleeding and sacrifice at 9:00 AM. For (a), (b), (c) and (i), the IHC signal is defined as the average optical density (AOD) calculated by Image J. For (a-i), Data are shown as mean ± SD; *P* values by two-tailed unpaired Student's *t*-test.

Figure S10

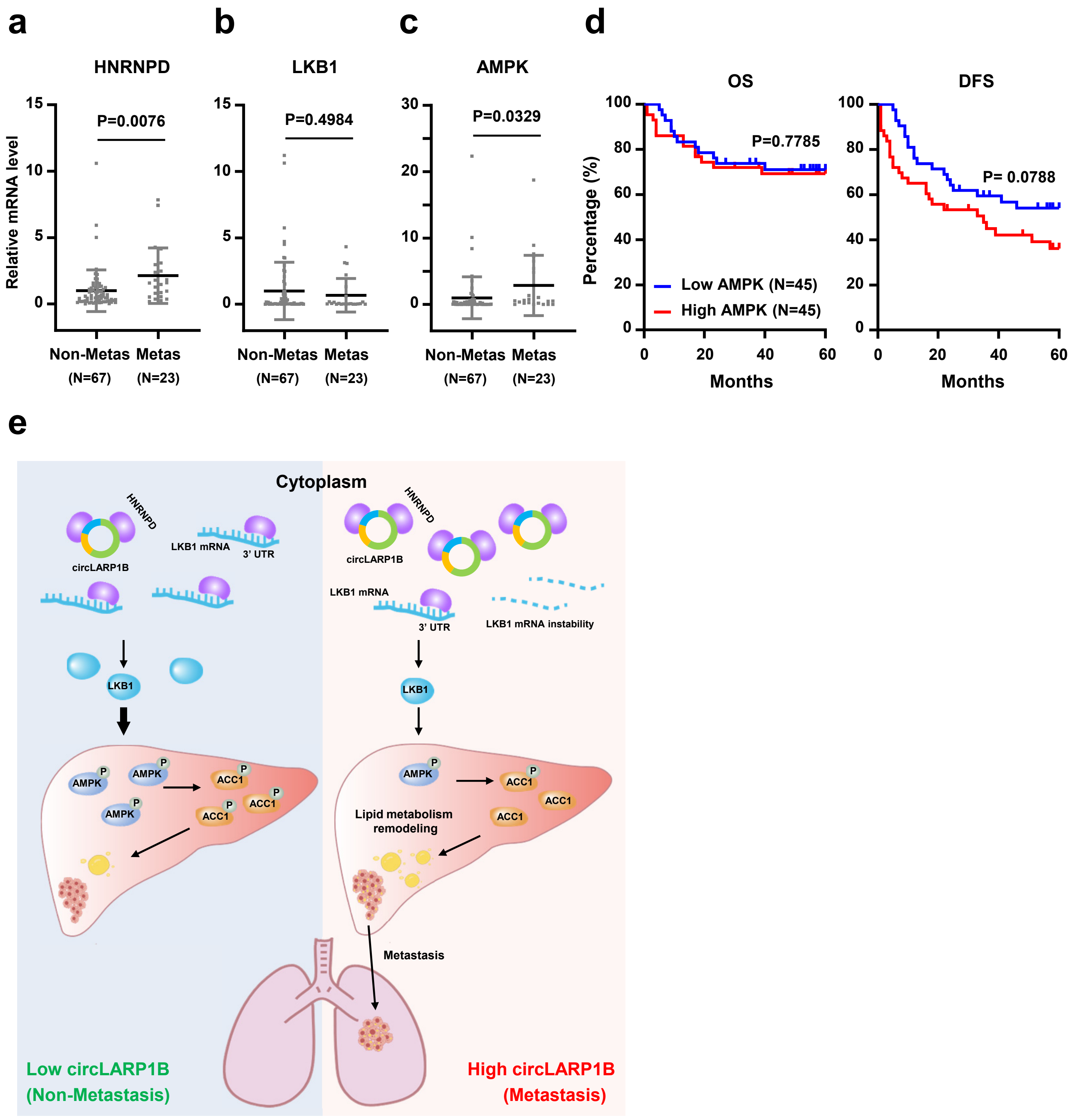

a
HNRNPD
Relative mRNA level
P=0.0076
Non-Metas
Metas
(N=67)
(N=23)
b
LKB1
P=0.4984
Non-Metas
Metas
(N=67)
(N=23)
c
AMPK
P=0.0329
Non-Metas
Metas
(N=67)
(N=23)
d
OS
DFS
Percentage (%)
P=0.7785
P= 0.0788
Low AMPK (N=45)
High AMPK (N=45)
Months
Months
e
Cytoplasm
HNRNPD
circLARP1B
LKB1 mRNA
3' UTR
LKB1 mRNA instability
LKB1
AMPK
P
ACC1
Lipid metabolism remodeling
Metastasis
Low circLARP1B
(Non-Metastasis)
High circLARP1B
(Metastasis)

Figure S10. Clinical significance of the HNRNPD-LKB1-AMPK axis and the working model. a-c) RT-qPCR analysis of *HNRNPD* (a), *LKB1* (b) and *AMPK* (c) mRNA expressions in HCC specimens. *ACTB* mRNA is the endogenous control for normalization. *P* values are from two-tailed unpaired Student's *t*-test. d) Kaplan–Meier analysis of OS and DFS for 90 HCC patients with *AMPK* mRNA levels. The red curve indicates survival in patients with higher HCC *AMPK* mRNA levels, and the blue line indicates survival in patients with lower HCC *AMPK* mRNA levels. *P* values are calculated by the log-rank test. e) A brief summary and a working model for roles and functional mechanisms of *circLARP1B* in HCC lipid metabolism and metastasis.
